# Quantitative proteomics and phosphoproteomics of urinary extracellular vesicles define putative diagnostic biosignatures for Parkinson’s disease

**DOI:** 10.1038/s43856-023-00294-w

**Published:** 2023-05-10

**Authors:** Marco Hadisurya, Li Li, Kananart Kuwaranancharoen, Xiaofeng Wu, Zheng-Chi Lee, Roy N. Alcalay, Shalini Padmanabhan, W. Andy Tao, Anton Iliuk

**Affiliations:** 1 Department of Biochemistry, Purdue University, West Lafayette, IN 47907 USA; 2 Tymora Analytical Operations, West Lafayette, IN 47906 USA; 3 School of Electrical and Computer Engineering, Purdue University, West Lafayette, IN 47907 USA; 4 Department of Chemistry, Purdue University, West Lafayette, IN 47907 USA; 5 West Lafayette Junior/Senior High School, West Lafayette, IN 47906 USA; 6 Department of Neurology, Columbia University Irving Medical Center, New York, NY 10032 USA; 7 The Michael J. Fox Foundation for Parkinson’s Research, New York City, NY 10163 USA; 8 Department of Medicinal Chemistry and Molecular Pharmacology, Purdue University, West Lafayette, IN 47907 USA; 9 Purdue Institute for Cancer Research, Purdue University, West Lafayette, IN 47907 USA

**Keywords:** Parkinson's disease, Proteomics

## Abstract

**Background:**

Mutations in the leucine-rich repeat kinase 2 (*LRRK2*) gene have been recognized as genetic risk factors for Parkinson’s disease (PD). However, compared to cancer, fewer genetic mutations contribute to the cause of PD, propelling the search for protein biomarkers for early detection of the disease.

**Methods:**

Utilizing 138 urine samples from four groups, healthy individuals (control), healthy individuals with G2019S mutation in the *LRRK2* gene (non-manifesting carrier/NMC), PD individuals without G2019S mutation (idiopathic PD/iPD), and PD individuals with G2019S mutation (LRRK2 PD), we applied a proteomics strategy to determine potential diagnostic biomarkers for PD from urinary extracellular vesicles (EVs).

**Results:**

After efficient isolation of urinary EVs through chemical affinity followed by mass spectrometric analyses of EV peptides and enriched phosphopeptides, we identify and quantify 4476 unique proteins and 2680 unique phosphoproteins. We detect multiple proteins and phosphoproteins elevated in PD EVs that are known to be involved in important PD pathways, in particular the autophagy pathway, as well as neuronal cell death, neuroinflammation, and formation of amyloid fibrils. We establish a panel of proteins and phosphoproteins as novel candidates for disease biomarkers and substantiate the biomarkers using machine learning, ROC, clinical correlation, and in-depth network analysis. Several putative disease biomarkers are further partially validated in patients with PD using parallel reaction monitoring (PRM) and immunoassay for targeted quantitation.

**Conclusions:**

These findings demonstrate a general strategy of utilizing biofluid EV proteome/phosphoproteome as an outstanding and non-invasive source for a wide range of disease exploration.

## Plain language summary

Parkinson’s disease (PD) is a progressive neurological disorder that affects body movement because some brain cells stop producing the chemical dopamine. PD is often not diagnosed until it has advanced, making early detection crucial. To enable early detection, we investigated tiny packages called extracellular vesicles released from a variety of cells, including the brain cells, that can be found in urine as a potential source for diagnosing PD. These tiny packages contain different kinds of molecules from inside the cells. We analyzed urine samples from 138 individuals and found several proteins involved in PD development that could be biological indicators for early detection of the disease. We used various techniques to make sure that our findings were accurate. Our study suggests that looking at these proteins in urine could be a good way to detect PD in a non-invasive manner.

## Abstract (web summary)

Hadisurya et al. identify potential diagnostic biomarkers for Parkinson’s disease (PD) via mass spectrometry analysis of urinary extracellular vesicles. The authors identify multiple proteins and phosphoproteins previously found to be involved in PD pathophysiology.

## Introduction

It has been more than two centuries since Parkinson’s disease (PD) was described by Dr. Parkinson in 1817^1^. PD is the second most common neurogenerative disorder after Alzheimer’s disease (AD)^2^. PD’s most common pathological finding is a decreased pigmentation in the substantia nigra pars compacta (SNpc) caused by the death of dopaminergic neurons, leading to progressive deterioration of motor function^3,4^. In addition to motor symptoms, non-motor symptoms may include cognitive impairment, autonomic dysfunction, hyposmia, and sleep disturbances^5^. Currently, PD is incurable and progresses gradually with symptom deterioration into severe disabilities^6^. It has been estimated that PD affects 1 percent of the population over 60^7^. Overall, as many as 1 million Americans are living with PD, and approximately 60,000 Americans are diagnosed with PD each year^8,9^.

While the cause of PD is currently unknown, researchers speculate that environmental and genetic factors contribute to its development^10^. Large-scale genome-wide association studies (GWAS) have identified 41 independent risk variants for PD in various cohorts^4,11^. A subset of patients develops PD because of a major genetic risk. Specifically, mutations in the Leucine-rich repeat kinase 2 (*LRRK2*) gene are found in hereditary forms, emphasizing the shared molecular pathway driving both familial and non-familial PD to comprise the most common cause of the disease^12,13^. Mutations in *LRRK2* have been recognized as genetic risk factors for sporadic (~1%) and familial forms of PD (~5%)^13^. *LRRK2* encodes a large protein of 2527 amino acids containing two functional enzymatic domains, the GTPase and the Ser/Thr kinase domains, and several protein–protein interaction domains such as the armadillo, ankyrin, leucine-rich repeat (LRR), and WD40 domains^14,15^. Out of many mutations in *LRRK2*, Gly2019→Ser (G2019S) mutation in its kinase domain is by far the most common among caucasians^16^. Interestingly, some individuals with the G2019S mutation, known as the non-manifesting carrier (NMC) group, have not developed PD yet. Whether they will develop the disease at an older age remains unclear.

Recent findings regarding the Gly2019→Ser (G2019S) mutation in the *LRRK2* kinase domain have uncovered that the mutation drives changes in vesicular trafficking, autophagy, and lysosomal dysfunction signaling pathways^16^. The changes in these signaling pathways are attributed to the hyperactivation of the LRRK2 kinase activity assessed by phosphorylation of its substrates, the Rab proteins^17^. Rab proteins are the main regulators of important aspects of autophagy and lysosome activity, including membrane trafficking, vesicle formation, vesicle movement along actin and tubulin networks, and membrane docking and fusion. In short, from the evidence above, it is conceivable that the changes in signaling pathways caused by the Gly2019→Ser (G2019S) mutation in the *LRRK2* kinase domain may be reflected in extracellular vesicles (EVs). Therefore, EVs offer a promising source for protein biomarkers in PD.

EVs (primarily exosomes and microvesicles) are lipid bilayer-coated nanoparticles secreted by all cell types. The secretion of EVs was initially considered a means of eliminating proteins, lipids, and RNA from inside the cells^18^. With accumulating evidence, EVs have become recognized as a very important component in intercellular communication^19^. Recent studies have reported EVs as a rich resource of biomarkers for the non-invasive detection of neurodegenerative diseases from biofluids^20^. These EV-based disease markers can be identified well before the onset of symptoms or physiological detection of illness, making them promising candidates for early-stage PD diagnosis^21,22^. Moreover, since phosphorylation events directly reflect cellular physiological status during neurodegeneration, urinary EVs represent a highly promising source of phosphoproteins as non-invasive disease markers^23,24^. Previous studies from our group have identified numerous EV proteins and phosphoproteins in urine and plasma from breast cancer, chronic kidney disease, kidney cancer, and pancreatic cancer patients^25–28^.

Furthermore, some other groups already explored the possibility of using EV biofluids, such as cerebrospinal fluid (CSF), saliva, and plasma, as the source for PD diagnosis. CSF exosomes from patients with PD and dementia with Lewy bodies contain a pathogenic species of α-synuclein, which could initiate oligomerization of soluble α-synuclein in target cells and confer disease pathology^29^. Saliva-derived EVs from PD patients have elevated levels of oligomeric α-synuclein compared to controls^30^. Serum neuronal exosomes of α-synuclein and clusterin could predict and differentiate PD from atypical parkinsonism^31^. Although CSF, saliva, and plasma-derived EVs have been used for biomarker studies of PD, urine-derived EVs offer another promising clinically viable matrix for PD detection since urine can be non-invasively collected frequently in large volumes and repeatedly at different time points^32^. More importantly, although most urine-derived EVs originate from the kidney and urinary tract, a substantial proportion of those also originate from distal organs, including the brain^33,34^. Moreover, urinary EVs will reflect the whole physiological changes that happened to the body of PD patients. Therefore, urine-derived EVs may provide diagnostic opportunities for PD^35,36^.

Here we present a strategy for the discovery and development of proteins and phosphoproteins from urinary EVs as putative diagnostic biosignatures for Parkinson’s disease. For the discovery experiment, we utilized 82 individual urine samples made available from Columbia University Irving Medical Center (hereinafter referred to as “Columbia LRRK2 cohort”) under a Michael J. Fox Foundation (MJFF)-funded LRRK2 biomarker project^32^ and split them into 164 analyses (82 proteomics and 82 phosphoproteomics). We used our in-house developed unique EVtrap (Extracellular Vesicles total recovery and purification) approach to efficiently enrich EVs and coupled it with LC-MS-based detection and quantitation for accurate urinary EV proteome and phosphoproteome analysis^37^. EVtrap, based on functionalized magnetic beads with a combination of lipophilic and hydrophilic groups, has a unique affinity toward lipid bilayer membrane coating EVs. EVtrap, which binds to the lipid bilayer, enables fast and reproducible EV isolation from urine samples. Our approach successfully demonstrates the feasibility of developing biofluid-derived EV phosphoproteins for disease profiling^25,26^.

In total, we determined a panel of unique proteins and phosphoproteins as novel high-confidence candidates for disease biomarkers. Disease biomarkers will help diagnose whether a patient currently has PD. Our large-scale LC-MS analysis efforts combined with extensive bioinformatics analysis led to the discovery of unique biosignatures for potential Parkinson’s disease diagnostics. Furthermore, we also analyzed our putative biomarker involvement in important disease-relevant pathways, which might provide new information for PD intervention. These findings will enhance the discovery and development of novel EV protein-based biomarkers and help create an effective early-stage clinical diagnosis strategy for PD. An in-depth understanding of those biosignature pathways could also lead to the potential discovery of new drugs for optimal intervention strategies in PD progression.

## Methods

### Ethics/patient consent

The study was approved by Columbia University Irving Medical Center institutional review board (IRB) no. AAAP9604 and all participants signed informed consent. The study design and conduct complied with all relevant regulations regarding the use of human study participants and was conducted in accordance with the criteria set by the Declaration of Helsinki.

### Statistics and reproducibility

A total of 138 urine sample biological replicates were collected. We further divided the samples into two groups, 82 samples for the discovery experiments and 56 samples for validation experiments. For the initial comprehensive discovery experiments, the urine samples were collected from 21 healthy individuals (Control), 13 healthy individuals with G2019S mutation (non-Manifesting Carrier/NMC), 28 PD individuals without G2019S mutation (idiopathic PD/iPD), and 20 PD individuals with G2019S mutation (LRRK2 PD). The 56 urine samples used for the validation experiments were classified as 33 patients with PD and 23 healthy individuals without genetic differentiation. All 138 samples were processed separately by implementing the statistical principles in experimental designs, including replication, randomization, and blocking when applicable^38^. The unpaired Student’s two-tailed *t*-test was performed to create the volcano plots for potential biomarker discovery and functional annotation analyses. The unpaired two-sample Wilcoxon test *p*-values and the one-way ANOVA *p*-value were performed to compare the four groups on each violin plot. The unpaired two-sample Wilcoxon test *p*-values were calculated to compare gender and specific proteins.

### Sample collection

All 82 urine samples used in the discovery LC-MS study and 56 urine samples used in the validation experiments were collected at Columbia University Irving Medical Center (CUIMC) and sent to our lab blindly. The samples were collected from March 2016 to April 2017 under a Michael J. Fox Foundation (MJFF)-funded LRRK2 biomarker project^32^. Each sample has been uniquely curated for *LRRK2* genotype and PD activity effects.

### EV isolation by EVtrap

For each urine sample, approximately 10–15 mL was utilized for EV enrichment by EVtrap. Before the EVtrap capture, the urine volume was normalized based on the creatinine levels, a normalizer we found to be optimal for EV studies. The EVtrap beads were provided by Tymora Analytical Operations and were utilized and validated as described previously^26–28,37,39^. In brief, the frozen urine samples were thawed in a 37 °C water bath. The samples were then centrifuged at 2500 × *g* for 10 min to remove cell debris and large apoptotic bodies and diluted with EVtrap loading buffer in a 1:10 v/v ratio. The magnetic EVtrap beads were added directly to the diluted at a ratio of 20 µL EVtrap beads per 1 mL urine. The mixture was incubated for 1 h by end-over-end rotation, and the supernatant was removed using a magnetic separator rack; the beads were washed with PBS, and the EVs were eluted by a 10 min incubation with 100 mM triethylamine (TEA, Millipore-Sigma). The eluted samples were dried entirely in a vacuum centrifuge. For Western blot analysis, the dried EV samples were lysed directly in LDS buffer (lower volumes of urine (~0.5–2 mL) were used for Western blot experiments).

### EV isolation by differential ultracentrifugation (UC)

The EV isolation by UC was performed to compare with the EVtrap method to demonstrate the superior efficiency of isolating urinary EVs by EVtrap at the beginning of this study. Urine samples (~1–2 mL) were centrifuged at 10,000 × *g* at 4 °C for 1 h. Supernatants were further centrifuged at an ultra-high speed of 100,000 × *g* (Optima MAX-XP Ultracentrifuge, Beckman Coulter) at 4 °C for 2 h. Pellets were washed with 1x PBS and centrifuged at 100,000 × *g* for 2 h again. Collected pellets were lysed directly in LDS buffer for Western blot analysis.

### Western blot experiments

The Western blot experiments were carried out to show biological replicates (different patients) rather than technical replicates (repeats). Therefore, all of the Western blot experiments were performed only once due to a very limited amount of rare clinical samples. A small percentage (approximately 0.5 mL urine sample equivalent for CD9, 1 mL for LRRK2, and 2 mL for pSer1292-LRRK2) of each purified EV sample was incubated for 10 min at 95 °C in LDS sample buffer. The equivalent volume of each sample aliquot was loaded and separated on an SDS-PAGE gel (NuPAGE 4–12% Bis-Tris Gel, Thermo Fisher Scientific). Afterward, the proteins were transferred onto a low-fluorescence PVDF membrane (Millipore-Sigma). The membranes were cut according to the appropriate molecular weights to detect the target proteins or phosphoproteins at their corresponding molecular weights. The membrane was blocked with 1% BSA in TBST for 1 h. The cut membranes then incubated with rabbit anti-CD9 (clone D3H4P; Cell Signaling Technology) at 1:5,000 ratio, or anti-LRRK2 (clone MJFF2 (c41-2); Abcam) at 1;1000 ratio, or anti-pSer1292-LRRK2 (clone MJFR-19-7-8; Abcam) at 1:500 ratio overnight in 1% BSA in TBST (3% BSA in TBST was used for anti-pSer1292-LRRK2). For the secondary antibody visualization, Goat anti-Rabbit Alexa-Fluor 800 nm (Thermo Fisher Scientific) was used to bind the primary antibodies and incubated for 1 h in 1% BSA in TBST. Lastly, the membrane was scanned by Odyssey near-infrared scanner (Licor) for signal detection and quantitation. A total of eight blots were used for each protein target detection. We loaded internal standards at an identical concentration in each blot to normalize the signal between the samples and the blots. For CD9 relative quantitation, we extracted EVs from a mixture of several unrelated samples as an internal control and added as a separate lane into each gel to enable gel-to-gel relative quantitation of the signal. For the relative quantitation of LRRK2, we used the same amount of the recombinant LRRK2 protein purchased from Thermo Fisher as an internal control for gel-to-gel relative quantitation of signal. Finally, for pSer1292-LRRK2 relative quantitation, we carried out in vitro autophosphorylation assay of the purchased recombinant LRRK2 protein, as described previously, and loaded the phosphorylated protein as an internal control for all phospho-LRRK2 Western blot detection experiments.

For the validation experiments, the membranes were cut according to the appropriate molecular weights to detect the target proteins at their corresponding molecular weights before blocking and incubated with the following primary antibodies: rabbit anti-CD9 (clone D3H4P; Cell Signaling Technology) at 1:5000 ratio, or rabbit anti-STK11 (clone D60C5; Cell Signaling Technology) at 1:1000 ratio, or mouse anti-PCSK1N (clone NP_037403.1; Millipore-Sigma) at 1:1000 ratio, together with rabbit anti-HNRNPA1 (clone D21H11; Cell Signaling Technology) at 1:1000 ratio. For the secondary antibody visualization, Goat anti-Rabbit or Goat anti-Mouse Alexa-Fluor 800 nm (Thermo Fisher Scientific) was used to bind the primary antibodies. An equal amount of pooled urine EVs was loaded in lane 1 of each gel to normalize the signal between two blots. The signal for each sample was then normalized to CD9.

### LC-MS sample preparation

Phase-transfer surfactant (PTS) aided procedure was used to lyse the dried EVs and extract proteins^40^. First, EVs were resuspended in the lysis solution containing 12 mM sodium deoxycholate (SDC; Sigma-Aldrich, cat. no. D6750), 12 mM sodium lauroyl sarcosinate (sarkosyl; Sigma-Aldrich, cat. no. L9150), 10 mM TCEP-HCl (Sigma-Aldrich, cat. no. C4706), 40 mM CAA (Sigma-Aldrich, cat. no. C0267), and phosphatase inhibitor cocktail (Millipore-Sigma, cat. no. P2850) in 50 mM Tris·HCl, pH 8.5 (Tris base; Fisher BioReagents, cat. no. BP152-1 and HCl; Fisher Chemical, cat. no. A144SI-212) by incubating 10 min at 95 °C. During this step, the proteins were also denatured, reduced, and alkylated. The samples were diluted fivefold with 50 mM triethylammonium bicarbonate and digested with Lys-C (Wako Chemicals, cat. no. 129-02541) at 1:100 (wt/wt) enzyme-to-protein ratio for 3 h at 37 °C. For further protein digestion, trypsin (proteomics grade, modified; Sigma-Aldrich, cat. no. T6567) was added to a final 1:50 (wt/wt) enzyme-to-protein ratio for overnight digestion at 37 °C. Then, the samples were acidified with trifluoroacetic acid (TFA; Merck, cat. no. 8082600100) to a final concentration of 1% TFA. An ethyl acetate (Fisher Chemical, cat. no. E145-4) solution was added at a 1:1 ratio to the samples. The mixture was vortexed for 2 min and then centrifuged at 20,000 × *g* for 2 min to obtain aqueous and organic phases. The organic phase (top layer) was removed, and the aqueous phase was collected, dried down to <10% original volume in a vacuum centrifuge, and desalted using TopTip C18 tips (Glygen Corporation, cat. no. TT2C18) according to the manufacturer’s instructions. After desalting, the peptide concentrations were determined by the Pierce Quantitative Peptide Colorimetry assay (Thermo Fisher, cat. No. 23275), and the samples were further normalized. Each sample was split into 99% and 1% aliquots for phosphoproteomic and proteomic experiments, respectively. The samples were dried entirely in a vacuum centrifuge and stored at −80 °C. For phosphoproteome analysis, 99% portion of each sample was subjected to phosphopeptide enrichment using a PolyMAC Phosphopeptide Enrichment kit (Tymora Analytical Operations, cat. no. 707) according to manufacturer’s instructions, and the eluted phosphopeptides dried completely in a vacuum centrifuge. The whole enriched sample was loaded onto LC-MS for phosphoproteomics analysis, while only 50% of each sample (equivalent to 0.5 µg) was injected for proteomics.

### LC-MS analysis

Both proteomic and phosphoproteomic samples were spiked with an 11-peptide indexed Retention Time internal standard mixture (Biognosys) to normalize the LC-MS signal between the samples. All samples were captured on a 2-cm Acclaim PepMap trap column (PN 164535, Thermo Fisher Scientific) and separated on a heated 50-cm Acclaim PepMap column (PN 164942, Thermo Fisher Scientific) containing C18 resin. The mobile phase buffer consisted of 0.1% formic acid (FA; Sigma-Aldrich, cat. no. F0507) in HPLC grade water (buffer A) with an eluting buffer containing 0.1% formic acid in 80% (vol/vol) acetonitrile (ACN; Fisher Scientific, cat. no. A955-4) (buffer B) run with a linear 60-min gradient of 6–30% buffer B at a flow rate of 300 nL/min. The UHPLC was coupled online with a Q-Exactive HF-X mass spectrometer (Thermo Fisher Scientific). The mass spectrometer was run in the data-dependent mode, in which a full-scan MS (from *m/z* 375 to 1500 with the resolution of 60,000 at *m/z* 200) was followed by MS/MS of the 15 most intense ions (30,000 resolution at *m/z* 200; normalized collision energy—28%; automatic gain control target (AGC)—2E4, maximum injection time—200 ms; 60 s exclusion].

### Parallel reaction monitoring (PRM)

Peptide samples were dissolved in 10.8 μL 0.05% TFA & 2% ACN and injected 10 μL into the UHPLC coupled with a Q-Exactive HF-X mass spectrometer (Thermo Fisher Scientific). The mobile phase buffer consisted of 0.1% formic acid in HPLC grade water (buffer A) with an eluting buffer containing 0.1% formic acid in 80% (vol/vol) acetonitrile (buffer B) run with a linear 60-min gradient of 5–35% buffer B at a flow rate of 300 nL/min. Each sample was analyzed under PRM with an isolation width of ±0.8 Th. In these PRM experiments, an MS2 level at 30,000 resolution relative to *m/z* 200 (AGC target 2E5, 200 ms maximum injection time) was run as triggered by a scheduled inclusion list. The inclusion list included peptides that have been manually picked and compared to PeptideAtlas^41,42^. Higher-energy collisional dissociation was used with 28 eV normalized collision energy. PRM data were manually curated within Skyline-daily (64-bit) 22.1.9.208 (6839020bd)^43^.

### LC-MS data processing

The raw files were searched directly against the human Swiss-Prot database with no redundant entries, using Byonic (Protein Metrics) and Sequest search engines loaded into Proteome Discoverer 2.3 software (Thermo Fisher Scientific). MS1 precursor mass tolerance was set at 10 ppm, and MS2 fragment tolerance was set at 20 ppm. In the processing workflow, search criteria for both search engines were performed with full trypsin/P digestion, a maximum of two missed cleavages allowed on the peptides analyzed from the sequence database, a static modification of carbamidomethylation on cysteines (+57.0214 Da), and variable modifications of oxidation (+15.9949 Da) on methionine residues and acetylation (+42.011 Da) at N terminus of proteins. Phosphorylation (+79.996 Da) on serine, threonine, or tyrosine residues was included as the variable modification for phosphoproteome analysis. The false-discovery rates of proteins and peptides were set at 0.01. All protein and peptide identifications were grouped, and any redundant entries were removed. Unique peptides and unique master proteins were reported. Finally, the proteomic results were further normalized against common urine EV proteins to account for any other variations in urine concentration.

### Label-free quantitation analysis

The label-free quantitation node of Precursor Ions Quantifier in the consensus workflow through the Proteome Discoverer v2.3 (Thermo Fisher Scientific) was used to quantify all data. For the quantification of proteomic and phosphoproteomic data, the intensities of peptides were extracted with initial precursor mass tolerance set at 10 ppm, fragment mass tolerance at 0.02 Da, minimum peak count as 1, maximum RT shift as 5 min, PSM confidence FDR of 0.01 as strict and 0.05 as relaxed, with hypothesis test of *t*-test (background based), protein abundance based ratio calculation, 100 as the maximum allowed fold-change, and site probability threshold of 75. The abundance levels of all peptides and proteins were normalized to the spiked-in internal iRT standard. For calculations of protein abundance, the sum of sample abundances of the connected peptide groups was added together and used for downstream analysis.

### Bioinformatics analysis

All clinical sample data were analyzed using the Perseus software (version 1.6.5.0)^44^. The normalized intensities of proteins and phosphoproteins were extracted from Proteome Discoverer search results and log-based 2 transformed for quantifying both proteomic and phosphoproteomic data. The abundances were categorized into four different categories: Control, NMC, iPD, and LRRK2 PD. The proteins or phosphoproteins with detected abundances of more than 70% in each category were kept. It was done to keep the proteins and phosphoproteins detected in at least one category. The imputation for the missing abundances was performed by assigning small random values from the normal distribution with a downshift of 1.8 SDs and a width of 0.3 SDs. Very low abundances normally cause missing values.

All abundances for each protein or phosphoprotein were further normalized by subtracting the median from each protein or phosphoprotein abundance. Then, the unpaired Student’s two-tailed *t*-test was performed, and the difference in averages was calculated for the three comparisons. Various packages in R 3.5.0^45^, including but not limited to ggplot2 3.3.1^46^, ggpubr 0.3.0^47^, EnhancedVolcano 1.7.6^48^, pROC^49^, Vennerable 3.0^50^, and Circlize 0.4.9^51^, and also Cytoscape 3.8.0^52^ (an open-source software platform for visualizing complex networks) were used to visualize the data. For the volcano plots, the *x*-axis is the log(2) fold-change on averages, and the *y*-axis is the log(10) of the *p*-value. Volcano plots were created for each comparison with cutoff values of permutation-based FDR = 0.05 (−log10(0.05) = 1.30) and log base 2 fold-change = 0.5, which equals ~1.414 fold-change. The Venn diagrams were created based on the upregulated proteins or phosphoproteins in the volcano plots. The violin plots were generated by focusing on significant proteins and phosphoproteins from the overlapped area in the Venn diagrams. The unpaired two-sample Wilcoxon test *p*-values and the one-way ANOVA *p*-value were included on each of the violin plots. The unpaired two-sample Wilcoxon test *p*-values were shown for the comparison between gender and specific proteins. The correlations between potential biomarker expressions with gender, age, disease duration, and MoCA were created with a minimal 0.6 for *R*^2^ and a maximal 0.05 for *p*-value calculated using t-distribution with n-2 degrees of freedom as thresholds. Lastly, STRING v11.5^53^ and IPA^54^ (QIAGEN Inc., https://www.qiagenbioinformatics.com/products/ingenuitypathway-analysis) were used to analyze the protein–protein interactions and validate their respective protein roles in hallmark PD pathways.

### Division Into training set and test set, feature selection, and predictive analyses

One hundred thirty-eight unique subjects were divided randomly into the discovery and validation experiments. In total, 82 subjects were categorized into the main experiment, further divided into training (57 subjects) and test sets (25 subjects). Fifty-nine subjects were used for the validation experiments, consisting of parallel reaction monitoring and Western blot experiments. To facilitate the top feature selection using machine learning, we created volcano plots for each comparison in training set with cutoff values of unpaired Student’s two-tailed *t*-test *p*-value = 0.05 (−log10(0.05) = 1.30) and log base 2 fold-change = 0.5, which equals to ~1.414 fold-change. For the discovery experiment, we first performed feature selection on the biomarker candidates. The goal was to discover disease biomarkers in an independent manner. Disease biomarkers are upregulated in iPD and LRRK2 PD compared to HC and NMC. For the feature selections, machine learning, and predictive analyses, we utilized various packages, such as python 3.8.8^55^, conda 4.13.0^56^, jupyter-notebook 6.3.0^57^, pandas 1.4.3^58^, numpy 1.20.1^59^, matplotlib 3.3.4^60^, plotly 5.6.0^61^, sklearn 1.1.1^62^, mlxtend 0.20.0^63^, and xgboost 1.6.1^64^. Instead of using a simple one-shot feature selection technique that usually yields a sub-optimal solution, we used a two-step feature selection process that generates better performance: backward feature elimination followed by exhaustive feature selection^65^. We deployed backward feature elimination, which removes, one feature at a time, those features that did not have a meaningful effect on the dependent variable or prediction of output. Then, we deployed exhaustive feature selection, which aims at finding the best-performing feature subset by searching across all possible feature combinations (a brute-force method) until the desired number of features is left. Specifically, this number was determined by observing the increase in performance (accuracy) with the increase in the number of final selected features (in which it is diminishing return). For the 48 potential disease biomarkers, we performed feature selection as follows: 48 features -> backward feature elimination -> 14 features -> exhaustive feature selection -> 6 features. At the end of the feature selection, we discovered a list of the top six disease biomarkers.

Next, we performed a hyperparameters selection process which included a randomized search followed by an exhaustive search on a random forest classifier with 10-fold cross-validation utilizing the top six disease biomarkers. In particular, we searched over the following set of hyperparameters: ‘n_estimators,’ ‘max_features,’ ‘max_depth,’ ‘min_samples_split,’ ‘min_samples_leaf,’ and ‘bootstrap’ in which we validated the result by using 10-fold cross-validation.

The best set of hyperparameters for disease biomarkers is as follows: ‘bootstrap’: True, ‘max_depth’: 4, ‘max_features’: ‘auto’, ‘min_samples_leaf’: 3, ‘min_samples_split’: 3, ‘n_estimators’: 320.

In the randomized search, we searched across 200 different combinations of hyperparameters and then created the hyperparameter grid encompassing the optimal sampled hyperparameter combination from the randomized search. An exhaustive search was used to select the best-performing set of hyperparameters from the generated grid. Finally, we repeatedly train a Random Forest Classifier with the selected features and selected set of hyperparameters as obtained from the above processes 50 times. After that, we evaluate each of the constructed models using accuracy, confusion matrix, and ROC curve. To summarize the results over all trials, we compute the mean and 95% confidence interval of each evaluation metric.

### Reporting summary

Further information on research design is available in the Nature Portfolio Reporting Summary linked to this article.

## Results

### Urine EV phosphoproteomics study design and sample quality control for PD biosignature development

For decades, scientists have been focusing on PD genotype marker discovery. In the case of *LRRK2-*G2019S mutation diagnosis alone, people are often found to either have the mutation in their genome without even experiencing PD (non-manifesting carrier/NMC) or do not have the mutation in their genome although they are suffering from PD (idiopathic PD/iPD). Furthermore, whether people with diagnosed NMC will develop PD later in their lives remains unclear. Multiple recent studies have shown that analysis of proteins and phosphoproteins, in many cases, provides a better snapshot of cellular processes and disease progression than genomic or transcriptomic investigations^66–69^. Proteome/phosphoproteome profiling efforts have already demonstrated substantial advantages for disease diagnosis and prediction of treatment response^70–73^. This is particularly true for kinase-dependent conditions and kinase inhibitor drugs^74–76^. Using this study design, we have further confirmed what is already known in the PD research community—that genotype markers are unreliable. Therefore, there is a critical need to shift the focus to developing protein- and phosphoprotein-based biomarkers for PD detection instead. Since the *LRRK2*-G2019S mutation alters the phosphorylation activity and these changes are reflected in extracellular vesicles, this supports the rationale behind using EVs as promising biosignature sources for PD diagnosis. Moreover, considering that many phosphorylation events directly reflect cellular physiological status, urinary EVs represent a highly promising source of proteins and phosphoproteins as non-invasive PD markers^23,24^. This is further reinforced by the recent studies showing Parkinson’s disease relevance of LRRK2 phosphorylation in urinary EVs^77–79^ and *LRRK2*-G2019S mutation influence on neat urine proteome^80^.

**Fig. 1:**
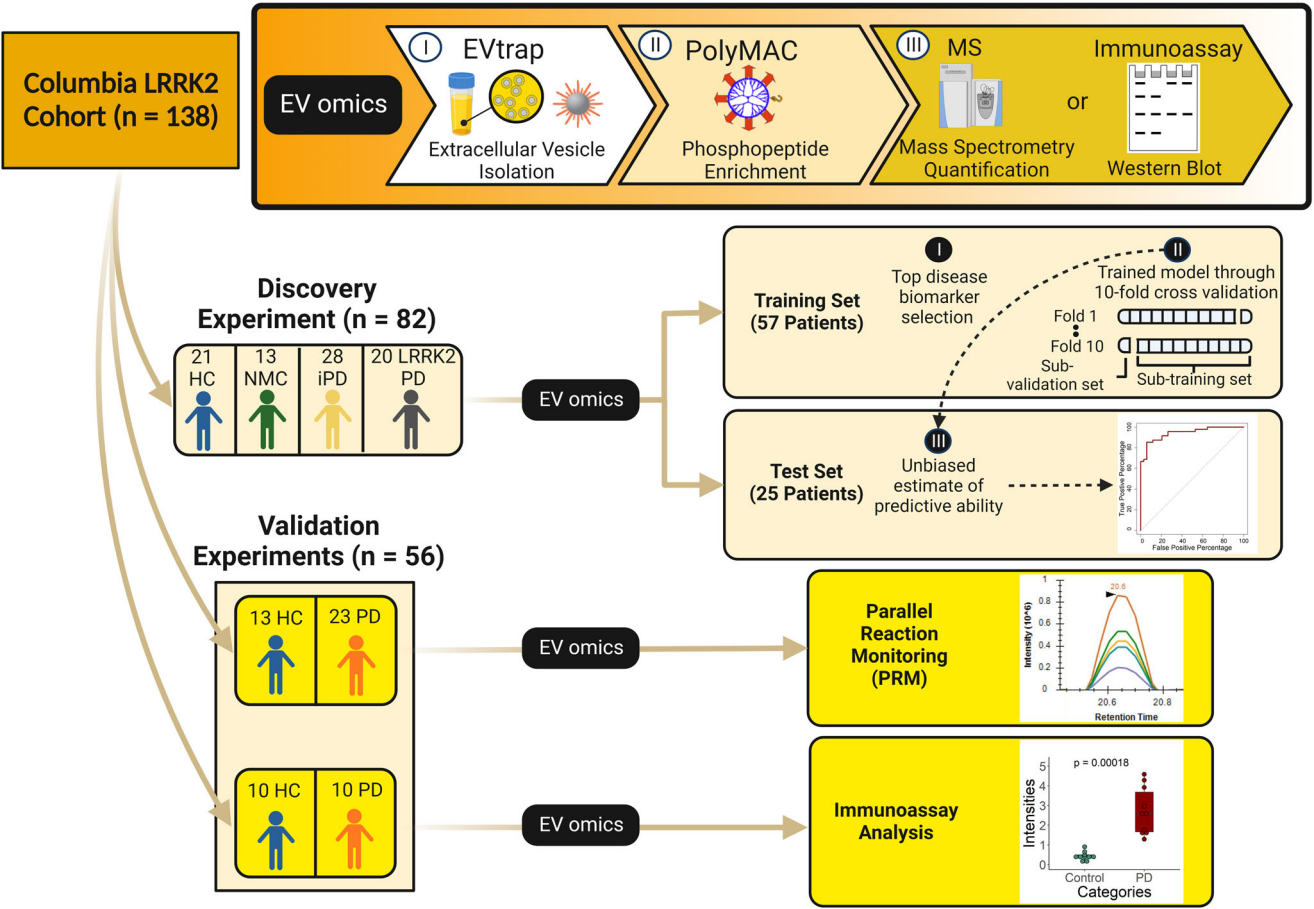
The development and validation of biomarker signatures for the diagnosis of Parkinson’s disease. A total of 138 urine samples were divided into two groups: the discovery and validation experiments. The urine samples were processed using our in-house (I) EVtrap for EV isolation and (II) PolyMAC (where applicable) for phosphopeptides enrichment. In the discovery experiment, the available 82 clinical urine samples were further randomly distributed into training and test sets for biomarker prediction. We proposed categorizing the potential biomarkers as the potential biomarkers for PD regardless of the *LRRK2*-G2019S mutation. Utilizing machine learning, we discovered the top disease biomarkers. Furthermore, we also trained our model using the 10-fold cross-validation and unbiasedly estimated the predictive ability of the test set. For biomarker validation, another 56 clinical urine samples were divided into two groups for parallel reaction monitoring (PRM) and immunoassay analysis. HC healthy control, NMC non-manifesting carrier, iPD idiopathic Parkinson’s Disease, LRRK2 PD LRRK2 Parkinson’s Disease, PD Parkinson’s Disease.

Urine samples were collected at Columbia University Irving Medical Center (CUIMC) from four cohorts with or without PD and with or without the common G2019S mutation in the *LRRK2* gene^32^. The samples were collected from March 2016 to April 2017 under a Michael J. Fox Foundation (MJFF)-funded LRRK2 biomarker project. The participants underwent clinical evaluation of their cognitive functions using the Montreal Cognitive Assessment (MoCA) and motor skills using the Unified Parkinson’s Disease Rating Scale part III (UPDRS-III). This sample cohort has been uniquely curated for in-depth analysis and comparison of *LRRK2* genotype and activity effects on PD as previously described^32,81^. These 138 samples were divided into three groups: the discovery experiment (82 samples) and two validation experiments (56 samples) (Fig. 1). We were fully blinded to the identity of all samples until after the complete analysis. These four groups—control, NMC, iPD, and LRRK2 PD—were the major components of this biosignature study design. The demographic information for all samples is provided in Table 1.

**Table 1:** The summary of cohort demographics and clinical characteristics for all 82 patients whose samples were used in the discovery experiment.

Demographics	Control	NMC	iPD	LRRK2 PD	Overall
Age (range; years)	69.4 (59–85)	58.5 (37–83)	66.1 (45–82)	69.7 (56–90)	66.6 (37–90)
Gender (Female/Male)	10/11	6/7	12/16	9/11	37/45
Disease duration (range; years)	N/a	N/a	0–18	1–26	0–26
MoCA	27.7 (24–30)	28.5 (27–30)	27.2 (23–30)	26.2 (8–30)	27.3 (8–30)
UPDRS-III	1.1 (0–5)	0.8 (0–3)	17.5 (5–38)	21.1 (1–53)	11.5 (0–53)

The range units for age and disease duration are in years (see Supplementary Data 11 for more details). The groups include healthy individuals (control), healthy individuals with G2019S mutation in the *LRRK2* gene (non-manifesting carrier/NMC), PD individuals without G2019S mutation (idiopathic PD/iPD), and PD individuals with G2019S mutation (LRRK2 PD).

To evaluate the quality of samples and demonstrate the superior efficiency of isolating urinary EVs by EVtrap, we first selected a few representative samples and analyzed them using Tunable Resistive Pulse Sensing (TRPS), Western blotting with anti-CD9 and anti-LRRK2 antibodies, and LC-MS analyses. Nanoparticle size and distribution analysis with qNano (TRPS) of EVtrap- and ultracentrifugation (UC)-enriched urine EV samples both demonstrated a similar range of the isolated EVs, with the majority being in the 100–200 nm range (Supplementary Fig. 1a, b). Here, EVtrap showed a higher concentration of isolated EVs, as demonstrated in a previous publication^37^. Similarly, the detection of CD9 and LRRK2 target proteins by Western blot from five randomly selected urine samples revealed a significant increase in signal levels for both proteins after EVtrap isolation compared to UC (Supplementary Fig. 1c). Finally, to show the repeatability of our analytical procedure (from EVtrap enrichment to LC-MS analysis), we split a random human urine sample into six aliquots and processed them separately for LC-MS analysis. The raw intensities were log-based 2 transformed, filtered (70% quantification), and imputed before the coefficient of variation (CV) calculation. Supplementary Fig. 2a demonstrates the outstanding repeatability of the procedure, with almost all of the proteins detected and quantified across all six samples falling under 10% CV) and the vast majority under 5% CV (complete proteomics data in Supplementary Data 1). Moreover, we identified 90 EV markers from the top 100 EV markers and common EV proteins as listed in ExoCarta, such as CD9, CD63, and CD81, which were included as minimal information for studies of extracellular vesicles 2018 (MISEV 2018)^82,83^. MISEV 2018 guidelines also proposed that it is more appropriate to show depletion than to expect a binary presence/absence of proposed urine negative markers in urinary EVs. To show the depletion of high-abundant free urine proteins after EVs isolation, the raw intensities for all quantified proteins in 12 samples (six pairs of direct urine and urinary EVs isolated using EVtrap, where each pair was from the same urine sample with the same volume) were log-based 2 transformed, filtered (six minimal valid values in at least one group), and imputed before the fold-change calculation. As expected, we showed in Supplementary Fig. 2b that some of the most abundant proteins in urine, such as ALB (Fold-change = −181), UMOD (Fold-change = −65), and AMBP (Fold-change = −596), were significantly depleted in urinary EVs (paired Student’s two-tailed *t*-test *p*-values were shown, see Supplementary Data 1 for complete data)^84^.

### Urinary EVs as prominent sources of potential PD biomarkers

We processed 82 urine samples individually for the discovery experiment following the illustrated workflow in Supplementary Fig. 2c using approximately 10–15 mL of each urine after normalization by creatinine concentration. As the first step, we employed EVtrap to capture the complete EV profile from the urine samples using the synthesized magnetic beads described previously^37^. After EV lysis and protein digestion, a small portion of each sample (~1%) was used for direct proteomic analysis, and the rest (~ 99%) was used for phosphoproteomic analysis. We carried out phosphopeptide enrichment using our in-house developed dendrimer-based PolyMAC method on the remaining majority of each sample and analyzed by LC-MS. Indexed Retention Time Standard containing 11 artificial synthetic peptides was added to all proteomic and phosphoproteomic samples for improved peptide quantitation and reproducibility.

**Fig. 2:**
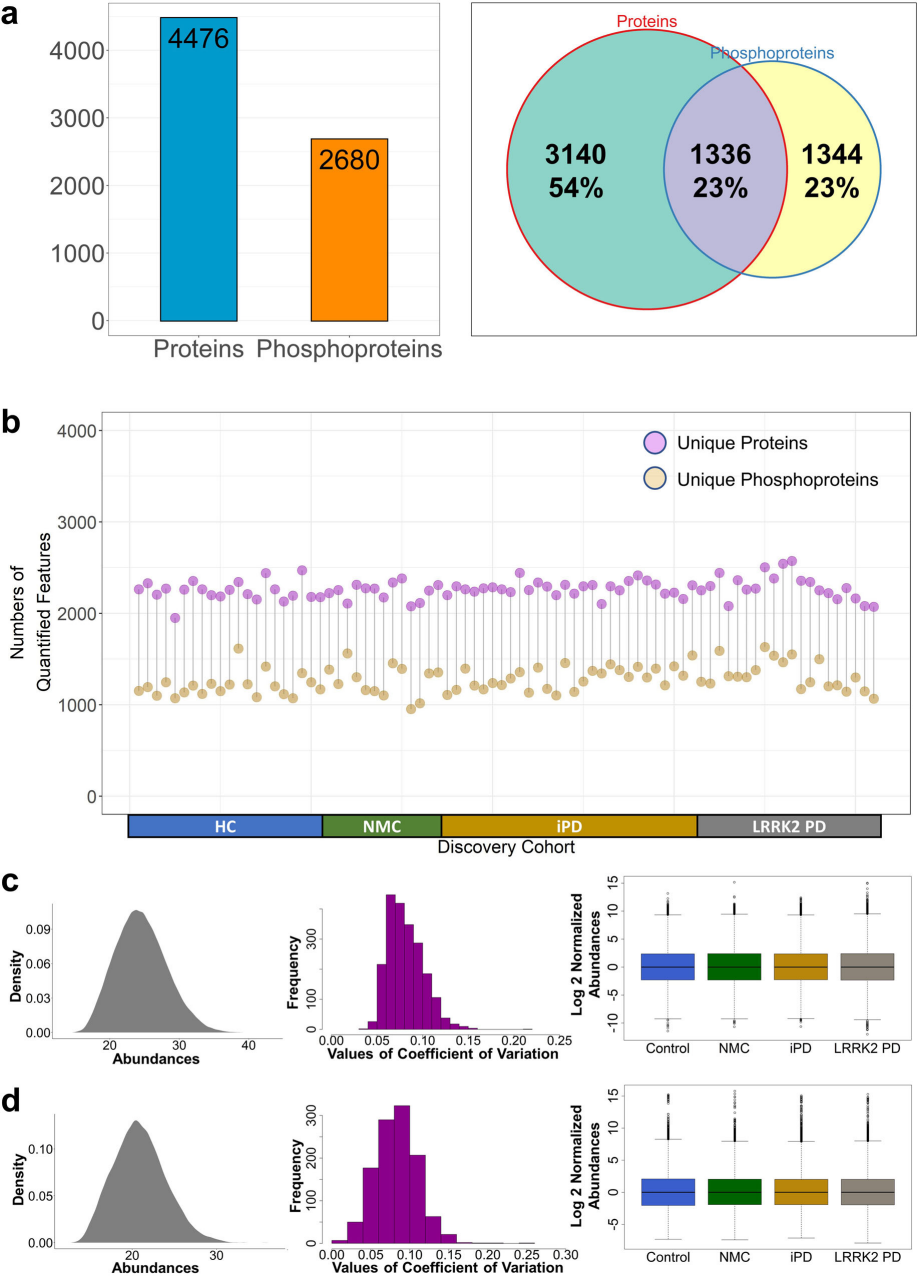
The summary of identification and quantification for all 82 patients. **a** The bar graphs and overlapped Venn diagram of unique identified proteins and phosphoproteins. **b** Cleveland Dot Plots for all quantified proteins and phosphoproteins. Both **c** proteomic and **d** phosphoproteomic data were normalized based on internal standards. Most quantified proteins and phosphoproteins had a CV of less than 20%. Normalized quantified data in the training set were then analyzed using feature selection to find potential biomarkers for PD. The box plots were derived from 21 HC, 13 NMC, 28 IPD, and 20 LRRK2 PD. For the lines in box plots: the line inside the box is the 50th percentile (median), the bottom and top of the box are the 25th and 75th percentiles, the whiskers are the 95% confidence interval, and any outliers are shown as open circles.

Our urinary EV proteomic and phosphoproteomic analyses identified and quantitated 4476 unique proteins from 46,240 peptide groups and 2680 unique phosphoproteins from 10,620 phosphopeptide groups (see Fig. 2a for identified features and Fig. 2b for quantified features, Supplementary Data 2 and 3). We evaluated whether our identified EV proteins and phosphoproteins were a good source for PD assessment. We compared our data with the brain-specific RNA-seq data downloaded from the Human Protein Atlas website^85^. We used 2587 proteins classified as brain-elevated from the Human Protein Atlas dataset to compare our EV protein and phosphoprotein data. We found that 8.9% of our EV proteins were denoted as brain-elevated (Supplementary Fig. 3). While the brain is likely a minimal source of EV proteins in urine^86^, this finding strengthens our hypothesis that urinary EV proteins and phosphoproteins are great candidates as potential biomarkers for PD.

We normalized both the proteome and phosphoproteome data based on internal standards. Figure 2c, d confirmed that the data had been effectively normalized with a coefficient of variation (CV) of less than 20%. Before we divided the discovery experiment data into the training and test sets, we performed gene ontology, clinical parameter correlation, and pathway analyses using the complete data (*n* = 82).

### Functional annotation identifies immune response, complement activation, and vesicle-mediated transport as the most prominent etiologies of PD in urine EVs

**Fig. 3:**
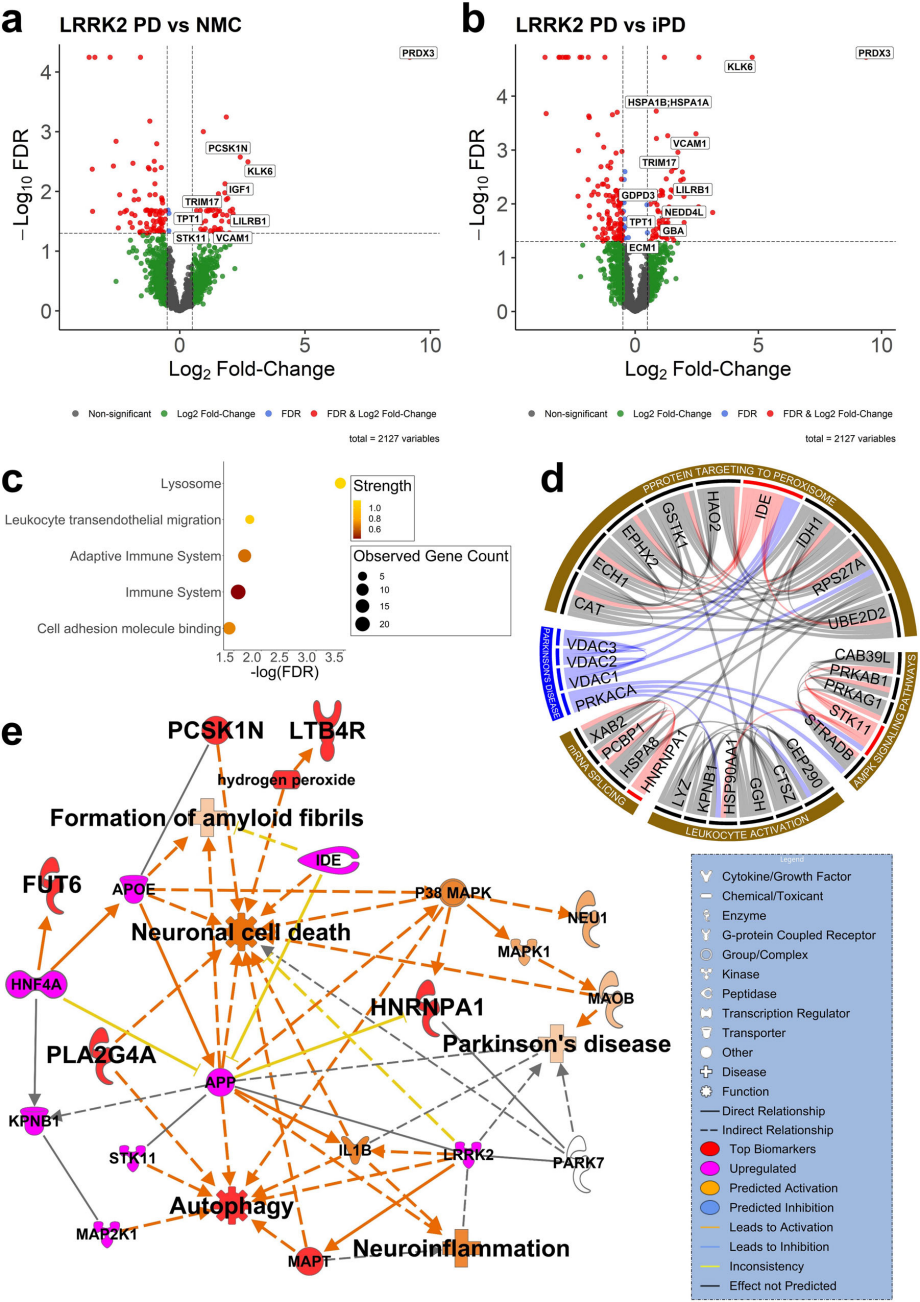
Protein and phosphoprotein biomarker network and pathway analysis. Volcano plots were created for **a** LRRK2 PD vs. NMC (20 and 13 individuals, respectively) and **b** LRRK2 PD vs. iPD (20 and 28 individuals, respectively) comparisons with cutoff values of permutation-based FDR = 0.05 and log base 2 fold-change = 0.5, which equals to ~1.414 fold-change. **c** GO analyses for LRRK2 PD compared to iPD samples. **d** A circos plot and **e** the IPA pathway analysis of the protein and phosphoprotein disease markers.

We identified the upregulated proteins and phosphoproteins in NMC, iPD, and LRRK2 PD groups against the controls along with LRRK2 PD vs. NMC and LRRK2 PD vs. iPD (permutation-based FDR < 0.05 and log base 2 fold-change > 0.5, which equals to ~1.414 fold-change, see Supplementary Data 4 for the complete proteomic data and Supplementary Data 5 for the complete phosphoproteomic data). The most interesting single feature among all upregulated proteins and phosphoproteins was PRDX3. PRDX3 protein, a hydrogen peroxide scavenger produced by mitochondria, was significantly overexpressed in LRRK2 PD vs. NMC (Fold-change = 580, *p*-value = 4.05E-14) and in LRRK2 PD vs. iPD (Fold-change = 665, *p*-value = 5.07E-22), respectively (see Fig. 3a, b, see Supplementary Data 4 for table format). Similar to PRDX3, KLK6, TRIM17, TPT1, VCAM1, and LILRB1 were also upregulated in both comparisons. Focusing on the upregulated proteins, we performed gene ontology analysis (Gene Ontology, KEGG, and Reactome Pathways) to understand the correlation between all upregulated proteins and PD. We utilized the STRING database (v11.5) for biological process gene ontology analysis^53^. The gene ontology analyses were set with a threshold FDR of 5% after Benjamini–Hochberg correction. Gene ontology results are shown in Fig. 3c and Supplementary Fig. 4 and listed in Supplementary Data 6.

The abnormal glycation and glycosylation seem to be more common than previously thought in PD and may underlie inflammation and mitochondria-induced oxidative stress in a feed-forward mechanism^87^. Furthermore, since PD patients’ CSF appears to have a specific metabolomic signature that reflects alterations in glycation or glycosylation, it was not surprising to discover that some biological process alterations involving glycosylation were also enriched in the patients’ urine EVs^87,88^. Interestingly, we discovered that the glycoside metabolic process was upregulated in NMC (Supplementary Fig. 4a). As seen in Supplementary Fig. 4b, cytolysis, in which various reducing agents, including dopamine, inhibit, was also enriched in iPD^89^. The fact that dopamine production is diminished in PD supports the observed increase in cytolysis. In addition, the immune response and the complement activation, which is a part of the innate immune system, are both enhanced in iPD. Complement activation, a major inflammatory mechanism in PD, on melanized neurons tends to increase in the PD substantia nigra^90^. When investigated using magnetic resonance imaging, the signal intensity of melanized neurons in the substantia nigra pars compacta in PD patients was greatly reduced, suggesting that the increase of complement activation contributes to PD development^91^. Cellular catabolic and carbohydrate derivative catabolic processes were upregulated in LRRK2 PD (Supplementary Fig. 4c). Cell adhesion molecule binding, lysosome, leukocyte transendothelial migration, and adaptive immune system were enhanced in LRRK2 PD (Fig. 3c).

### Disease-related EV protein and phosphoprotein biomarkers are prominently involved in the autophagy pathway

All of the following pathway analyses have been previously reported to be involved in PD onset and progression, but not by the direct experimental evidence of the pathogenic mechanism performed in this study. As potential disease markers, HNRNPA1, IDE, and STK11 proteins are shown to be involved in certain pathways that are important in PD progressions, such as protein targeting to peroxisome, AMPK signaling pathway, leukocyte activation, and mRNA splicing (Fig. 3d, see Supplementary Data 7 for protein–protein interactions). These markers also interact closely with PRKACA, VDAC1, VDAC2, and VDAC3, known to be PD-related^92^. Moreover, four top disease markers, PCSK1N, HNRNPA1, pPLA2G4A, and pLTB4R, are known to be involved in such important PD pathways as neuronal cell death, neuroinflammation, autophagy, and formation of amyloid fibrils (Fig. 3e; see Supplementary Data 8 for more details). From the Ingenuity Pathway Analysis (IPA), the upregulation of IDE leads to neuronal cell death activation, while the upregulation of STK11 indirectly leads to autophagy activation. PLA2G4A and LTB4R phosphoproteins were shown to be involved in downstream GPCRs and MAPK signaling pathways (Supplementary Fig. 5a; see Supplementary Data 9 for table format). Meanwhile, the presence of NEU1, a lysosomal enzyme and a disease marker, supports the emerging concept that PD is a lysosomal disorder^93^ (Supplementary Fig. 5b; see Supplementary Data 9 for table format). Furthermore, the overexpression of PLA2G4A, LTB4R, and NEU phosphoproteins can trigger the autophagy pathway, one of the hallmark pathways in PD (Supplementary Fig. 5c; see Supplementary Data 10 for more details). Interestingly, NEU1 showed two contradicting downstream effects. The overexpression of low-density lipoprotein (LDL)-cholesterol by NEU1 inhibited autophagy. On the other hand, the inhibited expression of high-density lipoprotein (HDL)-cholesterol by NEU1 triggered autophagy. In Supplementary Fig. 5c, PLA2G4A is shown to indirectly activate autophagy, supporting the fact that PLA2G4A activation leads to the impairment of autophagy flux by directly increasing lysosomal membrane permeabilization (LMP)^94^. The interactions of LTB4R/RAC1/PAK1/p38 MAPK are also known to activate autophagy.

### Correlation of proteome and phosphoproteome profiles with clinical parameters

We investigated any correlations between the expression of the proteins and phosphoproteins with age, gender, disease duration, MoCA score, and UPDRS-III score of the patients (see Supplementary Data 11 for the cohort demographics and clinical characteristics). There is increasing evidence that sex is an important factor in the development of PD^95^. In men, the risk of developing PD is nearly twice as high as in women. However, women have a higher mortality rate and faster disease progression^96^. MoCA was initially designed to evaluate mild cognitive impairment associated with AD to assess memory, executive functions, and verbal fluency, among others, and can be applied in a short period of time^97^. The test has been used for the cognitive evaluation of patients with PD to identify cognitive deficits. MoCA scores range between 0 and 30, where a score of 26 or over is considered normal. UPDRS-III scoring method evaluates the patient’s motor skills ranging from 0 to 108, with 108 being the worst.

We found that the expression levels of ENPEP, GDPD3, NAGA, NEDD4L, QPRT, and SCAMP3 proteins in urine EVs were significantly higher (*p*-value < 0.05, calculated using the unpaired two-sample Wilcoxon test) in males than in females (Supplementary Fig. 6a). There were positive correlations in the expression of FUT6 (*R*^2^ = 0.83, *p* < 0.05) and HAO2 (*R*^2^ = 0.90, *p* < 0.005) proteins with age in the female NMC group, as seen in Supplementary Fig. 6b. Meanwhile, the expression of ALPL protein was negatively correlated with disease duration in the female iPD group (Supplementary Fig. 6c). Related to the MoCA scores in the male NMC group, we found a positive correlation in CAPN5 and HNRNPA1 proteins, and a negative correlation in ENPEP, GDPD3, and GPD1L proteins (Supplementary Fig. 6d). Additional significant correlations between protein abundance levels, MoCA scores, and gender are shown in Supplementary Fig. 6d.

At the phosphoprotein level, pNEU1 abundance was positively correlated with age in the female NMC group (*R*^2^ = 0.86, *p* < 0.01) (Supplementary Fig. 7a). DTD1 phosphorylation was positively correlated with MoCA in the female NMC group (Supplementary Fig. 7b). pANXA11 and pHLA-B were negatively correlated with MoCA in the male NMC group, while there were positive correlations for CYSRT1, LTB4R, and TJP3 phosphoproteins. In addition, MoCA in female NMC was negatively correlated with the expression of CYSRT1 phosphoprotein. Lastly, the pLTBR4 level in the male LRRK2 PD group was positively correlated with MoCA.

**Fig. 4:**
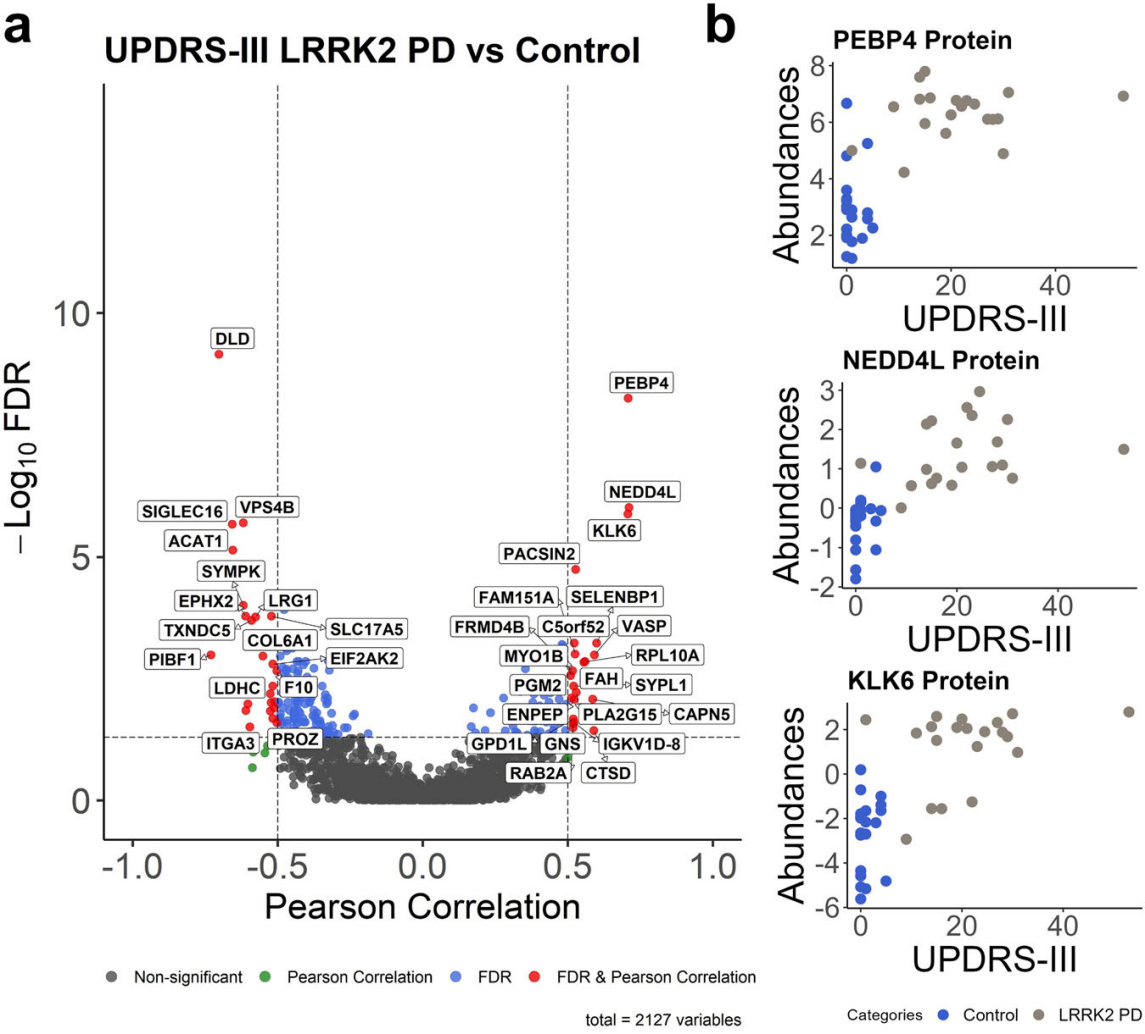
Correlations with clinical parameter, UPDRS-III. **a** Pearson correlation scores and associated FDR-values [−log_10_] of all protein intensities with the UPDRS-III scores; 20 LRRK2 PD and 21 HC were included. Significantly correlated proteins with an FDR of 5% after Benjamini–Hochberg correction are labeled. **b** The scatterplots of three biomarkers with strong Pearson correlation scores (>0.7).

Furthermore, we assessed the correlations of UPDRS-III scores with the protein and phosphoprotein intensities in iPD and LRRK2 PD patients versus those in healthy individuals. We found several proteins and phosphoproteins depicted in Fig. 4a and Supplementary Fig. 8 to be positively and negatively correlated with UPDRS-III and many that are moderately correlated with the UPDRS-III (0.5 < Pearson correlation < 0.7). Significantly correlated proteins with an FDR of 5% after Benjamini–Hochberg correction and Pearson correlation of more than 0.5 are labeled in the volcano plots. Lastly, Fig. 4b shows three proteins, PEBP4, NEDD4L, and KLK6, with higher than 0.7 Pearson correlation scores, denoting a strong correlation with UPDRS-III. These correlation data need to be further validated, and their relevance to PD evaluated in a translational manner.

### Top disease biomarkers were selected and evaluated using 10-fold cross-validation

The discovery experiment, which included samples from 21 healthy individuals (control), 13 healthy individuals with G2019S mutation in the *LRRK2* gene (non-manifesting carrier/NMC), 28 PD individuals without G2019S mutation (idiopathic PD/iPD), and 20 PD individuals with G2019S mutation (LRRK2 PD), were randomly divided into two groups: 70% training set and 30% test set for biomarker selection and predictive ability estimation (Fig. 1, Table 2 for HC, NMC, iPD, and LRRK2 PD patient distributions). The median normalization was performed on the training set so that all abundances in the four groups had the same median. After passing thresholds and robust normalizations, we obtained and quantified a total of 2127 qualified unique proteins and 1153 qualified unique phosphoproteins.

**Table 2:** The discovery experiment cohort distribution for machine learning.

	Training set	Test set
HC	15	6
NMC	9	4
iPD	19	9
LRRK2 PD	14	6

The number of HC, NMC, iPD, and LRRK2 PD patients after being randomly divided into the training and test sets.

From these curated training data, we generated six volcano plots for comparisons between NMC, iPD, and LRRK2 PD groups against the control samples with cutoff values of unpaired Student’s two-tailed *t*-test *p*-value = 0.05 (−log10(0.05) = 1.30) and log base 2 fold-change = 0.5, which equals to ~1.414 fold-change (Supplementary Fig. 9a, b; see Supplementary Data 12 for the proteome results, Supplementary Data 13 for the phosphoproteome results, and Supplementary Data 14 for overlapping proteins and phosphoproteins). Here, the volcano plots were created to facilitate the top feature selections using machine learning rather than finding significant features; therefore, multiple hypothesis corrections were not used as the cutoff. We need to emphasize that machine learning did not differentiate top biomarkers based on whether they were statistically significant; rather, machine learning made predictions by finding patterns within the data. The upregulated proteins and phosphoproteins overlapped in Venn diagrams. As mentioned previously, we investigated potential biomarkers identified as disease markers. We denoted disease markers as upregulated in PD regardless of the *LRRK2*-G2019S mutation (both iPD and LRRK2 PD groups). The upregulation of the disease biomarkers could indicate that a patient currently has PD. A single protein biomarker might be involved in several already-known diseases. To offer a better diagnostic value, we proposed to quantify a set of several biomarkers rather than a single diagnostic protein.

**Fig. 5:**
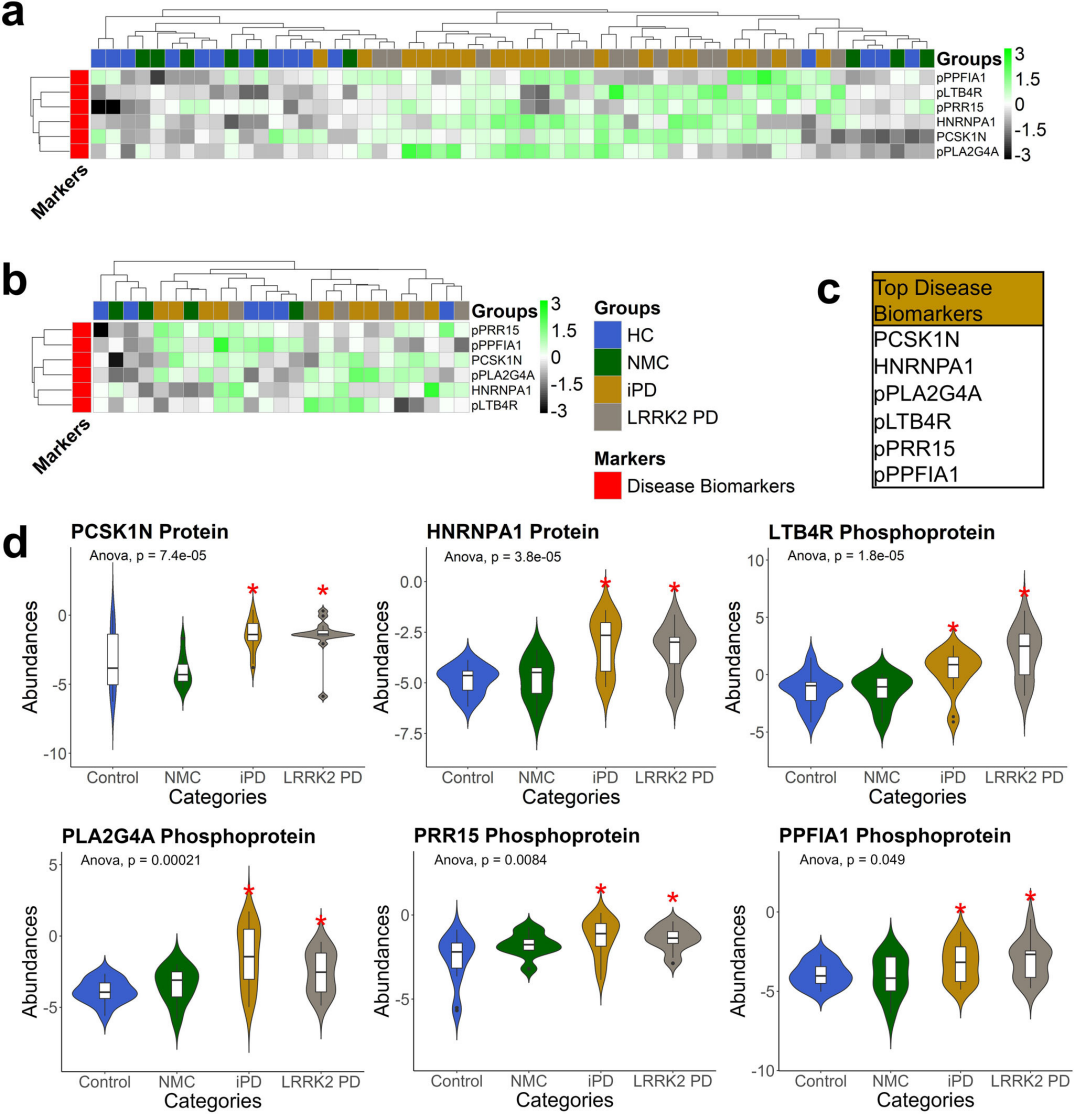
The selected top disease biomarkers acquired from the training set. **a** The training set’s heatmap of top potential protein and phosphoprotein biomarkers (Training set: 15 HC, 9 NMC, 19 iPD, and 14 LRRK2 PD). **b** The heatmap of top potential protein and phosphoprotein biomarkers on the test set (Test set: 6 HC, 4 NMC, 9 iPD, and 6 LRRK2 PD). **c** The table summary of the top disease biomarkers. **d** Violin plots of the statistically upregulated proteins and phosphoproteins from the training set in PD regardless of the *LRRK2*-G2019S mutation (disease markers). The heatmap was clustered by Euclidean distance and average method. The unpaired two-sample Wilcoxon test *p*-values and the one-way ANOVA *p*-value were calculated on each of the violin plots. The red stars above the violin plots indicate a statistically significant difference in the mean compared to those without the red star (*p*-value < 0.05). For the lines in box plots inside the violin plots: the line inside the box is the 50th percentile (median), the bottom and top of the box are the 25th and 75th percentiles, and the whiskers are the 95% confidence interval.

We first performed feature selection to select the top disease biomarkers. Instead of using a simple one-shot feature selection technique that usually yields a sub-optimal solution, we used a two-step feature selection process that generates better performance: backward feature elimination followed by exhaustive feature selection (See Table 3 for the list of feature selection inputs and the intermediate results after backward feature elimination and before exhaustive feature selection, see Supplementary Data 15 for more details of feature selection inputs)^65^. By utilizing this two-layer method, we could identify the top six disease biomarkers. Disease biomarkers are upregulated in iPD and LRRK2 PD compared to HC and NMC. The final selected disease biomarkers are shown in Fig. 5a, b and listed in Fig. 5c. Here, as expected, the iPD and LRRK2 PD groups clustered together, as well as the HC and NMC groups. The violin plots of the selected disease biomarkers are shown in Fig. 5d. Henceforth, we would label those biomarkers listed in Fig. 5c as top biomarkers; meanwhile, we would designate those biomarkers listed in Table 3 but not listed in Fig. 5c as potential biomarkers. We optimized our hyperparameters and trained our model using the random forest classifier with 10-fold cross-validation by utilizing the top six disease biomarkers. Lastly, we trained our model by utilizing the 10-fold cross-validation.

**Table 3:** Input disease biomarkers for machine learning.

Input disease biomarkers							
ABCA1	ERBB2	***➤ HNRNPA1***	***➤ PCSK1N***	STK11	pCLIC6	***➤ pLTB4R***	***➤ pPRR15***
ACAT2	FABP3	IDE	***PGM2***	TGM3	***pDKC1***	pNEU1	***pSPPL2B***
C4BPA	FAM151A	IGF1	RALA	UCHL1	***pDYNC1LI1***	***➤ pPLA2G4A***	pSSB
C6orf211;ARMT1	FLOT1	ITCH	RPS4X	UGP2	pEDN1	***➤ pPPFIA1***	pTBC1D9B
CAPN5	FUT6	***KLK10***	SAA4	VASP	pFNBP1	pPRG4	***pTJP3***
CC2D1A	GALNT7	NCCRP1	SLC22A13	pCLDN14	***pLAD1***	pPRKAR2A	***pTMPO***

List of the feature selection inputs (potential biomarkers) for disease biomarkers. Intermediate results after backward feature elimination and before exhaustive feature selection are bolded and italicized. The top biomarkers are marked with ***➤***.

### Disease biomarkers were substantiated using classification models, PRM-MS targeted mass spectrometry, and Western blot experiments

**Fig. 6:**
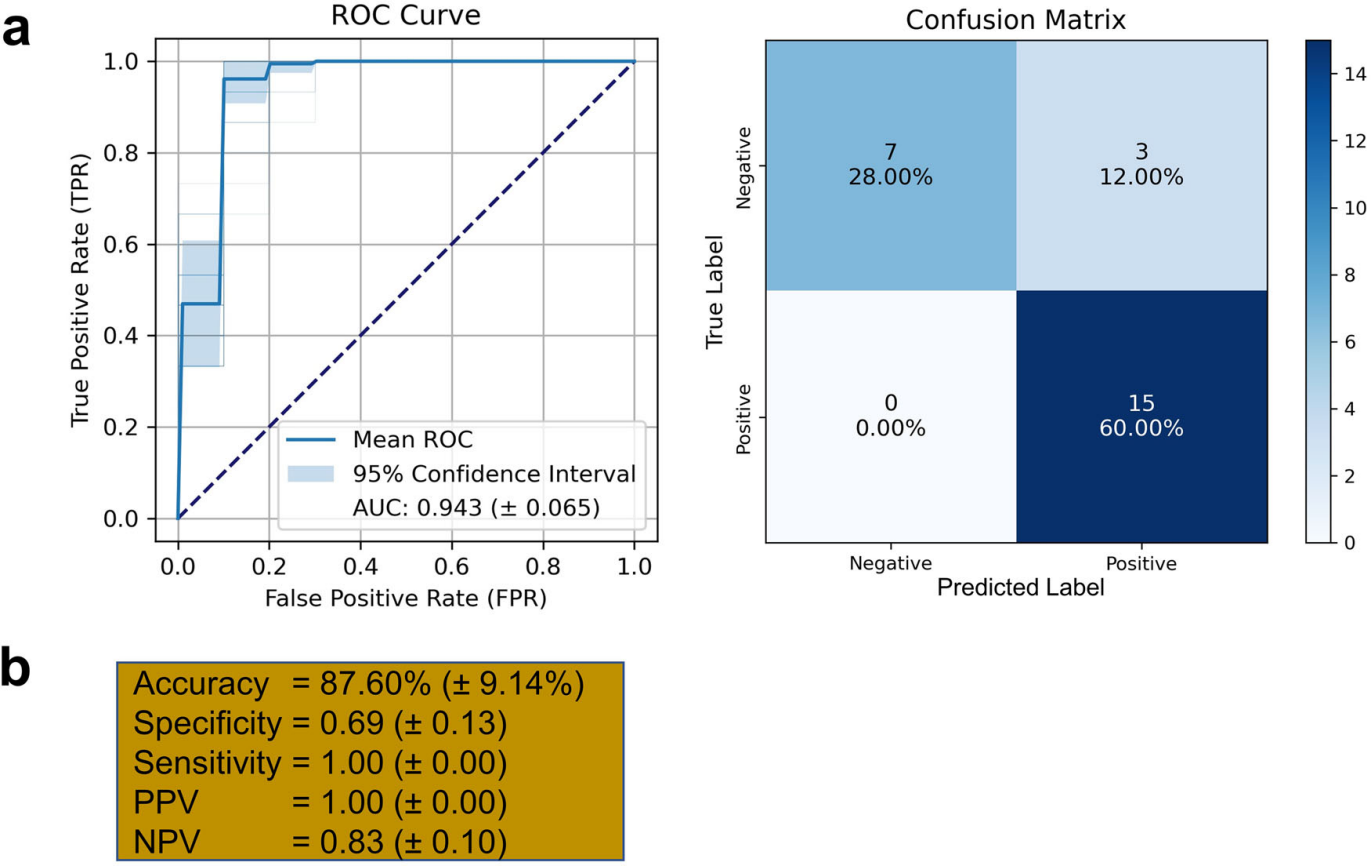
Unbiased estimation of predictive ability of urinary proteomes and phosphoproteomes on the test set. Receiver operating characteristic (ROC) curve and the confusion matrixes for the Random Forest Classifier model to classify 15 positive (Parkinson’s Disease) vs. 10 negative (Normal) individuals (**a**). In the ROC curve, the dotted diagonal line indicates random performance, and the light blue area represents the 95% confidence interval. The accuracy, sensitivity, specificity, positive predictive value (PPV), and negative predictive value (NPV) scores with their 95% confidence intervals are shown for PD vs. Normal (**b**).

After the careful feature selection and hyperparameters as described above, we tested our constructed model using accuracy scores, confusion matrixes, and receiver operating characteristic (ROC) curves, as depicted in Fig. 6. A confusion matrix evaluates one classifier with a fixed threshold, while the ROC evaluates that classifier over all possible thresholds. The area under the ROC curve (AUC) provides the performance measurement across the classification threshold. A higher true-positive percentage and a lower false-positive percentage will produce better AUC results. Normally, in the medical field, an AUC of 70–80% is considered acceptable, 80–90% is considered good, and 90–100% is considered excellent^98^. For example, the AUC for the top six disease biomarkers is 94.3%, with 87.60% confusion matrix accuracy (Fig. 6a, b). This panel would result in a 94.3% likelihood that the doctor will correctly distinguish a PD patient from a healthy patient based on finding the six biomarkers at an elevated level in the urinary EVs. Certainly, these findings need to be verified with a much more expanded validation cohort.

Parallel Reaction Monitoring-Mass Spectrometry (PRM-MS) and Western blot are both commonly used for the initial validation of potential biomarkers. We selected several of our top disease biomarkers and urinary exosome markers for further validation. Quantitative assays based on PRM-MS for the disease markers were performed with a new set of urine EV samples from 23 patients with PD and 13 healthy individuals (see Supplementary Data 11 for the cohort demographics and clinical characteristics). All of the samples used in the validation experiments came from a new cohort of patients. In this PRM-MS experiment, we targeted several top EV markers, proteins involved in PD pathways, proteins known to be PD biomarkers, several potential disease biomarkers (those biomarkers which were not chosen as top biomarkers by a feature selection), and finally, the top disease biomarkers (see Supplementary Table 1 for the target list, see Supplementary Data 16 for the inclusion list). Due to limited volumes of the new urine samples, we could only target protein biomarkers.

**Fig. 7:**
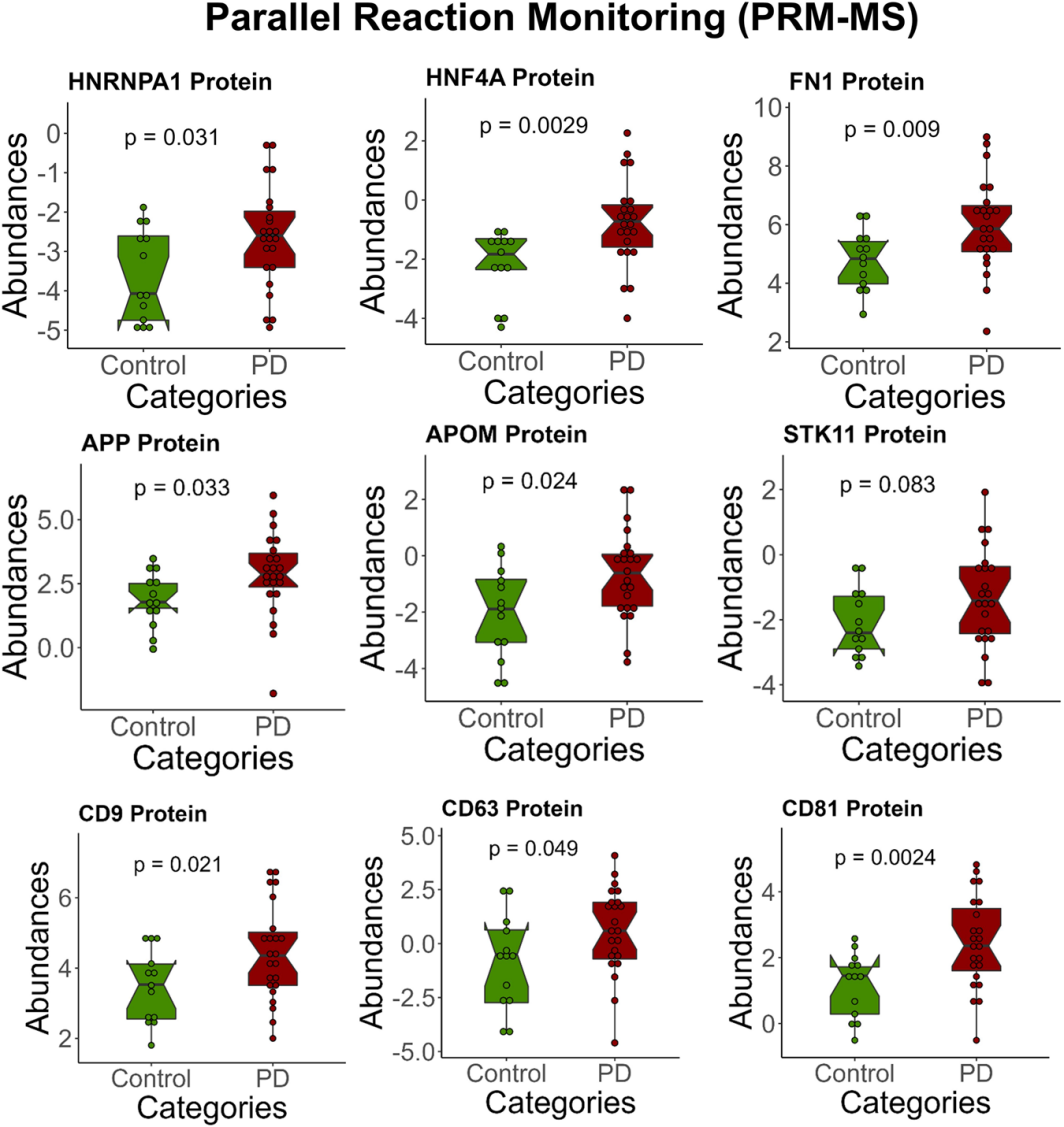
Targeted quantitation of disease biomarkers. A top disease biomarker, HNRNPA1, was validated in 23 patients with PD and 13 healthy individuals using PRM-MS (*p*-value < 0.05). HNF4A and FN1 were also significantly upregulated in PD (*p*-value < 0.01). The Student’s two-tailed *t*-test calculated all *p*-values. For the lines in box plots: the line inside the box is the 50th percentile (median), the bottom and top of the box are the 25th and 75th percentiles, and the whiskers are the 95% confidence interval.

One out of two targeted top disease biomarkers, HNRNPA1, was observed to be significantly upregulated in patients with PD compared to healthy individuals (Fig. 7, see Supplementary Data 16 for the table format). HNF4A, whose mRNAs were found to be upregulated in the blood of 51 PD patients vs. 45 controls using quantitative PCR assays, was significantly upregulated in PD (*p* < 0.01). Meanwhile, APP, whose mRNAs have been shown as blood biomarkers of PD, was also significantly upregulated in PD (*p* < 0.05, Fig. 7)^99^. Interestingly, the upregulation of CD9, CD63, and CD81 agreed with the previous finding that these three EV markers’ median fluorescence intensity (MFI) on the surface of plasma-derived EVs was significantly higher in PD compared to HC (*p* < 0.05)^100^. FN1, one of the proteins normally found to be co-purified with EVs, was also upregulated in PD patients (*p* < 0.01).

We further performed an immunoassay with this new cohort of urine EV samples from 10 patients with PD and 10 healthy individuals. Three disease markers, HNRNPA1, PCSK1N, and STK11, were noticeably upregulated in patients with PD compared to healthy individuals (Supplementary Fig. 10a, b, and see Supplementary Data 16 for the table format).

### LRRK2 and its Rab substrates signaling are altered but not significant PD biomarkers

Lastly, we explored whether some Rab phosphoproteins, which are known to be direct substrates of LRRK2 and identified in EVs, would be altered across different groups in the Columbia LRRK2 cohort. Therefore, we also investigated the direct LRRK2 activation in these urine EV samples in addition to new biomarker discovery. LRRK2 is known to phosphorylate a subgroup of Rab proteins, and *LRRK2*-G2019S mutation has been previously shown to increase the phosphorylation of its Rab substrates^16^. Rab proteins are master regulators of membrane trafficking, orchestrating vesicle formation and vesicle movement along actin and tubulin networks, as well as membrane docking and fusion—all critical aspects of autophagy and lysosome biology^16^. First, we performed Western blot analyses of all 82 urine samples, quantifying CD9 (common exosome marker), LRRK2, and pSer1292-LRRK2 signal in the EVs captured by EVtrap (Supplementary Fig. 11a, see Supplementary Data 17 for table format). pSer1292, an LRRK2 autophosphorylation site, indirectly reflects LRRK2 activation^101^. We normalized and compared the signal between all samples using a recombinant autophosphorylated LRRK2 protein as an internal standard^77^. As expected, the normalized CD9 signal did not show a significant difference between the sample groups (Supplementary Fig. 11b), while the expression of LRRK2 in LRRK2 PD was significantly higher than in the control samples (*p* = 0.028). Unfortunately, it was challenging to detect the pSer1292-LRRK2 signal in most samples, caused by a meager amount of this modified protein in the samples and/or a lower antibody sensitivity. Due to undetectable signals in most samples, we did not find any significant difference in the pSer1292-LRRK2 phosphorylation level itself (Supplementary Fig. 11b). We also compared the Western blot quantitative result with the mass spectrometry data (Supplementary Fig. 11c). While not all of the differences observed in these Western blot and mass spectrometry experiments showed statistically significant changes, there was an apparent trend of higher LRRK2 signal in the G2019S groups (NMC and LRRK2 PD) in both the Western blot and mass spectrometry data. Interestingly, the LRRK2 overall phosphorylation level (sites other than Ser1292) is lower in NMC and significantly lower in LRRK2 PD than in the control group. Indeed, the low level of phosphorylated LRRK2 in EVs might explain why it was challenging to detect pSer1292-LRRK2 signals in the Western blot.

From the urine EV LC-MS analyses, we identified 34 Rab GTPases, 12 of which are known LRRK2 substrates, and eight phosphorylated Rab GTPases (Supplementary Table 2). After in-depth statistical normalization and qualification, we quantified 15 Rab GTPases (10 LRRK2 substrates) and four phosphorylated Rab GTPases. We observed that Rab2A (*p* < 0.003) and Rab10 (*p* = 0.037) were significantly upregulated in LRRK2 PD compared to the control samples (Supplementary Fig. 12a). Rab2A’s involvement in retrograde trafficking and particle recycling from Golgi back to the endoplasmic reticulum (ER) shows the role of this protein in the organellar homeostasis pathway to prevent misfolded proteins from entering the Golgi apparatus^6^. Thus, the upregulation of Rab2A in LRRK2 PD, which may promote retrograde trafficking machinery, may be the α-synuclein aggregation stress response.

Rab10 is a well-known substrate of LRRK2, and in vitro assays suggested that PD-related neurodegeneration may start by *LRRK2*-G2019S increasing phosphorylation of Rab10^102^. It is also known that Rab10 is involved in LRRK2 and other Rabs relocalization^103^. Therefore, it is not surprising that Rab10 was present at higher levels in the LRRK2 PD group EVs (Supplementary Fig. 12a). Interestingly, Rab17 protein was qualified to be one of our progression markers, although currently, the role of Rab17 in PD progression is not fully understood. At the phosphoprotein level, only Rab12 (*p* < 0.005) was significantly upregulated in LRRK2 PD (Supplementary Fig. 12a). Rab12 is an LRRK2 endogenous substrate that plays a role in endosomal lysosome sorting, degradation, and autophagy^103^.

We also investigated the correlation of the identified Rab GTPase expression levels with age, gender, disease duration, and MoCA scores with the new biomarkers. As seen in Supplementary Fig. 12b, the expression of Rab1A protein in females was significantly higher than in males with *p* < 1e-12. In contrast, Rab1B (*p* < 0.0005), Rab3D (*p* < 0.005), and Rab7A (*p* < 0.05) were expressed at higher levels in males than females. We also observed that the expression level of Rab2A protein in female iPD individuals was positively correlated with age (*R*^2^ = 0.68, *p* < 0.001) (Supplementary Fig. 12c). Meanwhile, the expression of Rab17 protein in female iPD individuals was positively correlated with MoCA (*R*^2^ = 0.63, *p* < 0.005) (Supplementary Fig. 12d). As noted before, these correlations need to be reproduced and evaluated further to better understand their significance.

## Discussion

Mass spectrometry (MS)-based biofluid proteome analysis and quantitation have recently gained renewed interest and excitement in disease profiling efforts. The approach offers immeasurable potential for innovative biomarker discovery. However, successful translation from MS data to human disease profiling remains limited. This limitation is partly due to the complexity of biofluids, which have a very large dynamic range and are typically dominated by a few highly abundant proteins. To date, scientists have been concentrating on finding PD biomarkers in EVs of biofluids such as CSF and plasma without paying much attention to the importance of urinary EVs as a potential source of biomarkers^104^. Here, we report in-depth analyses of proteome and phosphoproteome in urinary EVs and demonstrate the viability of developing proteins and phosphoproteins as potential disease biomarkers. We present an MS-based strategy that includes isolating EV particles from human urine utilizing EVtrap, enrichment of EV phosphopeptides, in-depth LC/MS analysis, and robust bioinformatics evaluation for biomarker discovery and qualitative verification (Fig. 1). After we showed that our EV isolation method was reproducible and successfully depleted high-abundant free urine proteins, we analyzed EV samples from patients with *LRRK2*-G2019S mutation (NMC), idiopathic PD (iPD), and LRRK2 PD compared to healthy individuals to identify candidate disease biomarkers. In total, we identified and quantified 4476 unique proteins from 46,240 peptide groups and 2680 unique phosphoproteins from 10,620 phosphopeptide groups (Fig. 2a, b). Then, the proteins and phosphoproteins identified were normalized and analyzed to be statistically useful for further downstream analyses (Fig. 2c, d).

To show the robustness of our EV isolation method and downstream analyses, we identified PD-relevant features supported by the literature. Among the upregulated proteins, PRDX3 was significantly overexpressed in LRRK2 PD vs. NMC and LRRK2 PD vs. iPD comparisons (Fig. 3a, b). *LRRK2*-G2019S mutation has been shown to increase the phosphorylation of PRDX3, causing inhibition of endogenous peroxidases and increasing neuronal cell death^105^; however, interestingly, we did not detect the presence of PRDX3 phosphoprotein in urinary EVs. Similarly, KLK6, TRIM17, TPT1, VCAM1, and LILRB1 were also significantly upregulated in both comparisons, with KLK6 and LILRB1 also upregulated in LRRK2 PD vs. Control. In humans, the KLK6 protein is expressed at high levels in the nervous system and is one of the few most abundant serine proteases in the CSF, where it is secreted at mg/L levels^106^. Interestingly, KLK6 degrades α-synuclein and prevents its polymerization, suggesting that the nervous system secreted KLK6 through EVs to slow down α-synuclein degradation, and as a result, PD progression continues. Overexpression of TRIM17 increases α-synuclein expression^107^. Moreover, TPT1 mRNA is highly expressed in human substantia nigra with PD^108^. A significantly elevated expression of VCAM1 was observed in the blood of PD patients suggesting the role of VCAM1 in neuroinflammation and PD progression^109^. Recently, LILRB1 was discovered as a potential diagnostic signature for PD^110^.

For LRRK2 PD vs. NMC, IGF1, PCSK1N, and STK11 were significantly upregulated (Fig. 3a). The upregulation of IGF1 in our data matches the discovery that IGF1 was significantly increased in PD patients compared to HC in serum^111^. Meanwhile, STK11 upregulation indirectly leads to autophagy activation (Fig. 3e). These three proteins might give important insights into possible disease progression, especially when considering the possible confounding factor by age (Table 1). For LRRK2 PD vs. iPD, HSPA1A, HSPA1B, ECM1, GBA, NEDD4L, and GDPD3 were upregulated (Fig. 3b). Through microarray analysis on substantia nigra tissue, heat shock protein HSPA1A and HSPA1B were found to be upregulated in PD, indicating that this may be a common response to attenuate the adverse effects of misfolded protein^112^. ECM1 was also shown to be upregulated in the CSF samples of PD patients^113^. Interestingly, one of the most common genetic risk factors for PD is having a mutated GBA gene^114^.

In addition, it has been hypothesized that aging-related metabolic changes could contribute to the progression and onset of PD^115^. Therefore, it is not surprising to see that cellular catabolic and carbohydrate derivative catabolic processes were upregulated in LRRK2 PD (Supplementary Fig. 4c). Moreover, since LRRK2 substrates are involved in membrane trafficking, vesicle-mediated transport was also found to be enhanced in LRRK2 PD. Figure 3c comparison provides an interesting discovery about the difference between iPD and LRRK2 PD gene ontology. In this evaluation, cell adhesion molecule binding, lysosome, leukocyte transendothelial migration, and adaptive immune system were enhanced in LRRK2 PD. Lysosome activity was upregulated due to LRRK2 substrates’ involvement in lysosome sorting, degradation, and autophagy^103^. Leukocyte transendothelial migration, which is crucial for innate immunity and inflammation, and the adaptive immune system, which is carried out by lymphocytes, were enriched, indicating the potential that *LRRK2*-G2019S mutation could further amplify the already pro-inflammatory function of LRRK2 in inflammasome activity^116^. Although the expression of LRRK2 is mainly thought of in the context of neurons, it is also discovered to be highly expressed by immune cells such as monocytes, macrophages, and B cells, where LRRK2 direct substrate-mediated vesicle trafficking is heavily involved in their immune response initiation^116,117^.

Furthermore, the emerging system/network analysis has revolutionized novel mechanism discovery and promising drug targets. Our literature-based network analysis of the gene expression involving these potential biomarkers has revealed the connections between our biomarkers and critical pathways that could lead to PD development. Here, we showed that the four top disease markers, PCSK1N, HNRNPA1, pPLA2G4A, and pLTB4R, are indeed involved in PD pathways such as neuronal cell death, neuroinflammation, autophagy, and formation of amyloid fibrils (Fig. 3e). We also showed that some proteins and phosphoproteins sustained positive or negative correlations with gender, age, disease duration, MoCA, and UPDRS-III. Most interestingly, Fig. 4 displayed PEBP4, NEDD4L, and KLK6 with higher than 0.7 Pearson correlation scores, indicating a strong positive correlation with UPDRS-III. However, we need to emphasize that these correlations with the clinical parameters do not automatically mean causation. Some relevant correlations between the proteins and phosphoproteins and the clinical parameters should be studied further to provide more information.

In total, we discovered a panel of high-confidence putative disease biomarkers, which were substantiated using ROC, machine learning, and in-depth network analysis. The top disease biomarkers, PCSK1N, HNRNPA1, pPLA2G4A, pLTB4R, pPRR15, and pPPFIA1, could be employed for PD detection in a non-invasive way using a simple urine collection (Fig. 5). Our machine learning technique generated an AUC of 94.3%, a confusion matrix accuracy of 87.60%, and a sensitivity of 1 for the top six disease biomarkers (Fig. 6). Higher sensitivity is more important than specificity for early disease diagnosis. Here, we successfully categorized every individual with PD in the test set as PD patients. Given the performance of our machine learning model, there is a potential and feasible clinical application of using the classifier as a diagnostic tool for PD. The machine learning code used for feature selections, model training, and predictive analyses is available in Zenodo (10.5281/zenodo.7679354) and could be easily adapted for other disease classifications^118^. Several potential disease biomarkers have also been validated using targeted approaches—PRM and Western blot, including HNF4A, FN1, APP, APOM, STK11, CD9, CD63, CD81, and PCSK1N (Fig. 7, Supplementary Fig. 10a, b). The PRM-MS is a more powerful method to validate the biomarkers; meanwhile, the immunoassay validation is only semi-quantitative and can be used to look at the overall profile differences between healthy and PD individuals rather than as an absolute measurement. Together, the extensive data on these potential biomarkers might serve as a future of PD detection in a non-invasive and more cost-effective manner and as a resource to the research community for further studies. In other words, this platform represents a foundational resource for the emerging field of accurate and reproducible proteomic biomarker discovery.

We also directed considerable attention to LRRK2 kinase and its Rab substrate proteins in urinary EVs. This project involved two groups of patients with *LRRK2*-G2019S mutation, a feature present in some PD patients. However, it is known that the mutated *LRRK2* does not necessarily lead to PD onset, and many individuals live with this mutation without developing Parkinson’s disease. This study found a minor increase in LRRK2 protein amount and its overall phosphorylation level in PD patients’ urine EVs (Supplementary Fig. 11). Similarly, a few select Rab proteins showed an increased EV signal in total protein amount and phosphorylation level in PD cases (Rab2A, Rab10, pRab12; Supplementary Fig. 12a). However, none were selected as the optimal potential biomarkers for PD diagnosis. This finding further underscores the reality that Parkinson’s disease is highly complicated, with multiple signaling pathways involved in its pathology. While LRRK2 kinase is known to be involved in PD progression, detecting LRRK2 and its direct substrates in urinary EVs may not provide sufficient differentiation between cases. As carried out in this study, a more global analysis, which may or may not be directly influenced by LRRK2 activity, is needed to determine the most statistically significant biomarkers. We advocate that such a comprehensive analysis with highly stringent bioinformatics data validation gives us the best opportunity to discover the most optimal differentiating markers.

Urinary EV proteomics data would generate comprehensive information; however, phosphoproteomics data would provide a more complete picture of the state of the disease. One of the most significant challenges in urinary EV phosphoproteomics exploration is the sometimes-limited volume of urine samples. Here, we could not validate the urinary EV phosphoproteomics results due to the lower volume availability of the new sample batch. Especially in this case, the number and volume of available urine samples from the Columbia University Irving Medical Center (CUIMC) were limited because each patient must be deeply curated to detect the presence of the G2019S mutation. Therefore, we recognize that another more sensitive method to evaluate urinary EV phosphoproteins using lower urine volumes has to be developed to overcome this challenge. Currently, our lab is focusing on overcoming this specific challenge. Furthermore, we acknowledge the limitations of our Western blot experiments. Rather than showing technical replicates (repeats), the Western blot experiments were designed to show biological replicates (different patients) to demonstrate the variation across individuals. Therefore, all of the Western blot experiments were performed only once due to a very limited amount of rare clinical samples. In addition, the ladder on the blots was not visible because the membrane was cut at the proper molecular weight of the correct target proteins to enable us to run multiple antibody blots from the same samples. Moreover, there is no agreement upon loading control for EV samples. When possible, we were able to include CD9 as a marker of isolated EVs to measure the general level of EVs in each urine sample.

In summary, we have developed several comprehensive putative biomarker panels of proteins and phosphoproteins in urinary extracellular vesicles as biosignatures for Parkinson’s disease diagnosis (Table 4). Our putative biomarker panels, supported by prior literature and several validation experiments, offer a great opportunity for further extensive validation studies to translate these potential non-invasive signaling biomarkers as PD biomarkers from urinary EVs. The study highlights our ability to isolate and identify thousands of unique proteins and phosphoproteins from relatively small volumes of urine samples by utilizing the EVtrap EV enrichment approach. These findings further validate the underlying principle that this strategy could be valuable for exploring existing resources in a wide range of diseases. Finally, we expect our immediate results, followed by extensive evaluation and validation of the new markers in the clinical setting, could improve these patients’ medical outcomes and quality of life.

**Table 4:** The summary of all important biomarkers discovered in this study.

Type of biomarkers	Putative biomarkers	Visualized data
Biomarkers of LRRK2 PD vs. control	RAB2A, RAB10, pRAB12	Supplementary Fig. 12a
Biomarkers of LRRK2 PD vs. NMC	PRDX3, KLK6, TRIM17, TPT1, VCAM1, LILRB1, IGF1, PCSK1N, STK11	Fig. 3a
Biomarkers of LRRK2 PD vs. iPD	PRDX3, KLK6, TRIM17, TPT1, VCAM1, LILRB1, HSPA1A, HSPA1B, ECM1, GBA, NEDD4L, GDPD3	Fig. 3b
Biomarkers with strong correlation with UPDRS-III	PEBP4, NEDD4L, KLK6	Fig. 4
Top disease biomarkers chosen by machine learning	PCSK1N, HNRNPA1, pPLA2G4A, pLTB4R, pPRR15, pPPFIA1	Fig. 5
Disease biomarkers validated using PRM-MS	HNRNPA1, HNF4A, FN1, APP, APOM, STK11, CD9, CD63, CD81	Fig. 7
Disease biomarkers validated using WB	HNRNPA1, PCSK1N, STK11	Supplementary Fig. 10a, b
Biomarkers expressed significantly higher in males	ENPEP, GDPD3, NAGA, NEDD4L, QPRT, SCAMP3, RAB1B, RAB7A, RAB3D	Supplementary Figs. 6a and 12b
Biomarkers expressed significantly higher in females	RAB1A	Supplementary Fig. 12b

The listed putative biomarkers were discovered using various approaches, such as Pearson correlation analyses, machine learning, parallel reaction monitoring (PRM-MS), Western blot (WB), and statistical analyses of the protein or phosphoprotein expressions.

## Supplementary information

Description of Additional Supplementary Files

Supplementary Information

Supplementary Data

## Data Availability

The mass spectrometry raw data files and Proteome Discoverer search results for EVtrap repeatability, high-abundant free urine protein depletion, and the discovery experiments have been deposited in the MassIVE database (https://massive.ucsd.edu/ProteoSAFe/static/massive.jsp) and can be accessed via dataset identifier: MSV000085800 | PXD020475. The PRM raw data files and the Skyline file have been deposited in the Panorama Public (https://panoramaweb.org/pd.url). The ProteomeXchange ID reserved for these data is PXD032175. The source data have been provided in files named “Source Data 1” and “Source Data 2” located inside the zipped “Source Data” folder.

## Source data

Source Data
Reporting Summary

## Code availability

Custom Python code used for feature selections, model training, and predictive analyses, along with the README file, input files, and expected results, have been deposited in Zenodo (10.5281/zenodo.7679354)^118^.

## References

[1] Zeng XS, Geng WS, Jia JJ, Chen L, Zhang PP (2018). Cellular and molecular basis of neurodegeneration in Parkinson disease. Front. Aging Neurosci..

[2] Rui Q, Ni H, Li D, Gao R, Chen G (2018). The role of LRRK2 in neurodegeneration of Parkinson disease. Curr. Neuropharmacol..

[3] Houlden H, Singleton AB (2012). The genetics and neuropathology of Parkinson’s disease. Acta Neuropathol..

[4] Adams B (2019). Parkinson’s disease: a systemic inflammatory disease accompanied by bacterial inflammagens. Front. Aging Neurosci..

[5] Tibar H (2018). Non-motor symptoms of Parkinson’s disease and their impact on quality of life in a cohort of Moroccan patients. Front. Neurol..

[6] Zhong, J. et al. Integrated profiling of single cell epigenomic and transcriptomic landscape of Parkinson’s disease mouse brain. Preprint at *bioRxiv*10.1101/2020.02.04.933259 (2020).

[7] Reeve A, Simcox E, Turnbull D (2014). Ageing and Parkinson’s disease: why is advancing age the biggest risk factor?. Ageing Res. Rev..

[8] Hughes RC (1994). Parkinson’s disease and its management. BMJ.

[9] Marras C (2018). Prevalence of Parkinson’s disease across North America. NPJ Parkinsons Dis..

[10] Burbulla LF, Krüger R (2011). Converging environmental and genetic pathways in the pathogenesis of Parkinson’s disease. J. Neurol. Sci..

[11] Chang D (2017). A meta-analysis of genome-wide association studies identifies 17 new Parkinson’s disease risk loci. Nat. Genet..

[12] Satake W (2009). Genome-wide association study identifies common variants at four loci as genetic risk factors for Parkinson’s disease. Nat. Genet..

[13] Simón-Sánchez J (2009). Genome-wide association study reveals genetic risk underlying Parkinson’s disease. Nat. Genet..

[14] Price A, Manzoni C, Cookson MR, Lewis PA (2018). The LRRK2 signalling system. Cell Tissue Res..

[15] Marín I, Egmond WN, Haastert PJM (2008). The Roco protein family: a functional perspective. FASEB J..

[16] Alessi DR, Sammler E (2018). LRRK2 kinase in Parkinson’s disease. Science.

[17] Migheli R (2013). LRRK2 affects vesicle trafficking, neurotransmitter extracellular level and membrane receptor localization. PLoS ONE.

[18] Margolis L, Sadovsky Y (2019). The biology of extracellular vesicles: the known unknowns. PLoS Biol..

[19] Zhang Y, Wu X, Tao WA (2018). Characterization and applications of extracellular vesicle proteome with post-translational modifications. Trends Anal. Chem..

[20] Santucci L (2019). Biological surface properties in extracellular vesicles and their effect on cargo proteins. Sci. Rep..

[21] Yang KS (2017). Multiparametric plasma EV profiling facilitates diagnosis of pancreatic malignancy. Sci. Transl. Med..

[22] Verma M, Lam TK, Hebert E, Divi RL (2015). Extracellular vesicles: potential applications in cancer diagnosis, prognosis, and epidemiology. BMC Clin. Pathol..

[23] Xu R, Greening DW, Zhu H-J, Takahashi N, Simpson RJ (2016). Extracellular vesicle isolation and characterization: toward clinical application. J. Clin. Invest..

[24] Lin J (2015). Exosomes: novel biomarkers for clinical diagnosis. Sci. World J..

[25] Chen IH (2017). Phosphoproteins in extracellular vesicles as candidate markers for breast cancer. Proc. Natl Acad. Sci. USA.

[26] Iliuk A (2020). Plasma-derived extracellular vesicle phosphoproteomics through chemical affinity purification. J. Proteome Res..

[27] Hadisurya, M. et al. Data-independent acquisition phosphoproteomics of urinary extracellular vesicles enables renal cell carcinoma grade differentiation. *Mol. Cell. Proteomics* **22**, 100536 (2023). 10.1016/j.mcpro.2023.100536 PMC10165457 36997065

[28] Bockorny, B. et al. A Large-Scale Proteomics Resource of Circulating Extracellular Vesicles for Biomarker Discovery in Pancreatic Cancer. medRxiv 2023.03.13.23287216 10.1101/2023.03.13.23287216 (2023). 10.7554/eLife.87369 PMC11655061 39693144

[29] Stuendl A (2016). Induction of α-synuclein aggregate formation by CSF exosomes from patients with Parkinson’s disease and dementia with Lewy bodies. Brain.

[30] Cao Z (2019). α-Synuclein in salivary extracellular vesicles as a potential biomarker of Parkinson’s disease. Neurosci. Lett..

[31] Jiang C (2020). Serum neuronal exosomes predict and differentiate Parkinson’s disease from atypical parkinsonism. J. Neurol. Neurosurg. Psychiatry.

[32] Alcalay RN (2020). Higher urine bis(monoacylglycerol)phosphate levels in LRRK2 G2019S mutation carriers: implications for therapeutic development. Mov. Disord..

[33] Decramer S (2008). Urine in clinical proteomics. Mol. Cell. Proteomics.

[34] An M, Gao Y (2015). Urinary biomarkers of brain diseases. Genomics Proteomics Bioinformatics.

[35] Wang S, Kojima K, Mobley JA, West AB (2019). Proteomic analysis of urinary extracellular vesicles reveal biomarkers for neurologic disease. EBioMedicine.

[36] Upadhya R, Shetty AK (2021). Extracellular vesicles for the diagnosis and treatment of Parkinson’s disease. Aging Dis..

[37] Wu X, Li L, Iliuk A, Tao WA (2018). Highly efficient phosphoproteome capture and analysis from urinary extracellular vesicles. J. Proteome Res..

[38] Oberg AL, Vitek O (2009). Statistical design of quantitative mass spectrometry-based proteomic experiments. J. Proteome Res..

[39] Charles Jacob, H. K. et al. Identification of novel early pancreatic cancer biomarkers KIF5B and SFRP2 from “first contact” interactions in the tumor microenvironment. *J. Exp. Clin. Cancer Res.* **41**, 258 (2022). 10.1186/s13046-022-02425-y PMC9400270 36002889

[40] Zeringer E (2013). Methods for the extraction and RNA profiling of exosomes. World J. Methodol..

[41] Rauniyar N (2015). Parallel reaction monitoring: a targeted experiment performed using high resolution and high mass accuracy mass spectrometry. Int. J. Mol. Sci..

[42] Deutsch EW, Lam H, Aebersold R (2008). PeptideAtlas: a resource for target selection for emerging targeted proteomics workflows. EMBO Rep..

[43] MacLean B (2010). Skyline: an open source document editor for creating and analyzing targeted proteomics experiments. Bioinformatics.

[44] Tyanova S (2016). The Perseus computational platform for comprehensive analysis of (prote)omics data. Nat. Methods.

[45] R Core Team. *R: A Language and Environment for Statistical Computing* https://www.R-project.org/ (2022).

[46] Wickham, H. *ggplot2: Elegant Graphics for Data Analysis* (Springer, 2016).

[47] Kassambara, A. *ggpubr: ‘ggplot2’ Based Publication Ready Plots* https://rpkgs.datanovia.com/ggpubr/ (2020).

[48] Blighe, K. *EnhancedVolcano: Publication-Ready Volcano Plots with Enhanced Colouring and Labeling* https://github.com/kevinblighe (2018).

[49] Robin X (2011). pROC: an open-source package for R and S+ to analyze and compare ROC curves. BMC Bioinformatics.

[50] Swinton, J. *Vennerable: Venn and Euler Area-Proportional Diagrams* https://rdrr.io/rforge/Vennerable/ (2019).

[51] Gu Z, Gu L, Eils R, Schlesner M, Brors B (2014). circlize implements and enhances circular visualization in R. Bioinformatics.

[52] Shannon P (2003). Cytoscape: a software environment for integrated models of biomolecular interaction networks. Genome Res..

[53] Szklarczyk D (2019). STRING v11: protein-protein association networks with increased coverage, supporting functional discovery in genome-wide experimental datasets. Nucleic Acids Res..

[54] Krämer A, Green J, Pollard J, Tugendreich S (2014). Causal analysis approaches in ingenuity pathway analysis. Bioinformatics.

[55] Van Rossum, G. & Drake Jr, F. L. *Python Tutorial* https://ir.cwi.nl/pub/5008 (1995).

[56] Anaconda Software Distribution. *Anaconda Documentation* https://docs.anaconda.com/ (2020).

[57] Kluyver, T. et al. in *Positioning and Power in Academic Publishing: Players, Agents and Agendas* (eds Loizides, F. & Schmidt, B.) Jupyter Notebooks—a publishing format for reproducible computational workflows, 87–90 (IOS Press, 2016).

[58] McKinney, W. *Data Structures for Statistical Computing in Python*, 56–61 10.25080/Majora-92bf1922-00a (2010).

[59] Harris CR (2020). Array programming with NumPy. Nature.

[60] Hunter JD (2007). Matplotlib: a 2D graphics environment. Comput. Sci. Eng..

[61] Plotly Technologies Inc. *Collaborative Data Science* https://plot.ly (2015).

[62] Pedregosa F (2011). Scikit-learn: machine learning in Python. J. Mach. Learn. Res..

[63] Raschka S (2018). MLxtend: providing machine learning and data science utilities and extensions to Python’s scientific computing stack. J. Open Source Softw..

[64] Chen, T. & Guestrin, C. XGBoost: a scalable tree boosting system. In *Proc. 22nd ACM SIGKDD International Conference on Knowledge Discovery and Data Mining*, Vol. 42, 785–794 (ACM, 2016).

[65] Hastie, T., Tibshirani, R. & Friedman, J. *The Elements of Statistical Learning: Data Mining, Inference, and Prediction*, 57–60 (Springer, 2009).

[66] Buljan M, Blattmann P, Aebersold R, Boutros M (2018). Systematic characterization of pan‐cancer mutation clusters. Mol. Syst. Biol..

[67] Le Large TYS (2017). Key biological processes driving metastatic spread of pancreatic cancer as identified by multi-omics studies. Semin. Cancer Biol..

[68] Pishvaian MJ (2018). Molecular profiling of patients with pancreatic cancer: initial results from the know your tumor initiative. Clin. Cancer Res..

[69] Chen F, Chandrashekar DS, Varambally S, Creighton CJ (2019). Pan-cancer molecular subtypes revealed by mass-spectrometry-based proteomic characterization of more than 500 human cancers. Nat. Commun..

[70] Chen F (2017). Multiplatform-based molecular subtypes of non-small-cell lung cancer. Oncogene.

[71] Li J (2017). Characterization of human cancer cell lines by reverse-phase protein arrays. Cancer Cell.

[72] Mundt F (2018). Mass spectrometry–based proteomics reveals potential roles of NEK9 and MAP2K4 in resistance to PI3K inhibition in triple-negative breast cancers. Cancer Res..

[73] Wulfkuhle JD (2012). Molecular analysis of HER2 signaling in human breast cancer by functional protein pathway activation mapping. Clin. Cancer Res..

[74] Wulfkuhle, J. D. et al. Evaluation of the HER/PI3K/AKT family signaling network as a predictive biomarker of pathologic complete response for patients with breast cancer treated with Neratinib in the I-SPY 2 TRIAL. *JCO Precis. Oncol*. **2**, 1–20 (2018). 10.1200/PO.18.00024 PMC7446527 32914002

[75] Zagorac I (2018). In vivo phosphoproteomics reveals kinase activity profiles that predict treatment outcome in triple-negative breast cancer. Nat. Commun..

[76] Huang KL (2017). Proteogenomic integration reveals therapeutic targets in breast cancer xenografts. Nat. Commun..

[77] Wang S (2017). Elevated LRRK2 autophosphorylation in brain-derived and peripheral exosomes in LRRK2 mutation carriers. Acta Neuropathol. Commun..

[78] Fraser KB, Moehle MS, Alcalay RN, West AB (2016). Urinary LRRK2 phosphorylation predicts parkinsonian phenotypes in G2019S LRRK2 carriers. Neurology.

[79] Fraser KB (2016). Ser(P)–1292 LRRK2 in urinary exosomes is elevated in idiopathic Parkinson’s disease. Mov. Disord..

[80] Virreira Winter, S. et al. Urinary proteome profiling for stratifying patients with familial Parkinson’s disease. *EMBO Mol. Med*. **13**, e13257 (2021). 10.15252/emmm.202013257 PMC7933820 33481347

[81] Alcalay RN (2013). Parkinson disease phenotype in Ashkenazi jews with and without LRRK2 G2019S mutations. Mov. Disord..

[82] Keerthikumar S (2016). ExoCarta: a web-based compendium of exosomal cargo. J. Mol. Biol..

[83] Théry C (2018). Minimal information for studies of extracellular vesicles 2018 (MISEV2018): a position statement of the International Society for Extracellular Vesicles and update of the MISEV2014 guidelines. J. Extracell. Vesicles.

[84] Zhao M (2017). A comprehensive analysis and annotation of human normal urinary proteome. Sci. Rep..

[85] Uhlen M (2015). Tissue-based map of the human proteome. Science.

[86] Zhu Q (2021). The genetic source tracking of human urinary exosomes. Proc. Natl Acad. Sci. USA.

[87] Videira PAQ, Castro-Caldas M (2018). Linking glycation and glycosylation with inflammation and mitochondrial dysfunction in Parkinson’s disease. Front. Neurosci..

[88] Trezzi JP (2017). Distinct metabolomic signature in cerebrospinal fluid in early Parkinson’s disease. Mov. Disord..

[89] Everse J, Liu CJJ, Coates PW (2011). Physical and catalytic properties of a peroxidase derived from cytochrome c. Biochim. Biophys. Acta.

[90] Loeffler DA, Camp DM, Conant SB (2006). Complement activation in the Parkinson’s disease substantia nigra: an immunocytochemical study. J. Neuroinflammation.

[91] Sasaki M (2006). Neuromelanin magnetic resonance imaging of locus ceruleus and substantia nigra in Parkinson’s disease. Neuroreport.

[92] Sun Y, Vashisht AA, Tchieu J, Wohlschlegel JA, Dreier L (2012). Voltage-dependent anion channels (VDACs) recruit parkin to defective mitochondria to promote mitochondrial autophagy. J. Biol. Chem..

[93] Klein AD, Mazzulli JR (2018). Is Parkinson’s disease a lysosomal disorder?. Brain.

[94] Sarkar C (2019). PLA2G4A/cPLA2-mediated lysosomal membrane damage leads to inhibition of autophagy and neurodegeneration after brain trauma. Autophagy.

[95] Cerri S, Mus L, Blandini F (2019). Parkinson’s disease in women and men: what’s the difference?. J. Parkinsons. Dis..

[96] Baldereschi M (2000). Parkinson’s disease and parkinsonism in a longitudinal study: two-fold higher incidence in men. Neurology.

[97] Vásquez KA, Valverde EM, Aguilar DV, Gabarain HJH (2019). Montreal cognitive assessment scale in patients with Parkinson disease with normal scores in the mini-mental state examination. Dement. Neuropsychol..

[98] Safari S, Baratloo A, Elfil M, Negida A (2016). Evidence based emergency medicine; Part 5 receiver operating curve and area under the curve. Emergency.

[99] Santiago JA, Potashkin JA (2015). Network-based metaanalysis identifies HNF4A and PTBP1 as longitudinally dynamic biomarkers for Parkinson’s disease. Proc. Natl Acad. Sci. USA.

[100] Vacchi E (2020). Immune profiling of plasma-derived extracellular vesicles identifies Parkinson disease. Neurol. Neuroimmunol. Neuroinflamm..

[101] Di Maio R (2018). LRRK2 activation in idiopathic Parkinson’s disease. Sci. Transl. Med..

[102] Petridi, S. et al. Neurodegeneration caused by LRRK2-G2019S requires Rab10 in select dopaminergic neurons. Preprint at *bioRxiv*10.1101/586073 (2019).

[103] Seol W, Nam D, Son I (2019). Rab GTPases as physiological substrates of LRRK2 kinase. Exp. Neurobiol..

[104] Cova I, Priori A (2018). Diagnostic biomarkers for Parkinson’s disease at a glance: where are we?. J. Neural Transm..

[105] Angeles DC (2011). Mutations in LRRK2 increase phosphorylation of peroxiredoxin 3 exacerbating oxidative stress-induced neuronal death. Hum. Mutat..

[106] Pampalakis G (2017). KLK6 proteolysis is implicated in the turnover and uptake of extracellular alpha-synuclein species. Oncotarget.

[107] Lassot I (2018). The E3 ubiquitin ligases TRIM17 and TRIM41 modulate α-synuclein expression by regulating ZSCAN21. Cell Rep..

[108] Kim JM (2007). Identification of genes related to Parkinson’s disease using expressed sequence tags. DNA Res..

[109] Yu CC (2020). Vascular inflammation is a risk factor associated with brain atrophy and disease severity in Parkinson’s disease: a case-control study. Oxid. Med. Cell. Longev..

[110] Wu Z (2023). A computational approach based on weighted gene co-expression network analysis for biomarkers analysis of Parkinson’s disease and construction of diagnostic model. Front. Comput. Neurosci..

[111] Bernhard FP (2016). Insulin-like growth factor 1 (IGF-1) in Parkinson’s disease: Potential as trait-, progression- and prediction marker and confounding factors. PLoS ONE.

[112] Hauser MA (2005). Expression profiling of substantia nigra in Parkinson disease, progressive supranuclear palsy, and frontotemporal dementia with parkinsonism. Arch. Neurol..

[113] Kwon DH (2022). Cerebrospinal fluid metabolome in Parkinson’s disease and multiple system atrophy. Int. J. Mol. Sci..

[114] Sidransky E, Lopez G (2012). The link between the GBA gene and parkinsonism. Lancet Neurol..

[115] Song J, Kim J (2016). Degeneration of dopaminergic neurons due to metabolic alterations and Parkinson’s disease. Front. Aging Neurosci..

[116] Gardet A (2010). LRRK2 is involved in the IFN-γ response and host response to pathogens. J. Immunol..

[117] Hakimi M (2011). Parkinson’s disease-linked LRRK2 is expressed in circulating and tissue immune cells and upregulated following recognition of microbial structures. J. Neural Transm..

[118] Hadisurya, M. et al. Quantitative proteomics and phosphoproteomics of urinary extracellular vesicles define diagnostic biosignatures for Parkinson’s disease. Zenodo 10.5281/zenodo.7679354 (2023). 10.1038/s43856-023-00294-w PMC10172329 37165152

