# Supplementary material for: Quantitative proteomics and phosphoproteomics of urinary extracellular vesicles define putative diagnostic biosignatures for Parkinson’s disease
Source: Commun Med (Lond). 2023 May 10;3:64. doi: 10.1038/s43856-023-00294-w (PMC10172329; doi:10.1038/s43856-023-00294-w)
Supplement: Supplementary file 1 — Description of Additional Supplementary Files [file 43856_2023_294_MOESM1_ESM.pdf]

**Description of Additional Supplementary Files**

**File Name:** Supplementary Data 1

**Description:** Before Bioinformatics Analysis: All identified and quantified urinary EV proteins from 6 different replicates with their normalized abundances. These data were then analyzed statistically to determine the reproducibility of our overall EVtrap-LCMS method. EV markers (as per ExoCarta) were denoted with red font.

After Bioinformatics Analysis: All quantified urinary EV proteins with their coefficient of variations (CVs) after bioinformatics analysis. The proteins were ranked from the highest to the lowest CVs.

Urine vs. EV Raw Abundances: All identified direct urine and urinary EV proteins with their raw abundances.

Urine vs. EV Transformed: All quantified direct urine and urinary EV proteins with their log-based 2 transformed abundances.

**File Name:** Supplementary Data 2

**Description:** Proteins: All identified EV proteins. Some proteins have several isoforms. The total number of unique identified proteins is 4,476.

Peptide Groups: All identified EV peptide groups. There are 46,240 peptide groups in total.

**File Name:** Supplementary Data 3

**Description:** Phosphoproteins: All identified EV phosphoproteins. Some phosphoproteins have several isoforms. The total number of unique identified phosphoproteins is 2,680.

Phosphopeptide Groups: All identified EV phosphopeptide groups. There are 10,620 phosphopeptide groups in total.

**File Name:** Supplementary Data 4

**Description:** NMC vs. Control: The complete proteomic data of the NMC vs. Control volcano plot.

iPD vs. Control: The complete proteomic data of the iPD vs. Control volcano plot.

LRRK2 PD vs. Control: The complete proteomic data of the LRRK2 PD vs. Control volcano plot.

LRRK2 PD vs. NMC: The complete proteomic data of the LRRK2 PD vs. NMC volcano plot.

LRRK2 PD vs iPD: The complete proteomic data of the LRRK2 PD vs. iPD volcano plot.

Statistical Data: The p-value, the q-value, the difference, and the fold-change for all previous four comparisons.

**File Name:** Supplementary Data 5

**Description:** NMC vs. Control: The complete phosphoproteomic data of the NMC vs. Control volcano plot.

iPD vs. Control: The complete phosphoproteomic data of the iPD vs. Control volcano plot.

LRRK2 PD vs. Control: The complete phosphoproteomic data of the LRRK2 PD vs. Control volcano plot.

Statistical Data: The p-value, the q-value, the difference, and the fold-change for all previous three comparisons.

**File Name:** Supplementary Data 6

**Description:** NMC vs. Control: Significant gene ontology terms for up-regulated proteins in NMC vs. Control.

iPD vs. Control: Significant gene ontology terms for up-regulated proteins in iPD vs. control.

LRRK2 PD vs. Control: Significant gene ontology terms for up-regulated proteins in LRRK2 PD vs. Control.

LRRK2 PD vs. NMC: Significant gene ontology terms for up-regulated proteins in LRRK2 PD vs. NMC.

LRRK2 PD vs IPD: Significant gene ontology for upregulated proteins in LRRK2 PD vs. iPD.

**File Name:** Supplementary Data 7

**Description:** Discovery Experiment: The complete information of cohort demographics and clinical characteristics for all 82 patients.

PRM Experiment: The complete information of cohort demographics and clinical characteristics for all 36 patients.

Immunoassay Experiment: The complete information of cohort demographics and clinical characteristics for all 20 patients.

**File Name:** Supplementary Data 8

**Description:** Circos plot data containing the protein list and their respective gene ontology.

**File Name:** Supplementary Data 9

**Description:** Disease Markers: The IPA analysis of the protein disease markers.

**File Name:** Supplementary Data 10

**Description:** GPCR MAPK: The String analysis of PLA2G4A and LTB4R phosphoproteins in their involvement in GPCR and MAPK signaling pathways.

Lysosome: The String analysis of NEU1 phosphoproteins in their involvement in lysosomal regulation.

**File Name:** Supplementary Data 11

**Description:** Phospho-Disease Markers: The IPA analysis of the phosphoprotein disease markers related to the autophagy pathway.

**File Name:** Supplementary Data 12

**Description:** NMC vs. Control: The significant proteomic data of the NMC vs. Control volcano plot.

iPD vs. Control: The significant proteomic data of the iPD vs. Control volcano plot.

LRRK2 PD vs. Control: The significant proteomic data of the LRRK2 PD vs. Control volcano plot.

LRRK2 PD vs. NMC: The significant proteomic data of the LRRK2 PD vs. NMC volcano plot.

**File Name:** Supplementary Data 13

**Description:** NMC vs. Control: The significant phosphoproteomic data of the NMC vs. Control volcano plot.

iPD vs. Control: The significant phosphoproteomic data of the iPD vs. Control volcano plot.

LRRK2 PD vs. Control: The significant phosphoproteomic data of the LRRK2 PD vs. Control volcano plot.

**File Name:** Supplementary Data 14

**Description:** Proteins: The list of all overlapping up-regulated proteins in Supplementary Fig. 9a.

Phosphoproteins: The list of all overlapping up-regulated phosphoproteins in Supplementary Fig. 9b.

**File Name:** Supplementary Data 15

**Description:** Training Set - Disease: The list of all potential protein and phosphoprotein disease biomarkers and their normalized abundances needed for the feature selection.

Test Set - Disease: The list of all potential protein and phosphoprotein disease biomarkers and their normalized abundances needed for the predictive ability estimation.

**File Name:** Supplementary Data 16

**Description:** PRM Inclusion List: The inclusion list uploaded to the PRM method in Q-Exactive HF-X mass spectrometer.

PRM Result: The Skyline output of the PRM data after normalization to A2M.

Western Blot: The Western blot analysis of STK11, PCSK1N, and HNRNPA1 normalized to CD9

**File Name:** Supplementary Data 17

**Description:** The western blot data analysis of CD9, total LRRK2, and pSer1292-LRRK2.

**File Name:** Source Data 1

**Description:** The numerical results underlying the graphs and charts presented in the main and supplementary figures.

**File Name:** Source Data 2

**Description:** The uncropped blot/gel images after being sliced according to the appropriate molecular weights of the target proteins, and antibodies used.
