# Supplementary material for: Quantitative proteomics and phosphoproteomics of urinary extracellular vesicles define putative diagnostic biosignatures for Parkinson’s disease
Source: Commun Med (Lond). 2023 May 10;3:64. doi: 10.1038/s43856-023-00294-w (PMC10172329; doi:10.1038/s43856-023-00294-w)
Supplement: Supplementary file 2 — Supplementary Information [file 43856_2023_294_MOESM2_ESM.pdf]

Quantitative proteomics and phosphoproteomics of urinary extracellular vesicles define putative diagnostic biosignatures for Parkinson's Disease

Marco Hadisurya[1], Li Li[2], Kananart Kuwaranancharoen[3], Xiaofeng Wu[4], Zheng-Chi Lee[1,5], Roy N. Alcalay[6], Shalini Padmanabhan[7], W. Andy Tao[1,2,4,8,9*], Anton Iliuk[1, 2*]

[1]Department of Biochemistry, Purdue University, West Lafayette, IN, USA 47907

[2]Tymora Analytical Operations, West Lafayette, IN, USA 47906

[3]School of Electrical and Computer Engineering, Purdue University, West Lafayette, IN, USA 47907

[4]Department of Chemistry, Purdue University, West Lafayette, IN, USA 47907

[5]West Lafayette Junior/Senior Highschool, West Lafayette, IN, USA 47906

[6]Department of Neurology, Columbia University Irving Medical Center, New York, NY, USA 10032

[7]The Michael J. Fox Foundation for Parkinson's Research, New York City, NY, USA 10163

[8]Department of Medicinal Chemistry and Molecular Pharmacology, Purdue University, West Lafayette, IN, USA 47907

[9]Purdue Institute for Cancer Research, Purdue University, West Lafayette, IN, USA 47907

[*]To whom correspondence should be addressed. Email: watao@purdue.edu; anton.iliuk@tymora-analytical.com

Supplementary Figure 1

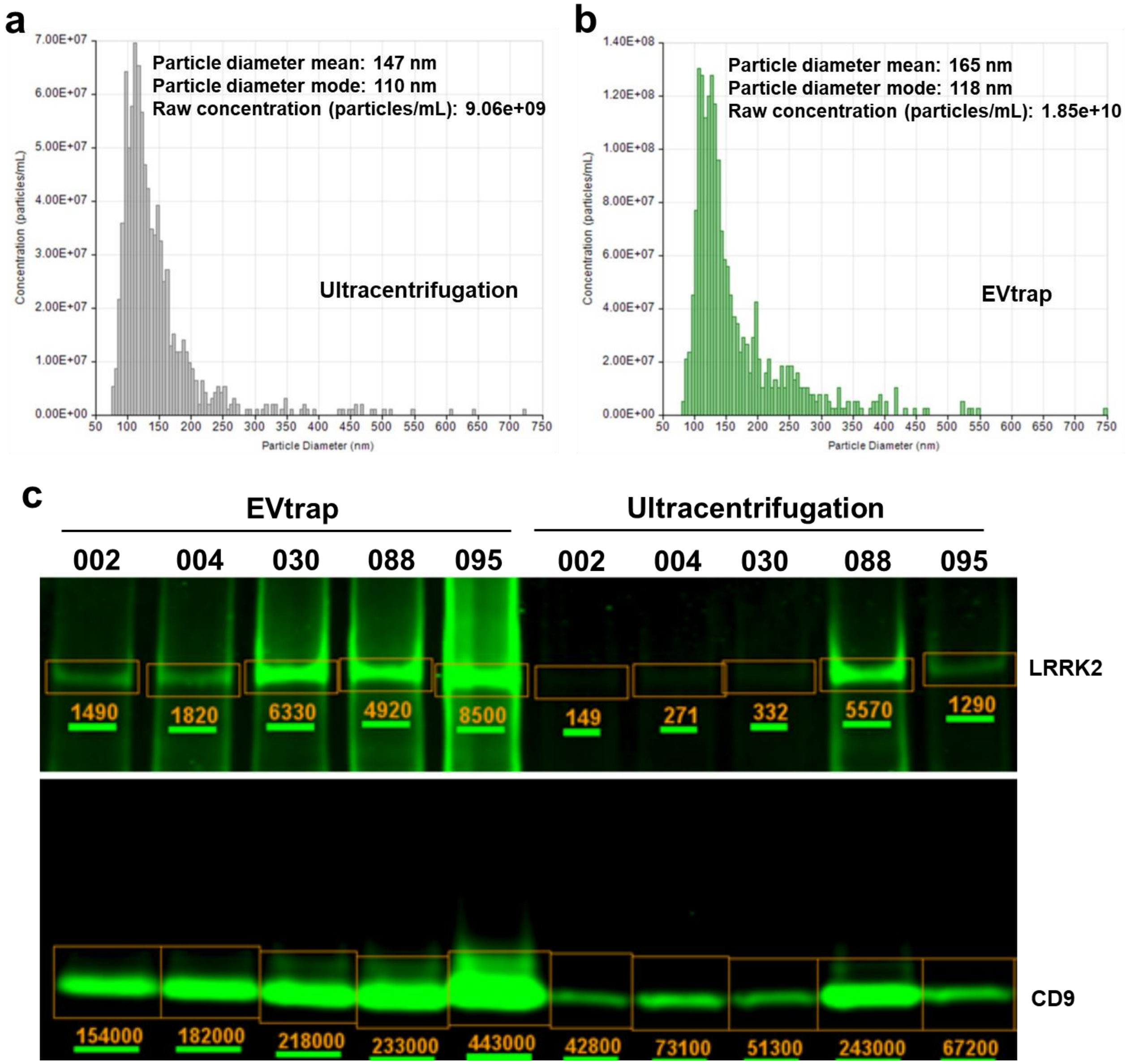

Supplementary Figure 1. Comparison of EVtrap and ultracentrifugation for EV capture from urine. TRPS analysis of EVs captured by a) ultracentrifugation or b) EVtrap. c) Western blot detection of CD9 and LRRK2 proteins of 5 urine EV samples isolated by EVtrap or ultracentrifugation. The membranes were cut according to the appropriate molecular weights to detect the target proteins at their corresponding molecular weights. The Western blot experiments were performed once with samples from 5 different individuals.

Supplementary Figure 2

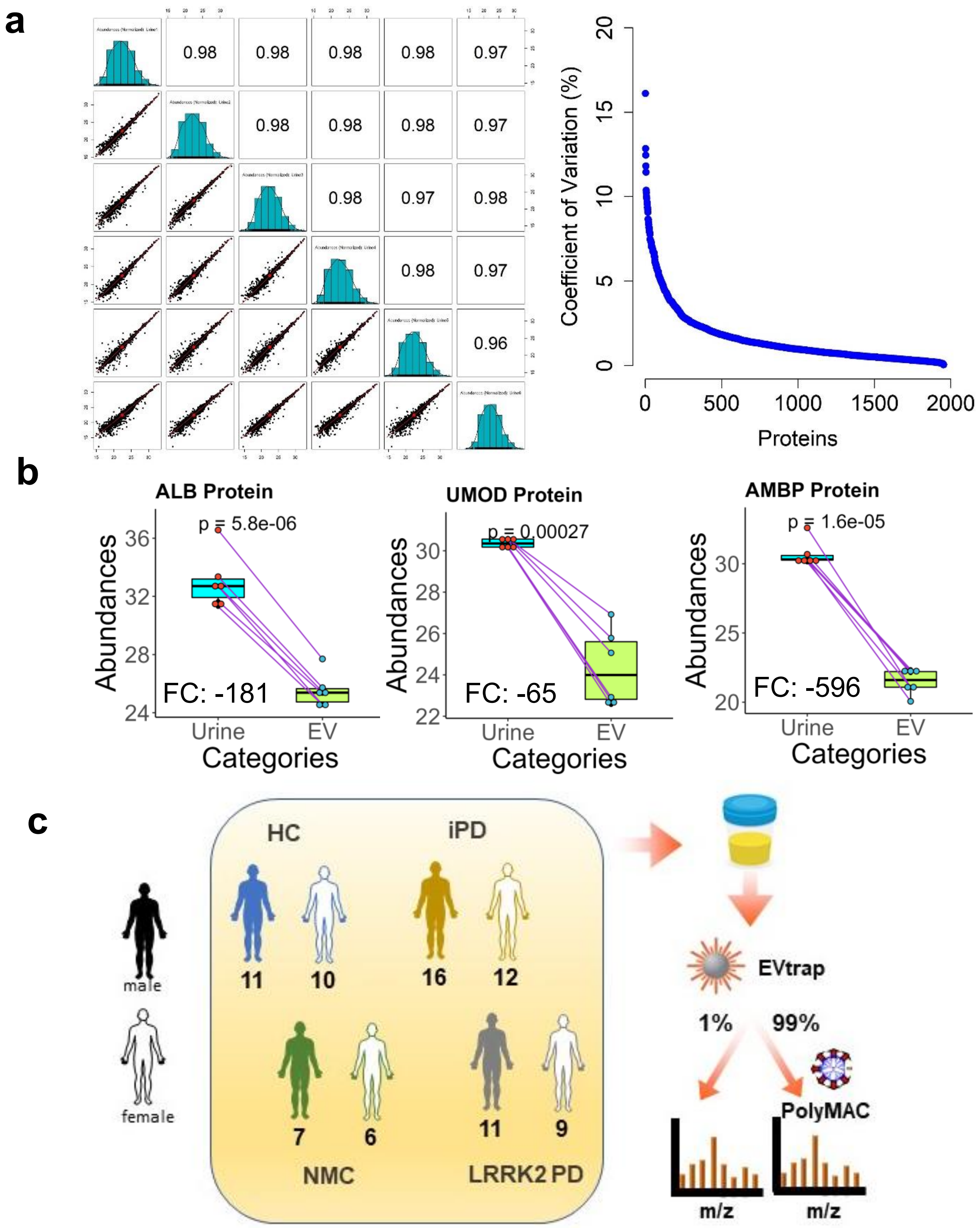

Supplementary Figure 2. The reproducibility evaluation of our complete urine EV analysis protocol and the analytical sample preparation workflow. To evaluate the procedural reproducibility, a single urine sample was separated into six aliquots and processed with our EVtrap-LCMS protocol as 6 technical replicates. a) We created a multi-scatter plot accompanied by Pearson correlation coefficients and a distribution plot of proteins by a coefficient of variation (%). b) The high-abundant free urine protein depletion after urinary EVs isolation was

analyzed using 6 pairs of direct urine and urinary EVs isolated using EVtrap, where each pair was from the same urine sample with the same volume (paired Student's two-tailed t-test p-values were shown). For the lines in box plots: the line inside the box is the 50th percentile (median), the bottom and top of the box are the 25th and 75th percentiles, and the whiskers are the 95% confidence interval. c) Workflow for urine sample processing.

Supplementary Figure 3

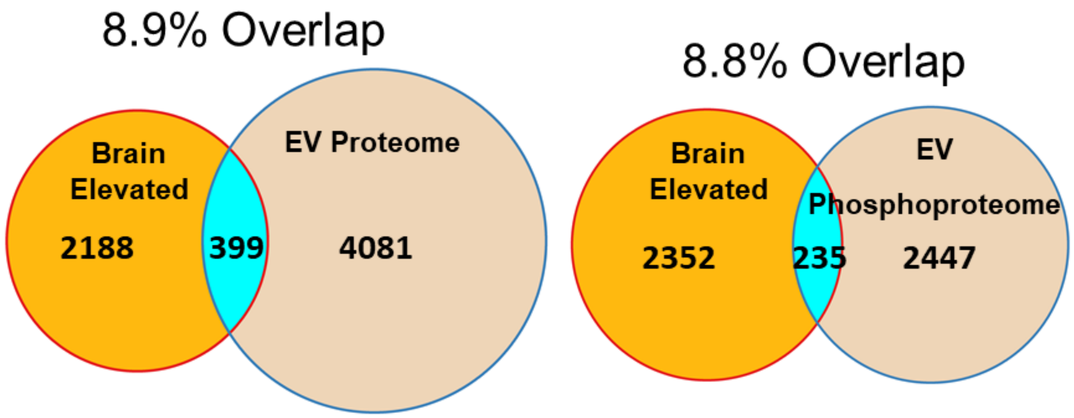

Supplementary Figure 3. The overlap of our identified proteomic and phosphoproteomic data with available brain-elevated RNA-seq data downloaded from the Human Protein Atlas website. We used 2587 proteins classified as brain-elevated from the Human Protein Atlas for comparison with our identified EV proteins and phosphoproteins.

Supplementary Figure 4

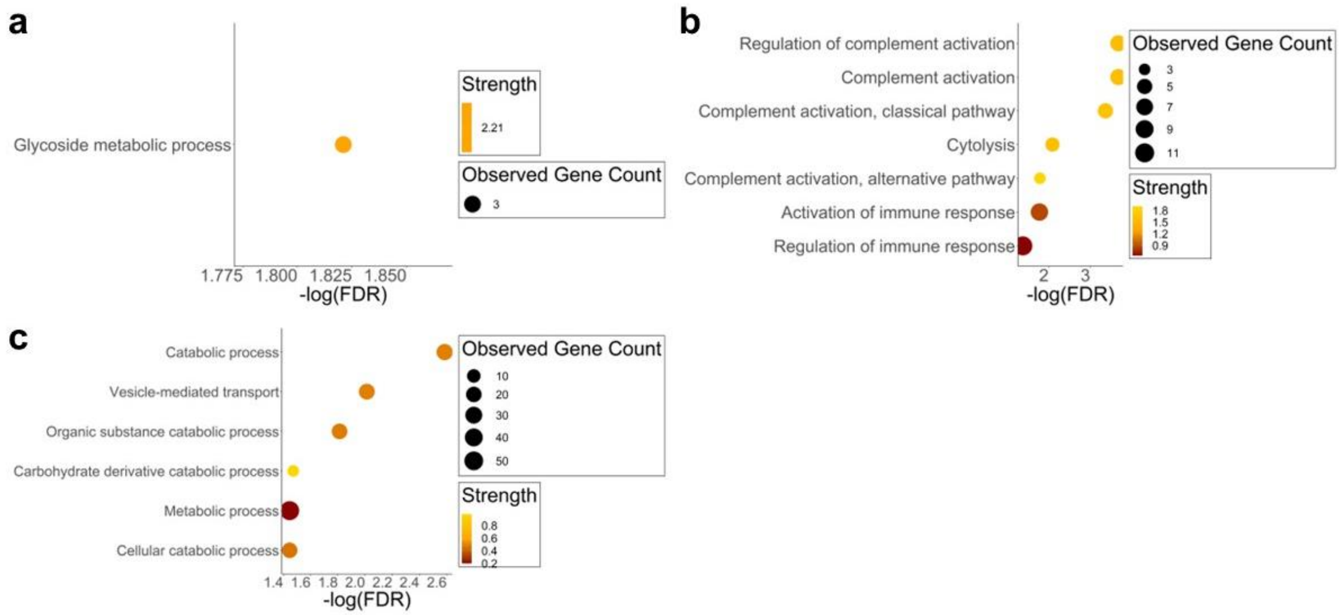

Supplementary Figure 4. Enriched biological process gene ontology analyses of up-regulated proteins. GO analyses for a) NMC compared to HC; b) iPD compared to HC; and c) LRRK2 PD compared to HC. The analyses were carried out with the STRING database. The gene ontology analyses were set with a threshold FDR of 5% after Benjamin-Hochberg correction.

Supplementary Figure 5

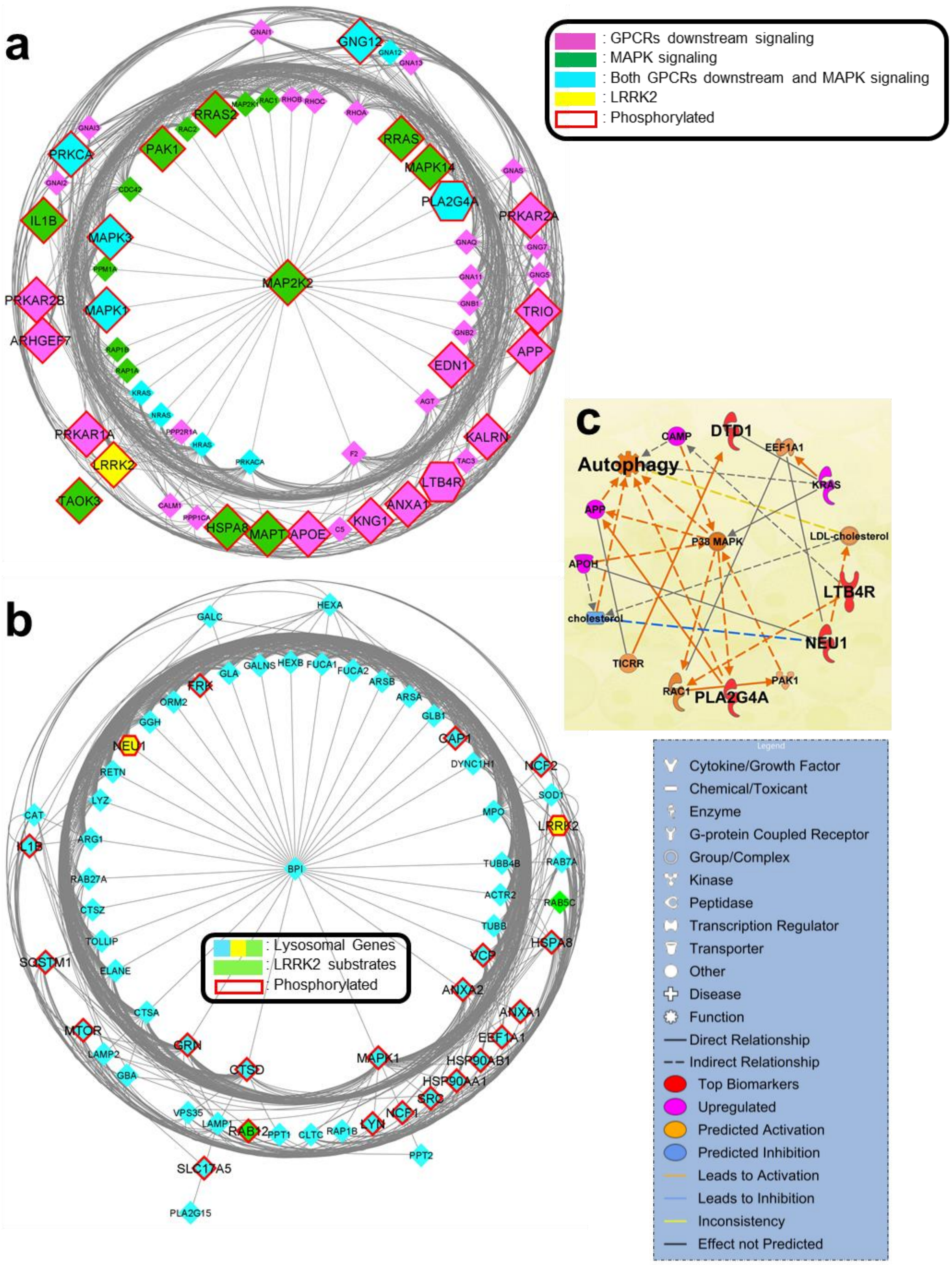

Supplementary Figure 5. Phosphoprotein disease biomarker network and pathway analyses. Enriched networks include a) GPCRs and MAPK signaling pathways and b) lysosome regulation and lysosomal disorder. c) IPA pathway analysis of the phosphoprotein disease markers related to the autophagy pathway.

Supplementary Figure 6

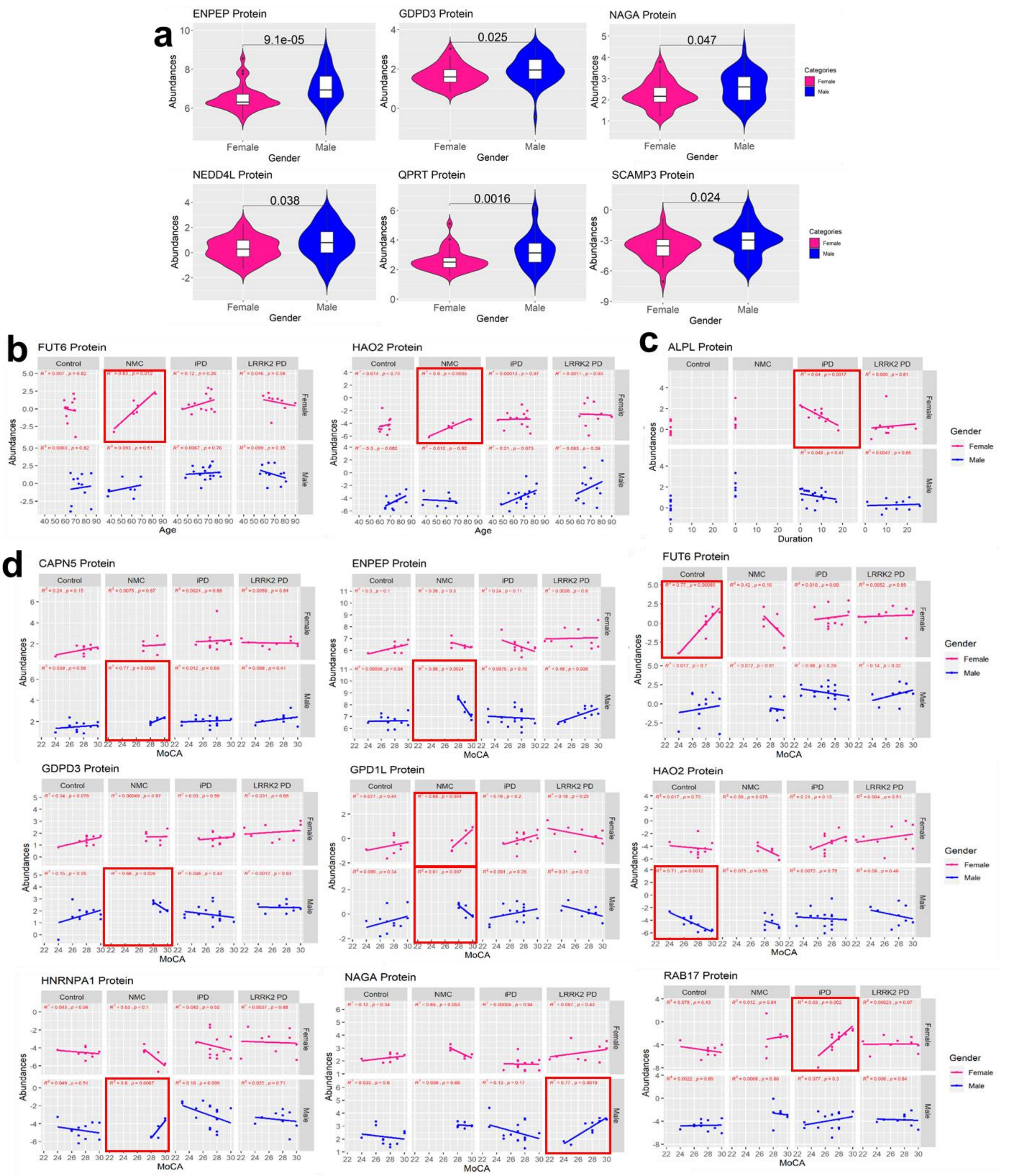

Supplementary Figure 6. Correlation analysis for select potential protein biomarkers from the full data set (training and test sets). a) ENPEP, GDPD3, NAGA, NEDD4L, QPRT, and SCAMP3 proteins were expressed higher in males (female/male = 37/45, the unpaired two-sample Wilcoxon test p-values were shown). For the lines in box plots inside the violin plots: the line inside the box is the 50th percentile (median), the bottom and top of the box are the 25th and 75th percentiles, and the whiskers are the 95% confidence interval. b) The correlation analysis between FUT6 and HAO2 protein abundances and age for each group according to gender.

c) The correlation analysis between ALPL protein abundances and disease duration for each group according to gender. d) The correlation analysis between CAPN5, ENPEP, FUT6, GDPD3, GPD1L, HAO2, HNRNPA1, NAGA, and RAB17 protein abundances and MoCA for each group according to gender. The red-bordered areas show either positive or negative correlations. The correlations between potential biomarker expressions with gender, age, disease duration, and MoCA were created with a minimal 0.6 for R^2 and a maximal 0.05 for p-value calculated using t-distribution with n-2 degrees of freedom as thresholds. All correlation analyses were derived from 21 HC, 13 NMC, 28 iPD, and 20 LRRK2 PD (female/male = 37/45).

Supplementary Figure 7

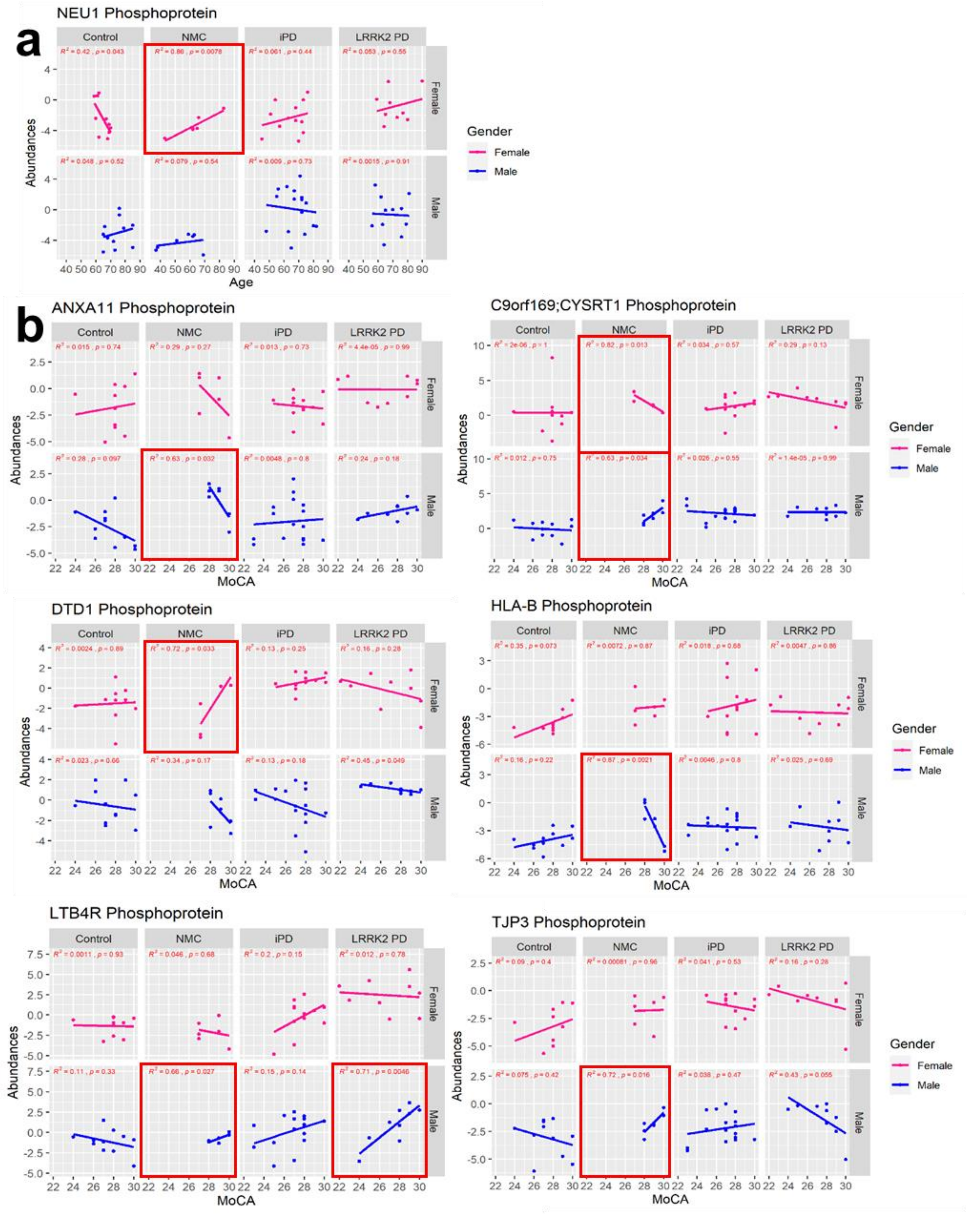

Supplementary Figure 7. Correlation analysis for select potential phosphoprotein biomarkers from the full data set (training and test sets). a) The correlation analysis between NEU1 phosphoprotein abundances and age for each group according to gender. b) The correlation analysis between ANXA11, CYSRT1, DTD1, HLA-B, LTB4R, and TJP3 phosphoprotein abundances and MoCA for each group according to gender. The red-bordered areas show either positive or negative correlations. The correlations between potential biomarker

expressions with age and MoCA were created with a minimal 0.6 for R^2 and a maximal 0.05 for p-value calculated using t-distribution with n-2 degrees of freedom as thresholds. All correlation analyses were derived from 21 HC, 13 NMC, 28 iPD, and 20 LRRK2 PD (female/male = 37/45).

Supplementary Figure 8

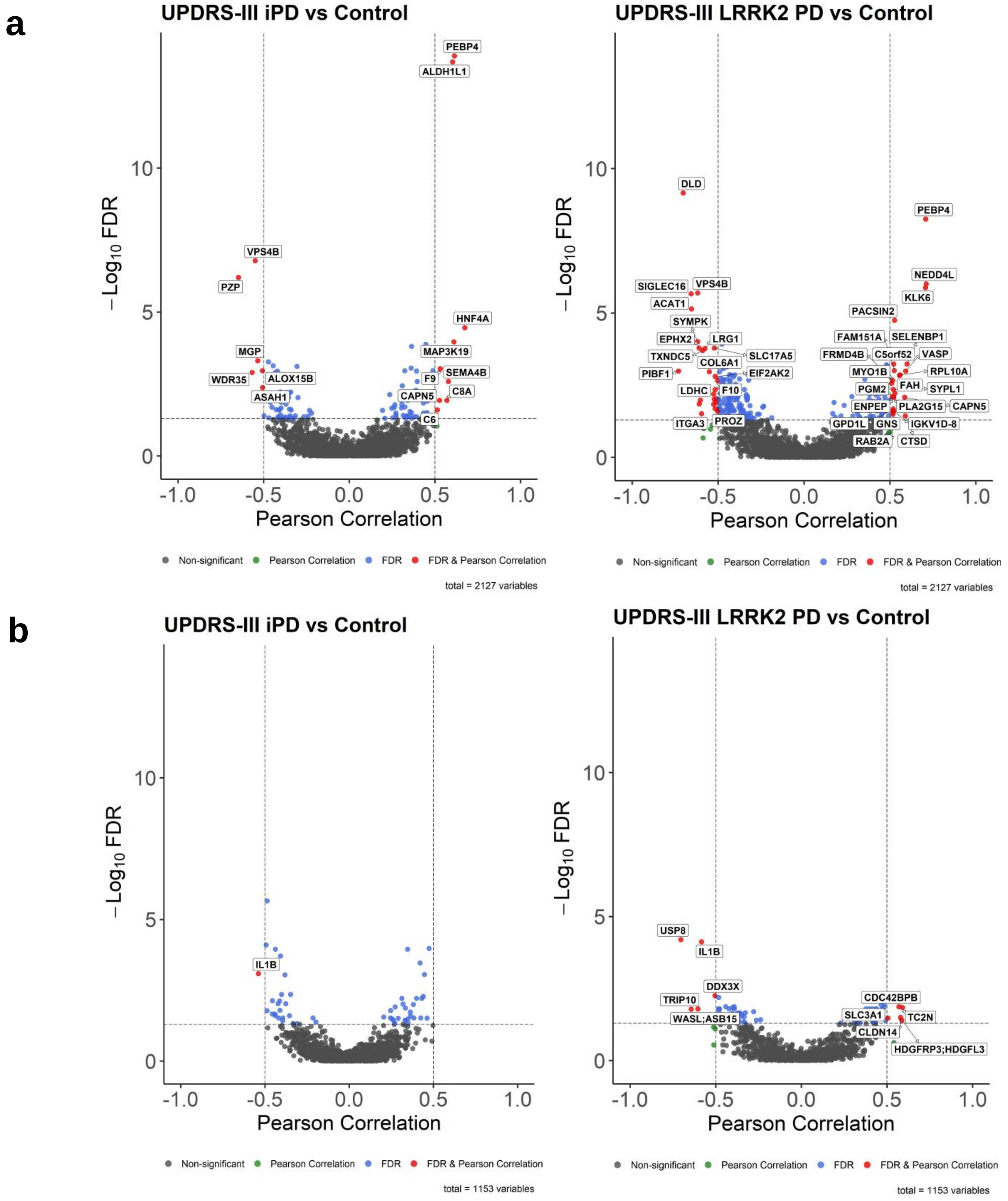

Supplementary Figure 8. Correlations with clinical parameter, UPDRS-III. Pearson correlation scores and associated FDR-values [-$\log_{10}$] of all a) protein and b) phosphoprotein intensities with the UPDRS-III score. Either 28 iPD patients (left) or 20 LRRK2 PD patients (right) versus 21 HC were included. Significantly correlated proteins with an FDR of 5% after Benjamin-Hochberg correction and Pearson correlation of more than 0.5 are labeled.

Supplementary Figure 9

a

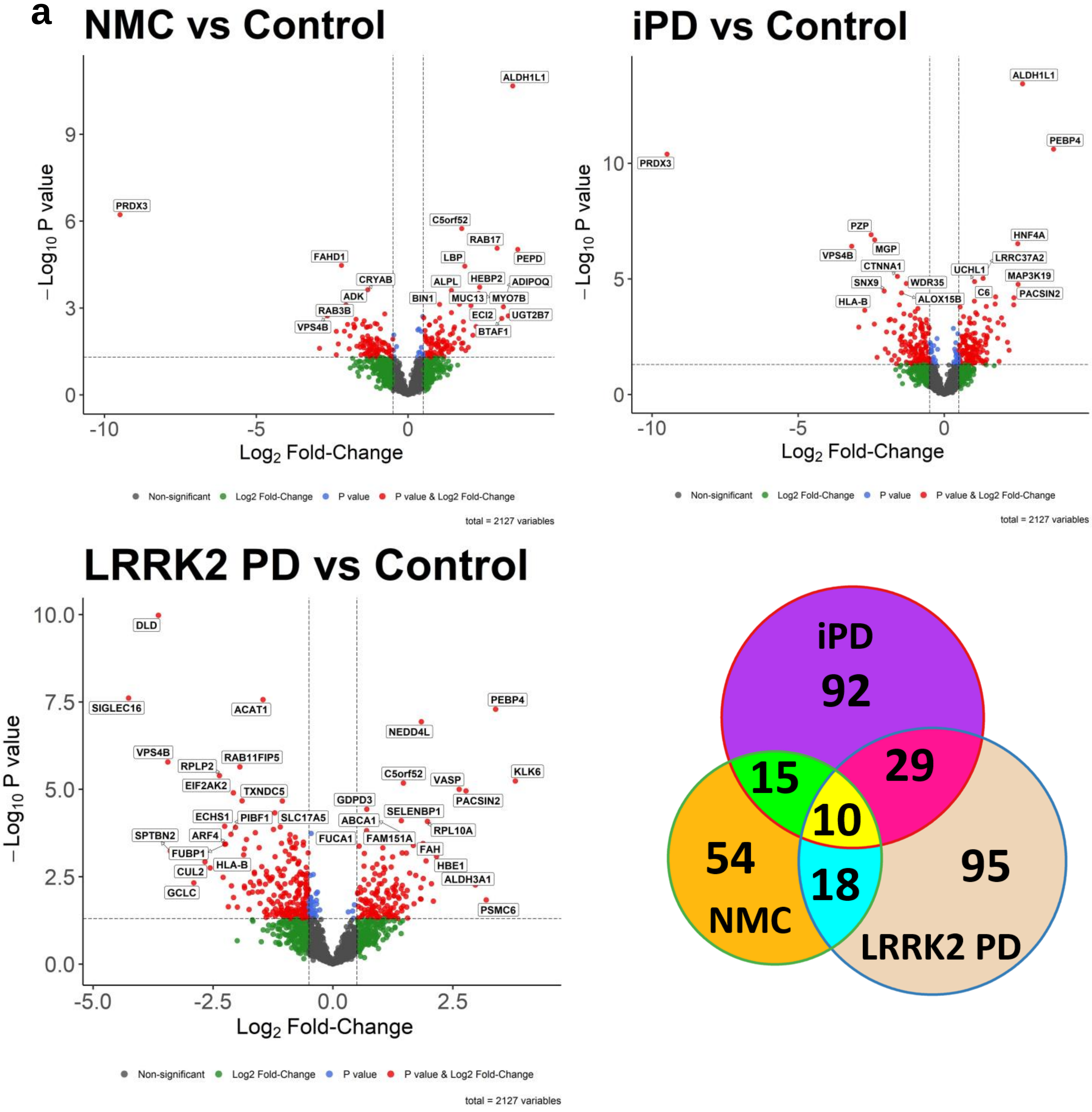

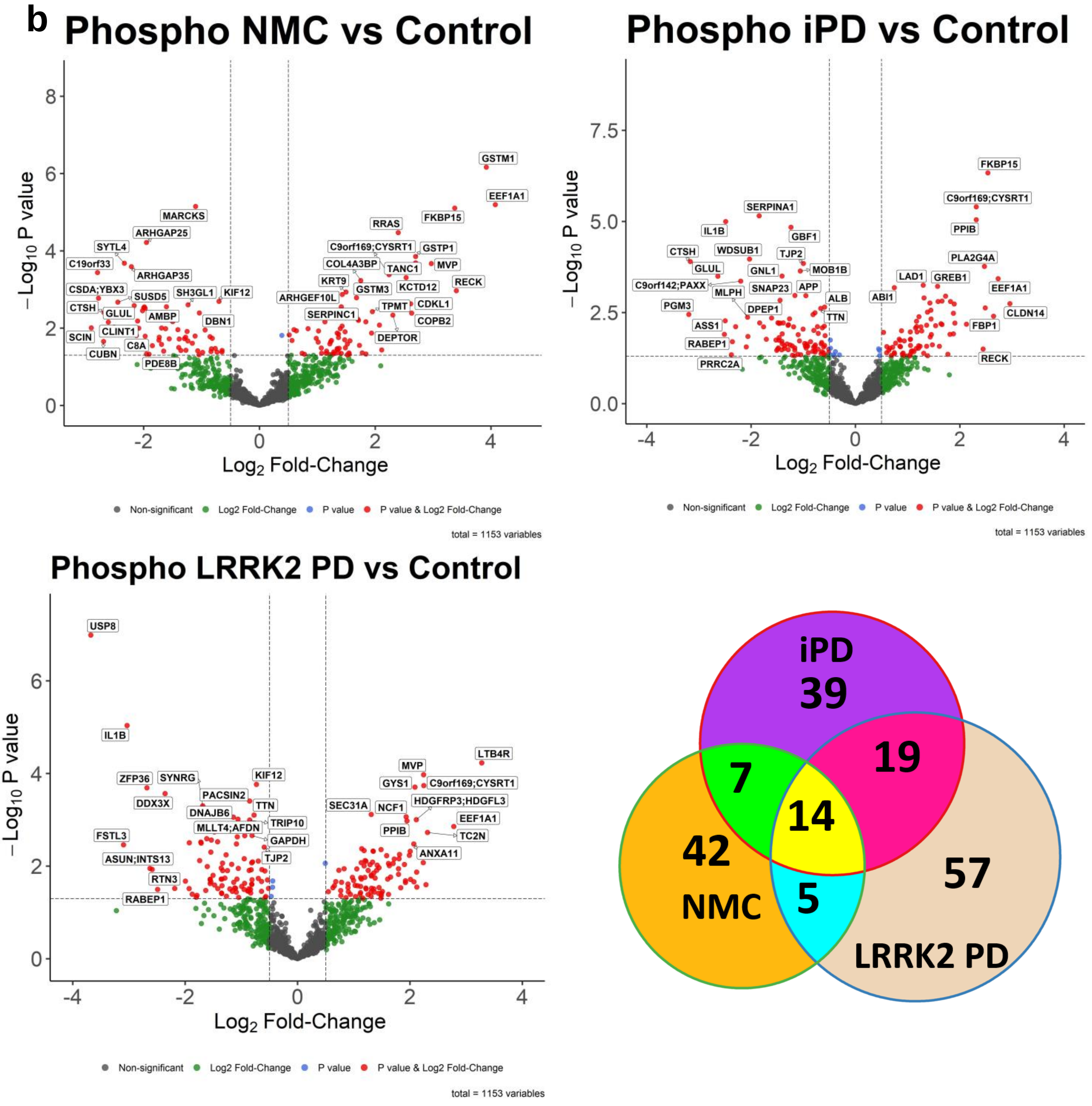

Supplementary Figure 9. Biosignature study design created on the training set. All three categories: NMC, iPD, and LRRK2 PD were compared to the HC group for (a) proteins and (b) phosphoproteins (Training set: 15 HC, 9 NMC, 19 iPD, and 14 LRRK2 PD). Volcano plots were created for each comparison with cut-off values of Student's two-tailed t-test p-value = 0.05 and log base 2 fold-change = 0.5, which equals to ~1.414 fold-change. Significantly up-regulated phosphoproteins from the three volcano plots were overlapped in Venn diagrams (see Supplementary Data 14 for overlapping proteins and phosphoproteins).

Supplementary Figure 10

a

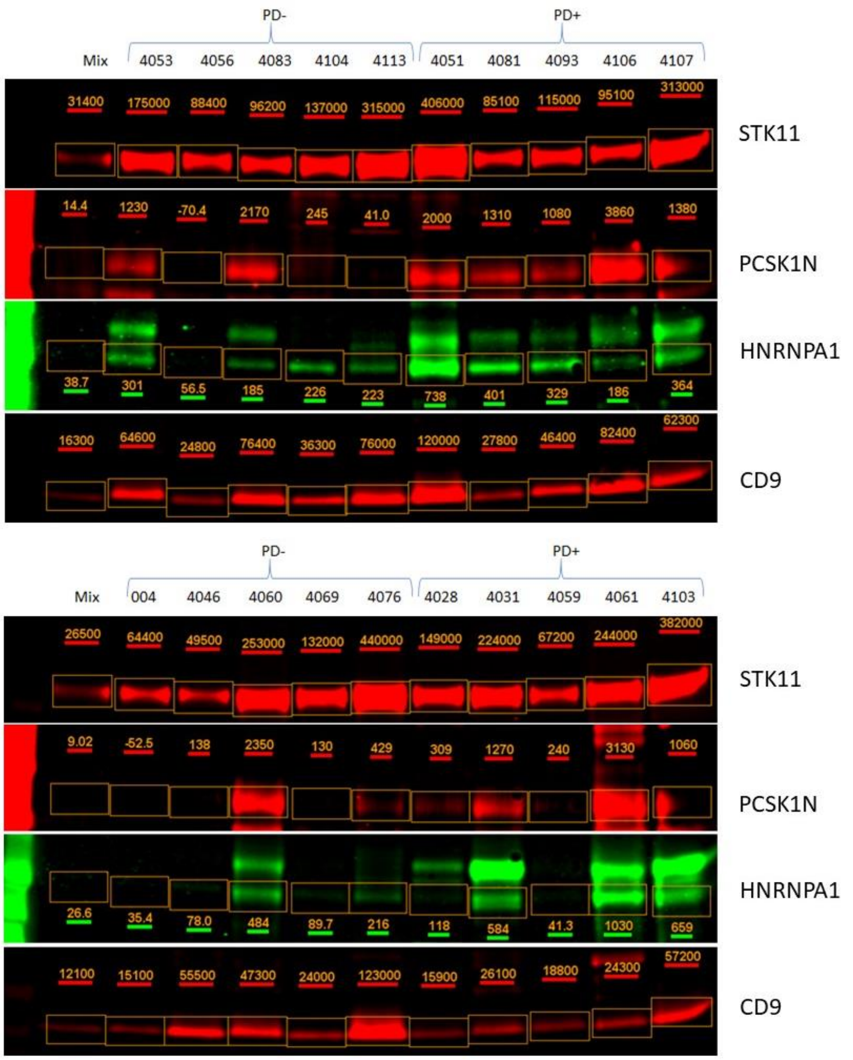

b

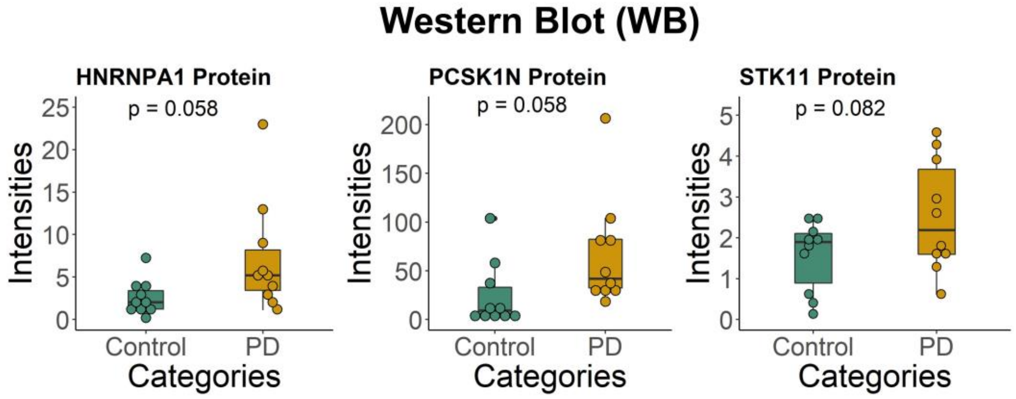

Supplementary Figure 10. Western blot analysis of CD9, STK11, PCSK1N, and HNRNPA1. a) All 20 urine EV samples (10 patients with PD and 10 healthy individuals) were analyzed by Western blot with anti-CD9, anti-STK11, anti-PCSK1N, and anti-HNRNPA1 antibodies (2 blots for each type). An equal amount of pooled urine EVs was loaded in lane 1 of each gel. The membranes were cut according to the appropriate molecular weights to detect the target proteins at their corresponding molecular weights. The Western blot experiments were performed once with samples from 20 different individuals. b) Two top disease biomarkers, HNRNPA1 and PCSK1N, and a potential disease biomarker, STK11, were validated in 10 patients with PD and 10 healthy individuals using Western blot (p-value < 0.1). The Student's two-tailed t-test calculated all p-values. For the lines in box plots: the line inside the box is the 50th percentile (median), the bottom and top of the box are the 25th and 75th percentiles, and the whiskers are the 95% confidence interval.

Supplementary Figure 11

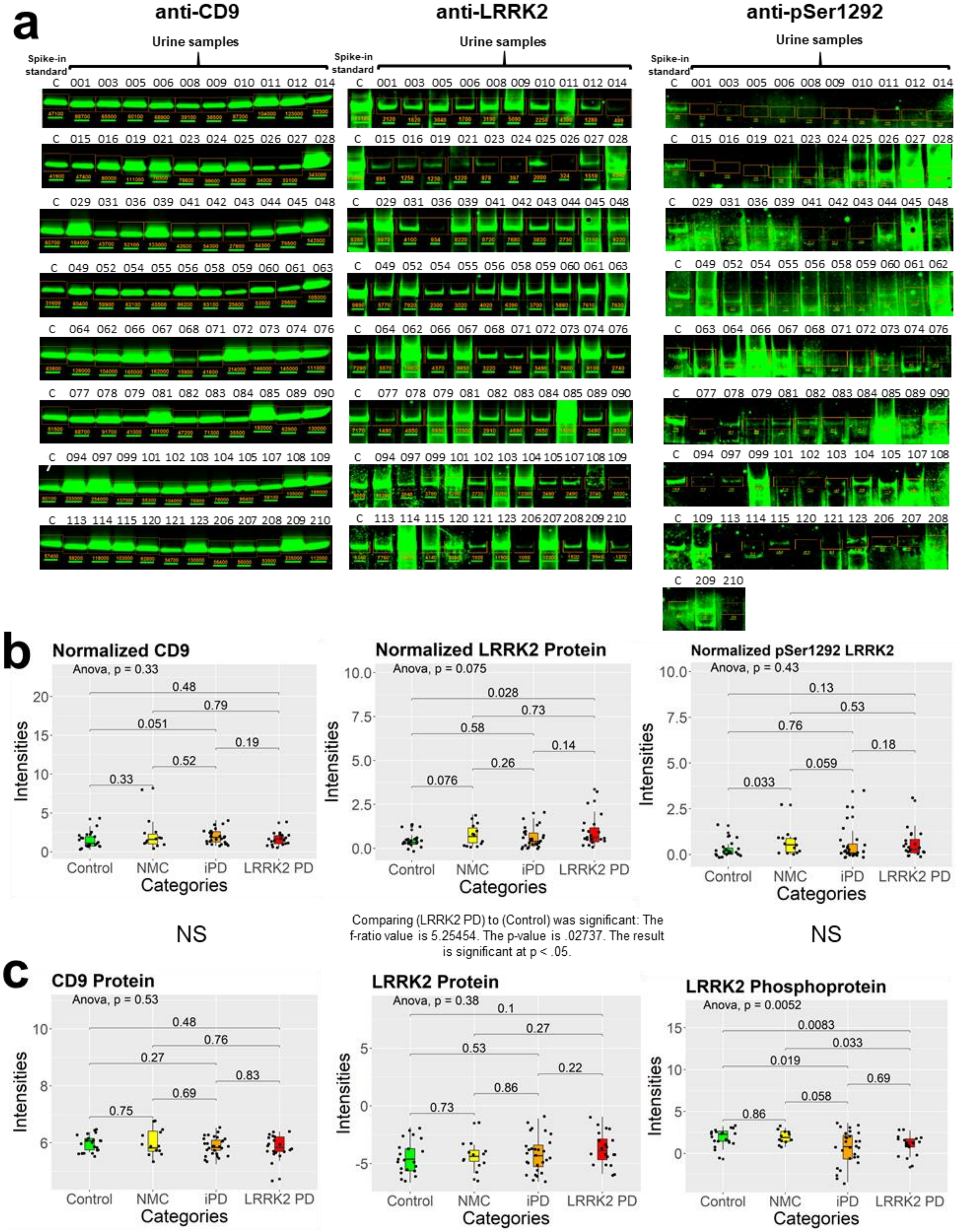

Supplementary Figure 11. Western blot analysis of CD9, total LRRK2, and pSer1292-LRRK2. All 82 urine EV samples (21 HC, 13 NMC, 28 iPD, and 20 LRRK2 PD) were analyzed by Western blot with anti-CD9, anti-LRRK2, and anti-pSer1292-LRRK2 antibodies (8 blots for each type) and mass spectrometer. An equal amount of a spike-in standard was loaded in lane 1 of each gel (pooled urine EVs for CD9 blots, recombinant LRRK2 for LRRK2 blots, and autophosphorylated recombinant LRRK2 for pSer1292-LRRK2 blots). a) Western blots from each analyzed target protein. The membranes were cut according to the appropriate molecular weights to detect the target proteins at their corresponding molecular weights. The Western blot experiments were performed once with samples from 82 different individuals. b) Western blot-based quantitative comparison across all samples

after normalization with an internal standard (See Supplementary Data 17 for table format). c) Mass spectrometry-based quantitative comparison across all samples after normalization with an internal standard. The Student's two-tailed t-test p-values and the one-way ANOVA p-value were included on each boxplot. For the lines in box plots: the line inside the box is the 50th percentile (median), the bottom and top of the box are the 25th and 75th percentiles, and the whiskers are the 95% confidence interval.

Supplementary Figure 12

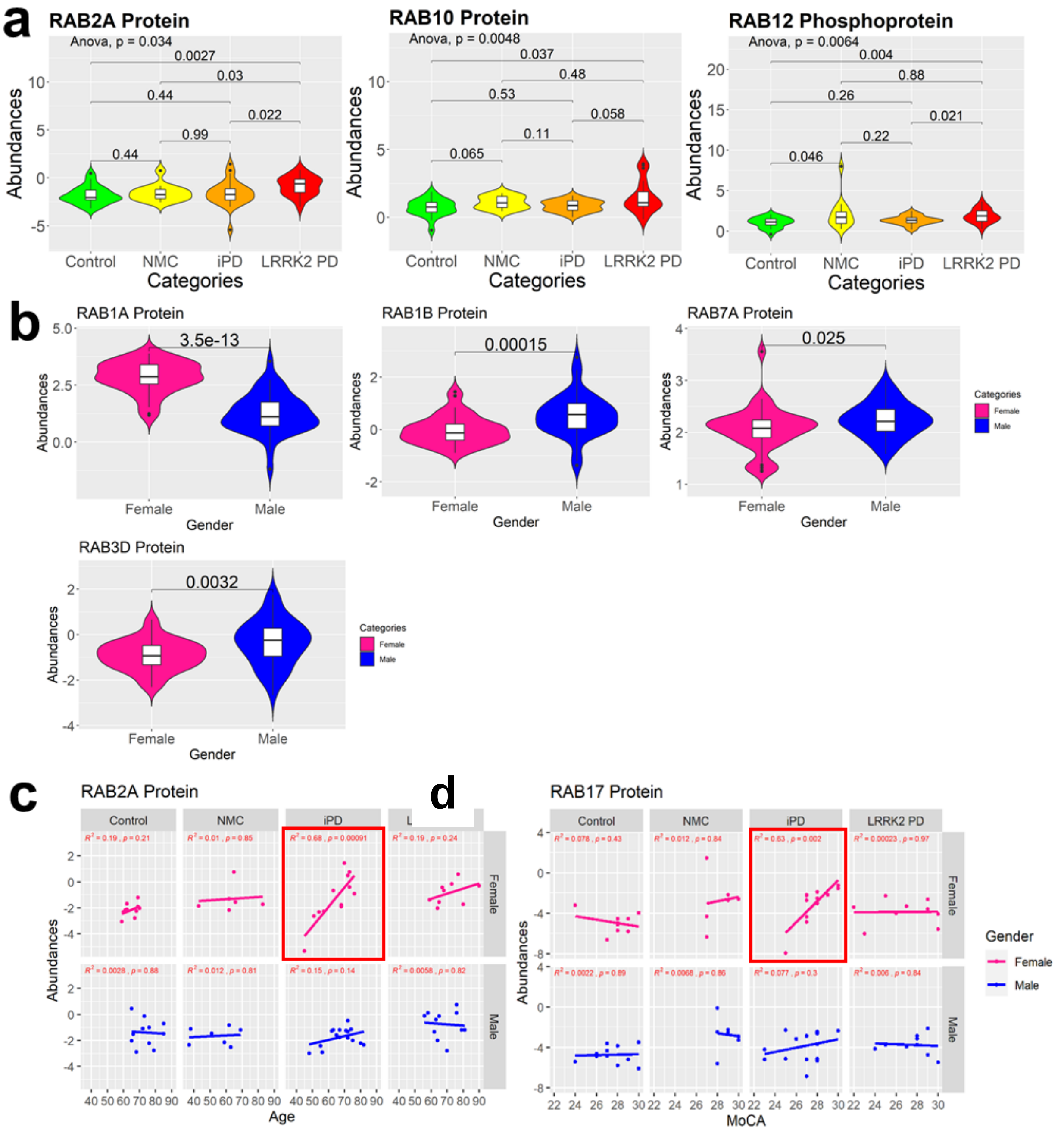

Supplementary Figure 12. Significantly changing Rab proteins and phosphoproteins. a) The EV levels of Rab2A and Rab10 in LRRK2 PD were significantly higher than the control. The violin plots were derived from 21 HC, 13 NMC, 28 iPD, and 20 LRRK2 PD. The unpaired two-sample Wilcoxon test p-values and the one-way ANOVA p-value were included for each violin plot. b) Rab1A was expressed at lower levels in males; Rab1B, Rab3D, and Rab7A were expressed at higher levels in males (female/male = 37/45, the unpaired two-sample Wilcoxon test p-values were shown). For the lines in box plots inside the violin plots: the line inside the box is the 50th percentile (median), the bottom and top of the box are the 25th and 75th percentiles, and the whiskers are the 95% confidence interval. c) The correlation analysis between Rab2A protein abundances and age for each group according to gender. d) The correlation analysis between Rab17 protein abundances and MoCA for each group according to gender. The red-bordered areas show positive correlations. The correlations between potential biomarker expressions with gender, age, and MoCA were created with a minimal 0.6 for R^2 and a maximal 0.05 for p-value calculated using t-distribution with n-2 degrees of freedom as thresholds. The correlation analyses were derived from 21 HC, 13 NMC, 28 iPD, and 20 LRRK2 PD (female/male = 37/45).

Supplementary Table 1

Gene Name	Reason
HNRNPA1	Top Disease Biomarker
PCSK1N	Top Disease Biomarker
IGF1	Potential Disease Biomarker
STK11	Potential Disease Biomarker
FN1	Co-purified with EV
A2M	EV Marker
ALB	Common EV contaminant
CD63	EV Marker
CD81	EV Marker
CD9	EV Marker
MSN	EV Marker
APOE	Involved in PD Pathway
APOM	Involved in PD Pathway
APOA1	Known PD Biomarker
APP	Known PD Biomarker
HNF4A	Known PD Biomarker
PARK7	Known PD Biomarker

Supplementary Table 1. List of the proteins targeted by PRM-MS.

Supplementary Table 2

Proteins		
Accession	Gene Symbol	# PSMs
P61026	RAB10	1740
Q15907	RAB11B	1400
Q6IQ22	RAB12	37
P51153	RAB13	171
P61106	RAB14	612
Q9H0T7	RAB17	26
Q9NP72	RAB18	25
P62820	RAB1A	622
Q9H0U4	RAB1B	828
Q9NX57	RAB20	2
Q9UL25	RAB21	166
Q9UL26	RAB22A	55
Q9ULC3	RAB23	24
P57735	RAB25	107
P51159-1	RAB27A	271
O00194	RAB27B	159
P61019-1	RAB2A	273
Q13636	RAB31	3
Q13637	RAB32	8
Q9BZG1	RAB34	5
Q15286	RAB35	437
P20337	RAB3B	127
O95716	RAB3D	331
Q86YS6	RAB43	38
P20338	RAB4A	38
P20339	RAB5A	433
P61020	RAB5B	564
P51148-2	RAB5C	1444
P20340-2	RAB6A	58
P51149	RAB7A	1861
O14966-1	RAB7L1; RAB29	268
P61006	RAB8A	1016
Q92930	RAB8B	576
P51151	RAB9A	24

Phosphoproteins		
Accession	Gene Symbol	# PSMs
P61026	RAB10	57
Q6IQ22	RAB12	384
P51153	RAB13	13
Q14966-1	RAB7L1; RAB29	135
Q86YS6	RAB43	3
P51149	RAB7A	2
P61006	RAB8A	290
Q92930	RAB8B	34

LRRK2 Substrates

Quantified

Supplementary Table 2. List of the 34 identified Rab GTPases, 12 known as LRRK2 substrates, and eight phosphorylated Rab GTPases.
