# Supplementary material for: Quantitative proteomics and phosphoproteomics of urinary extracellular vesicles define putative diagnostic biosignatures for Parkinson’s disease
Source: Commun Med (Lond). 2023 May 10;3:64. doi: 10.1038/s43856-023-00294-w (PMC10172329; doi:10.1038/s43856-023-00294-w)
Supplement: Supplementary file 4 — Source Data [file 43856_2023_294_MOESM4_ESM.zip › Source Data 2.pptx]

## Slide 1
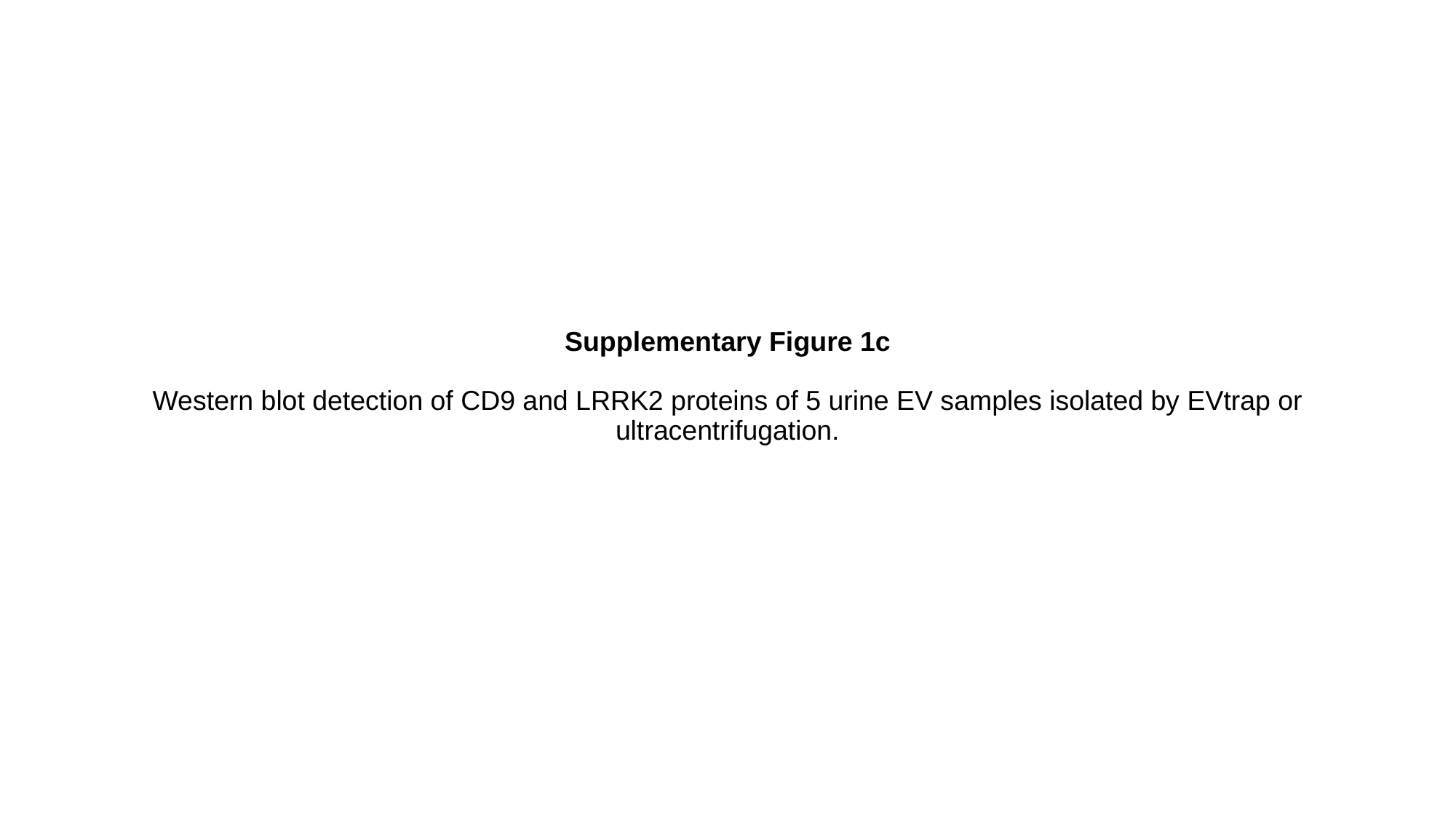

# Supplementary Figure 1c Western blot detection of CD9 and LRRK2 proteins of 5 urine EV samples isolated by EVtrap or ultracentrifugation.

## Slide 2
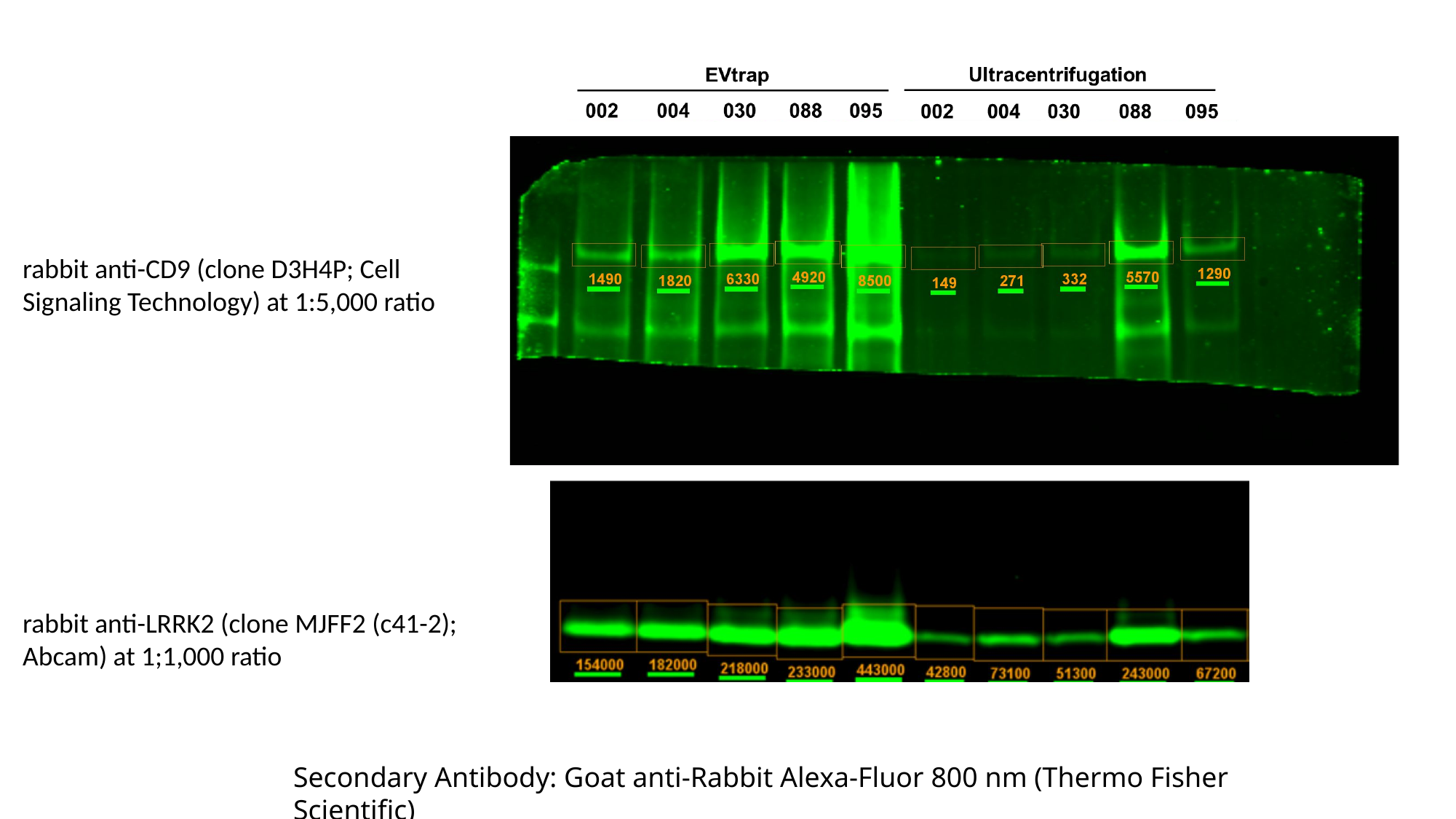

rabbit anti-CD9 (clone D3H4P; Cell Signaling Technology) at 1:5,000 ratio
rabbit anti-LRRK2 (clone MJFF2 (c41-2); Abcam) at 1;1,000 ratio
Secondary Antibody: Goat anti-Rabbit Alexa-Fluor 800 nm (Thermo Fisher Scientific)

## Slide 3
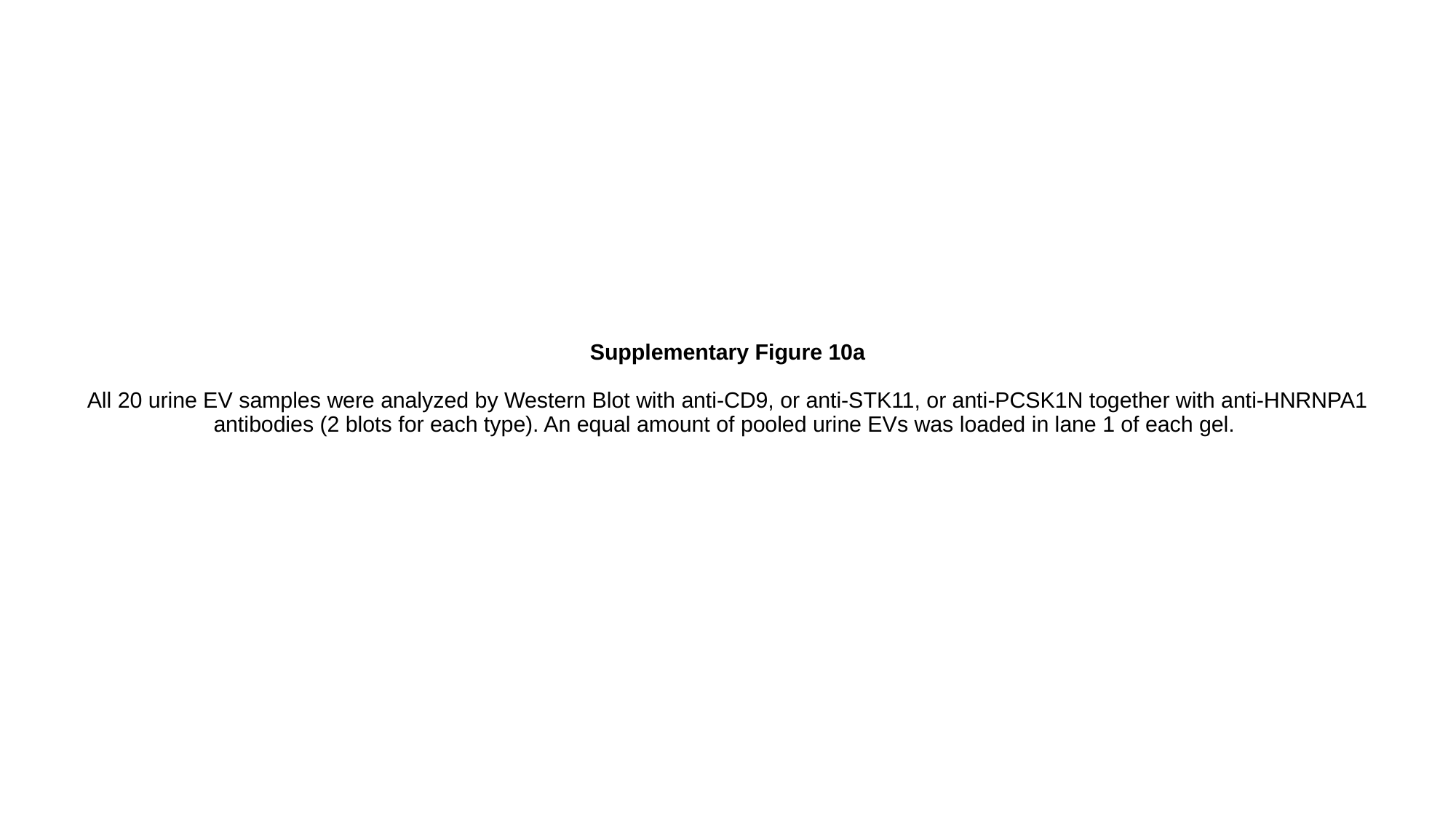

# Supplementary Figure 10a All 20 urine EV samples were analyzed by Western Blot with anti-CD9, or anti-STK11, or anti-PCSK1N together with anti-HNRNPA1 antibodies (2 blots for each type). An equal amount of pooled urine EVs was loaded in lane 1 of each gel.

## Slide 4
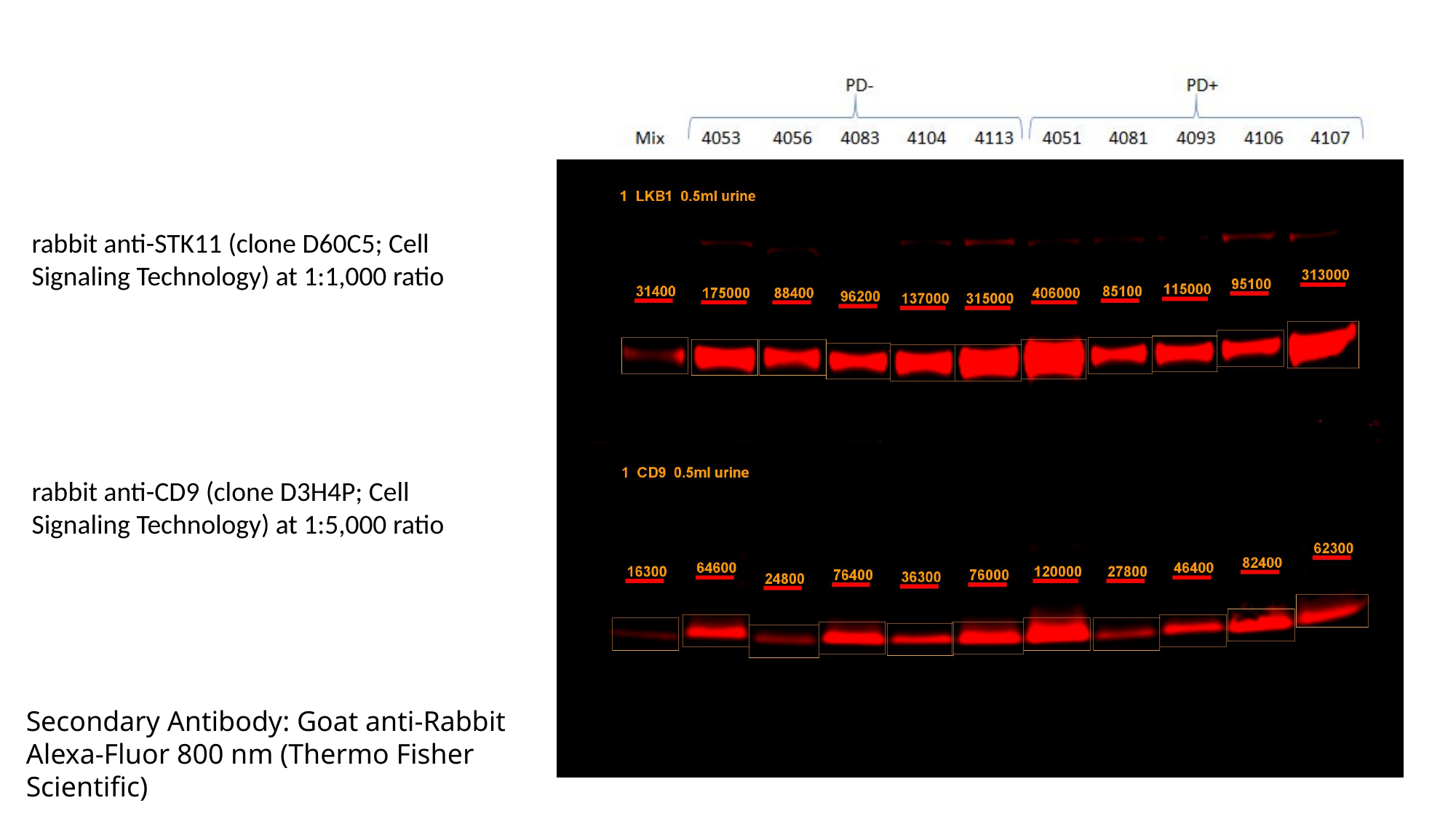

rabbit anti-STK11 (clone D60C5; Cell Signaling Technology) at 1:1,000 ratio
rabbit anti-CD9 (clone D3H4P; Cell Signaling Technology) at 1:5,000 ratio
Secondary Antibody: Goat anti-Rabbit Alexa-Fluor 800 nm (Thermo Fisher Scientific)

## Slide 5
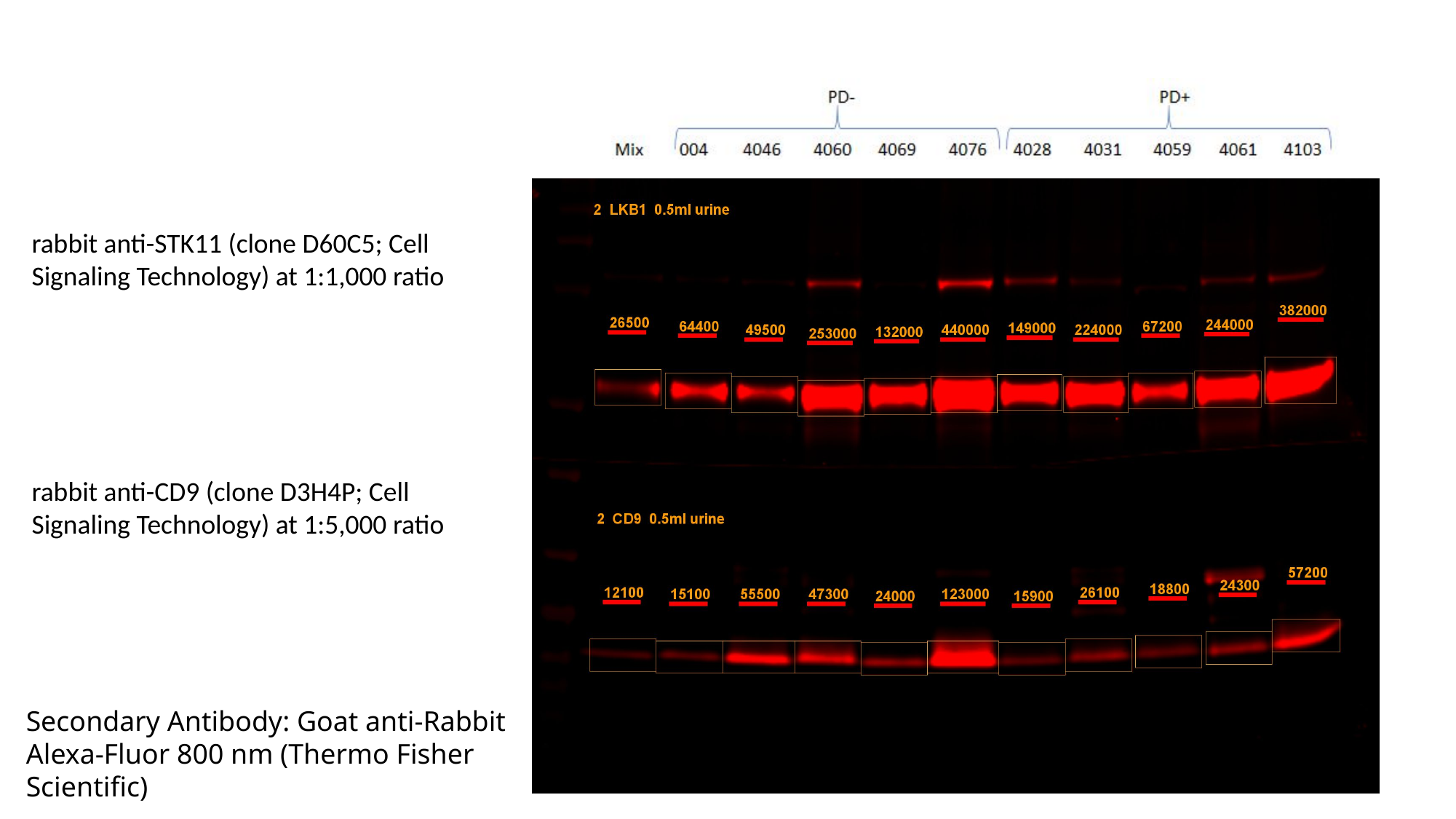

rabbit anti-STK11 (clone D60C5; Cell Signaling Technology) at 1:1,000 ratio
rabbit anti-CD9 (clone D3H4P; Cell Signaling Technology) at 1:5,000 ratio
Secondary Antibody: Goat anti-Rabbit Alexa-Fluor 800 nm (Thermo Fisher Scientific)

## Slide 6
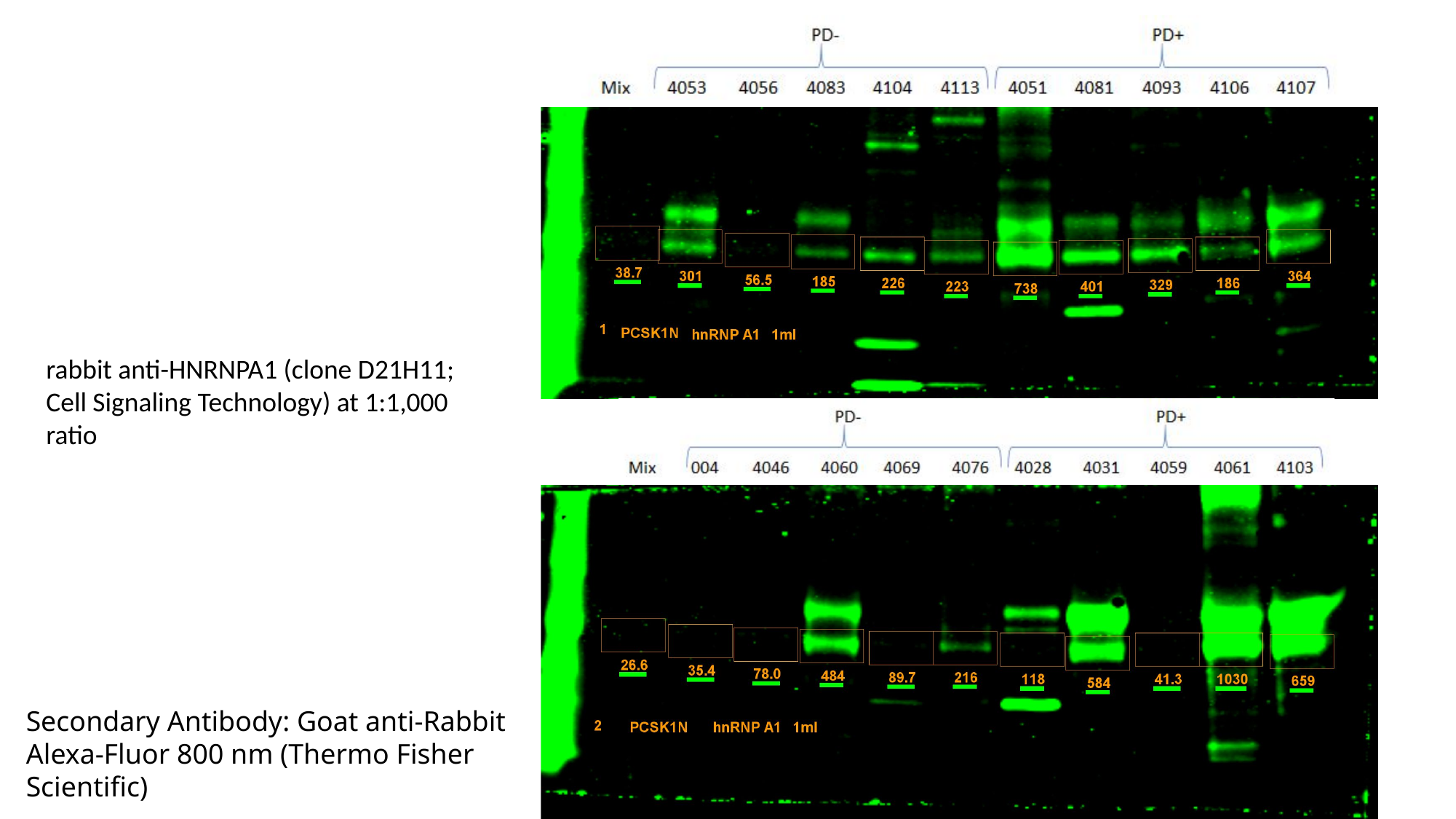

rabbit anti-HNRNPA1 (clone D21H11; Cell Signaling Technology) at 1:1,000 ratio
Secondary Antibody: Goat anti-Rabbit Alexa-Fluor 800 nm (Thermo Fisher Scientific)

## Slide 7
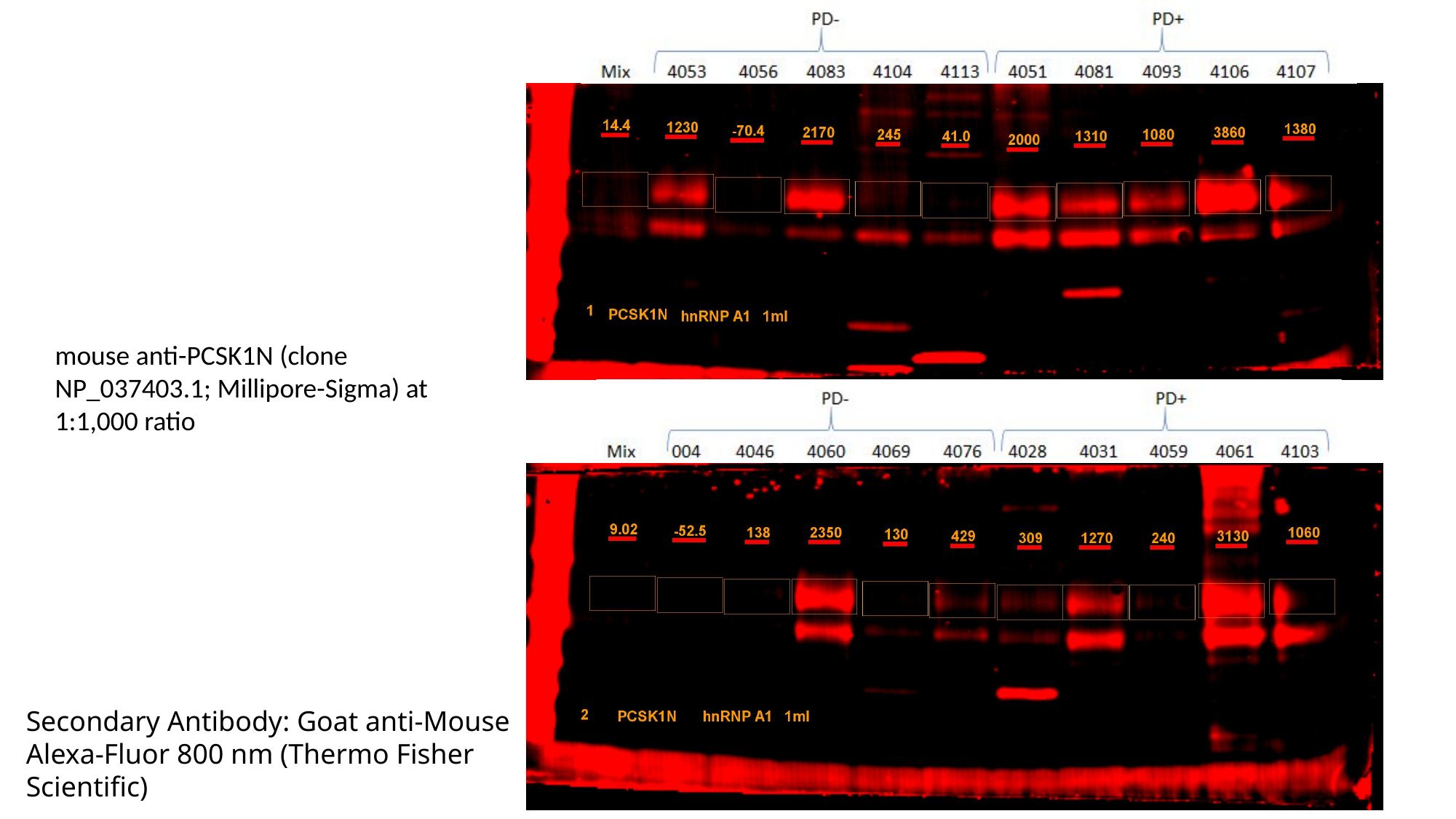

mouse anti-PCSK1N (clone NP_037403.1; Millipore-Sigma) at 1:1,000 ratio
Secondary Antibody: Goat anti-Mouse Alexa-Fluor 800 nm (Thermo Fisher Scientific)

## Slide 8
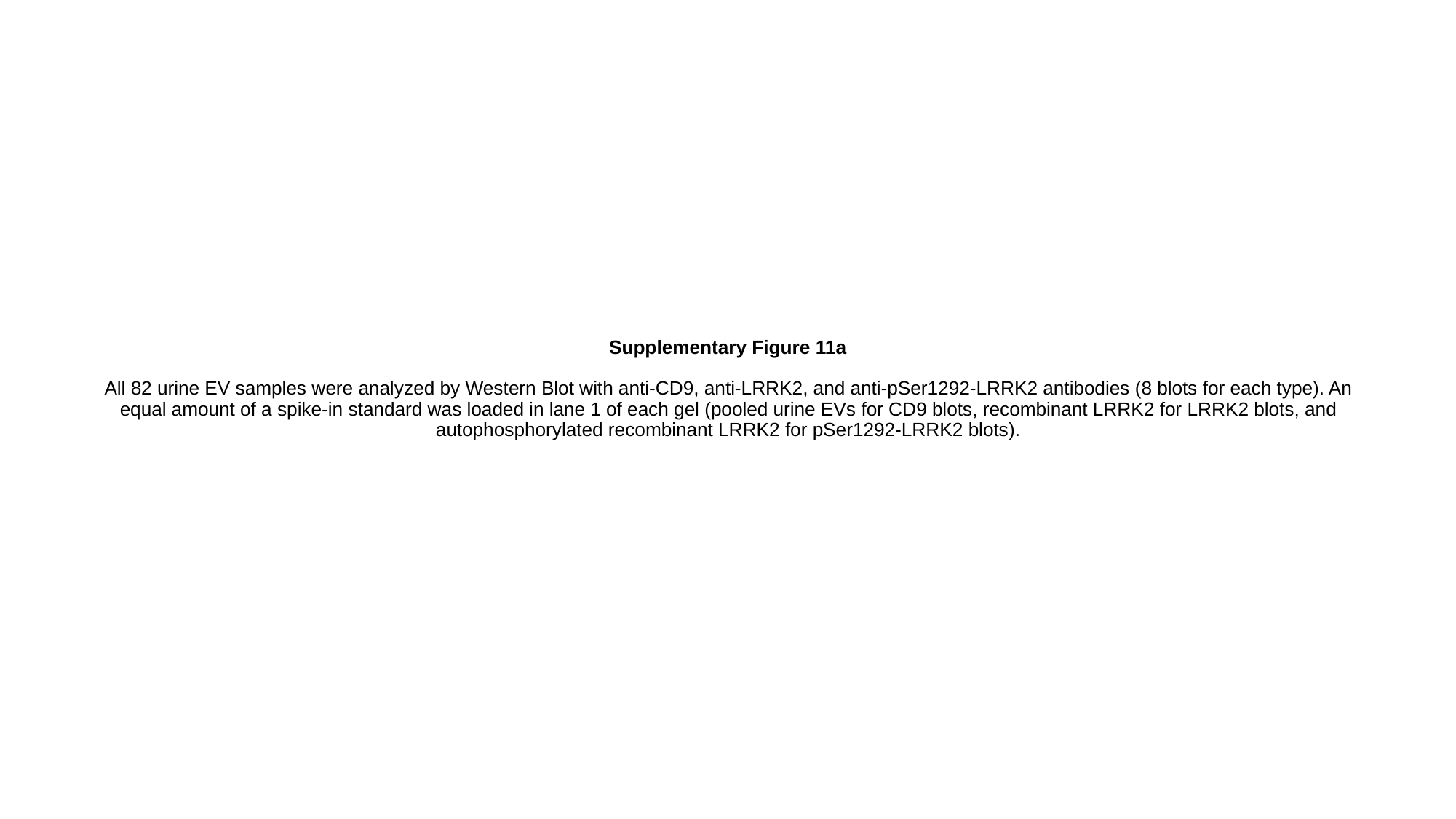

# Supplementary Figure 11a All 82 urine EV samples were analyzed by Western Blot with anti-CD9, anti-LRRK2, and anti-pSer1292-LRRK2 antibodies (8 blots for each type). An equal amount of a spike-in standard was loaded in lane 1 of each gel (pooled urine EVs for CD9 blots, recombinant LRRK2 for LRRK2 blots, and autophosphorylated recombinant LRRK2 for pSer1292-LRRK2 blots).

## Slide 9
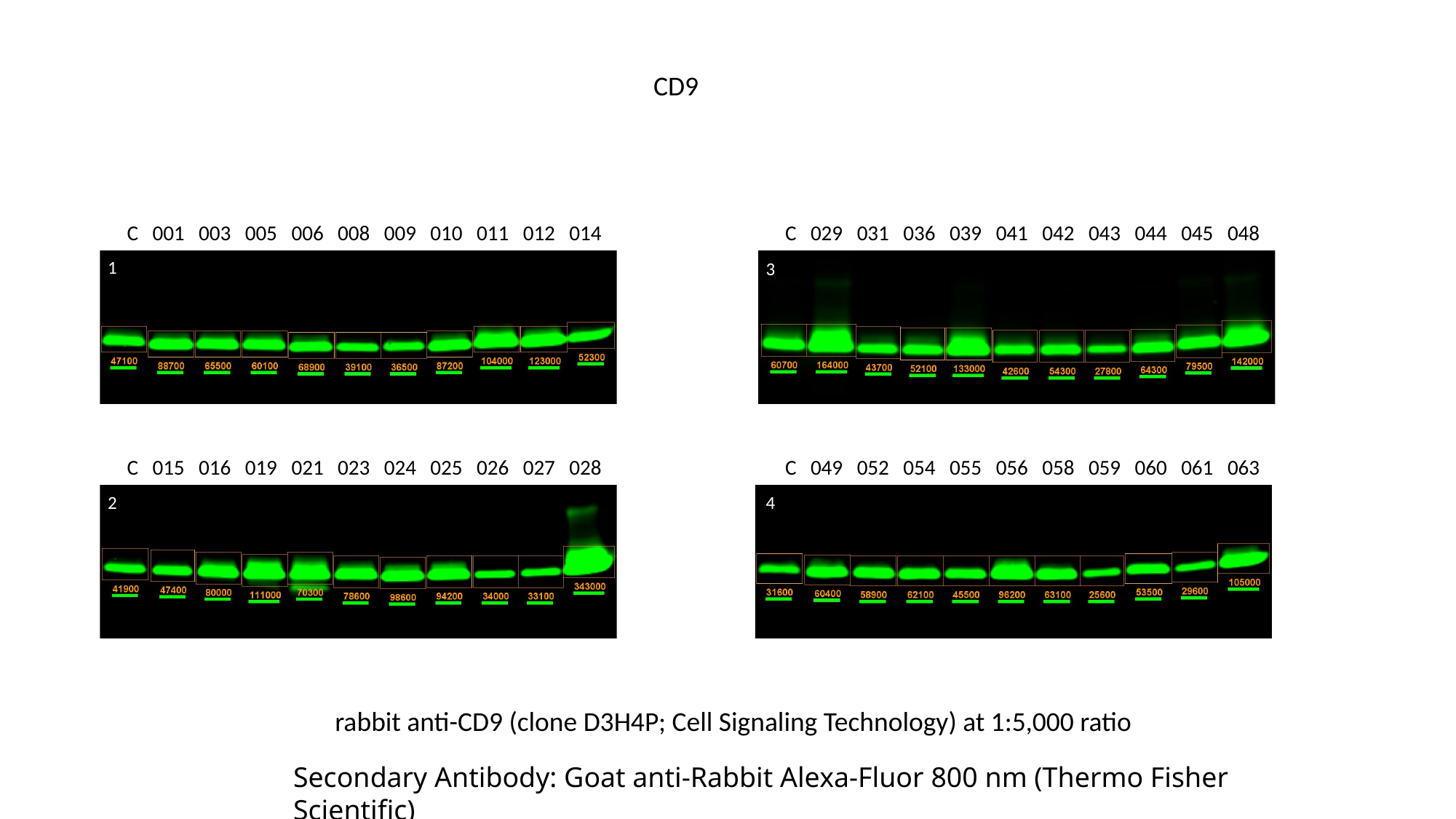

CD9
C 001 003 005 006 008 009 010 011 012 014
C 029 031 036 039 041 042 043 044 045 048
1
3
C 049 052 054 055 056 058 059 060 061 063
C 015 016 019 021 023 024 025 026 027 028
2
4
rabbit anti-CD9 (clone D3H4P; Cell Signaling Technology) at 1:5,000 ratio
Secondary Antibody: Goat anti-Rabbit Alexa-Fluor 800 nm (Thermo Fisher Scientific)

## Slide 10
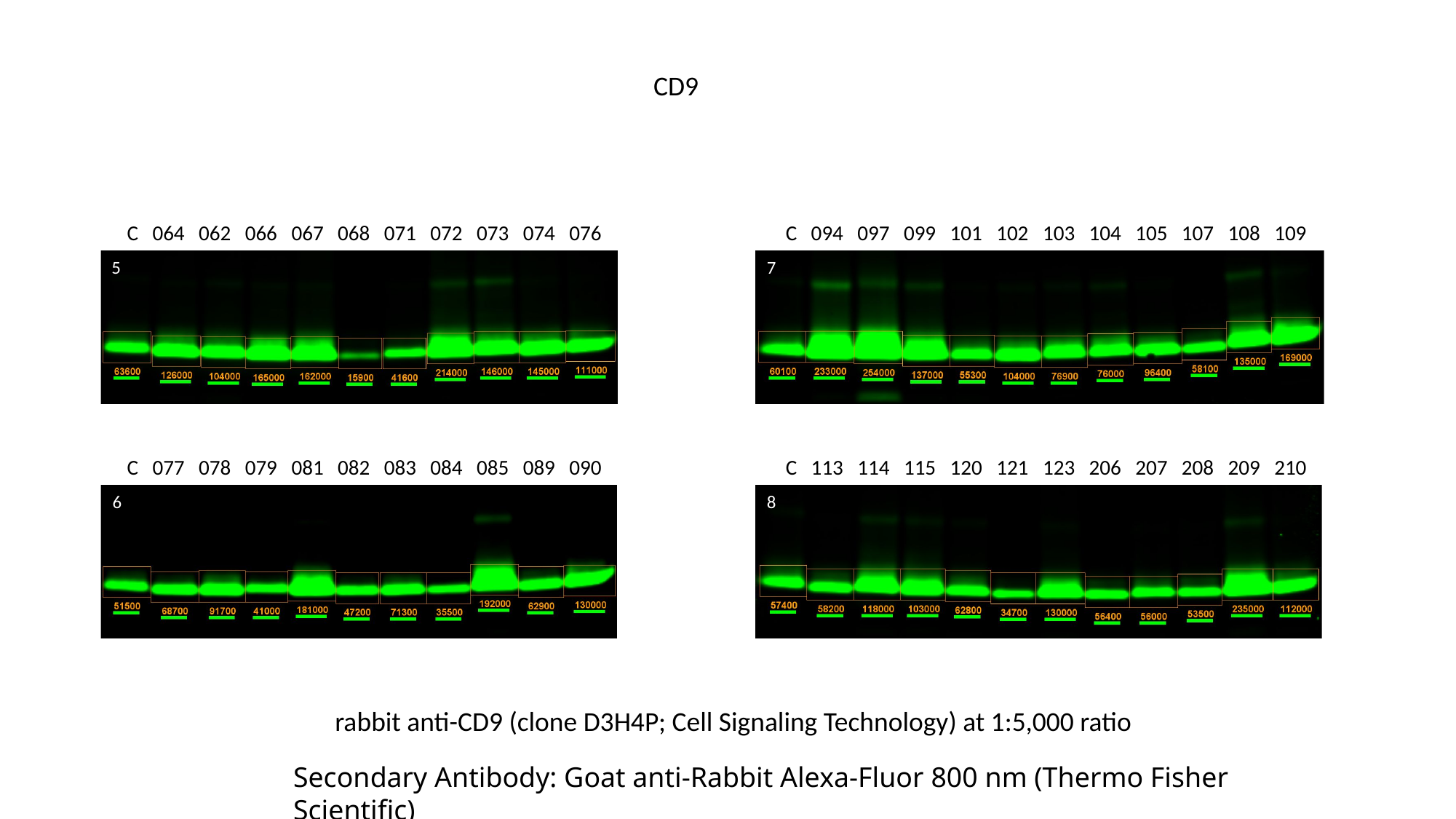

CD9
C 064 062 066 067 068 071 072 073 074 076
C 094 097 099 101 102 103 104 105 107 108 109
5
7
C 113 114 115 120 121 123 206 207 208 209 210
C 077 078 079 081 082 083 084 085 089 090
6
8
rabbit anti-CD9 (clone D3H4P; Cell Signaling Technology) at 1:5,000 ratio
Secondary Antibody: Goat anti-Rabbit Alexa-Fluor 800 nm (Thermo Fisher Scientific)

## Slide 11
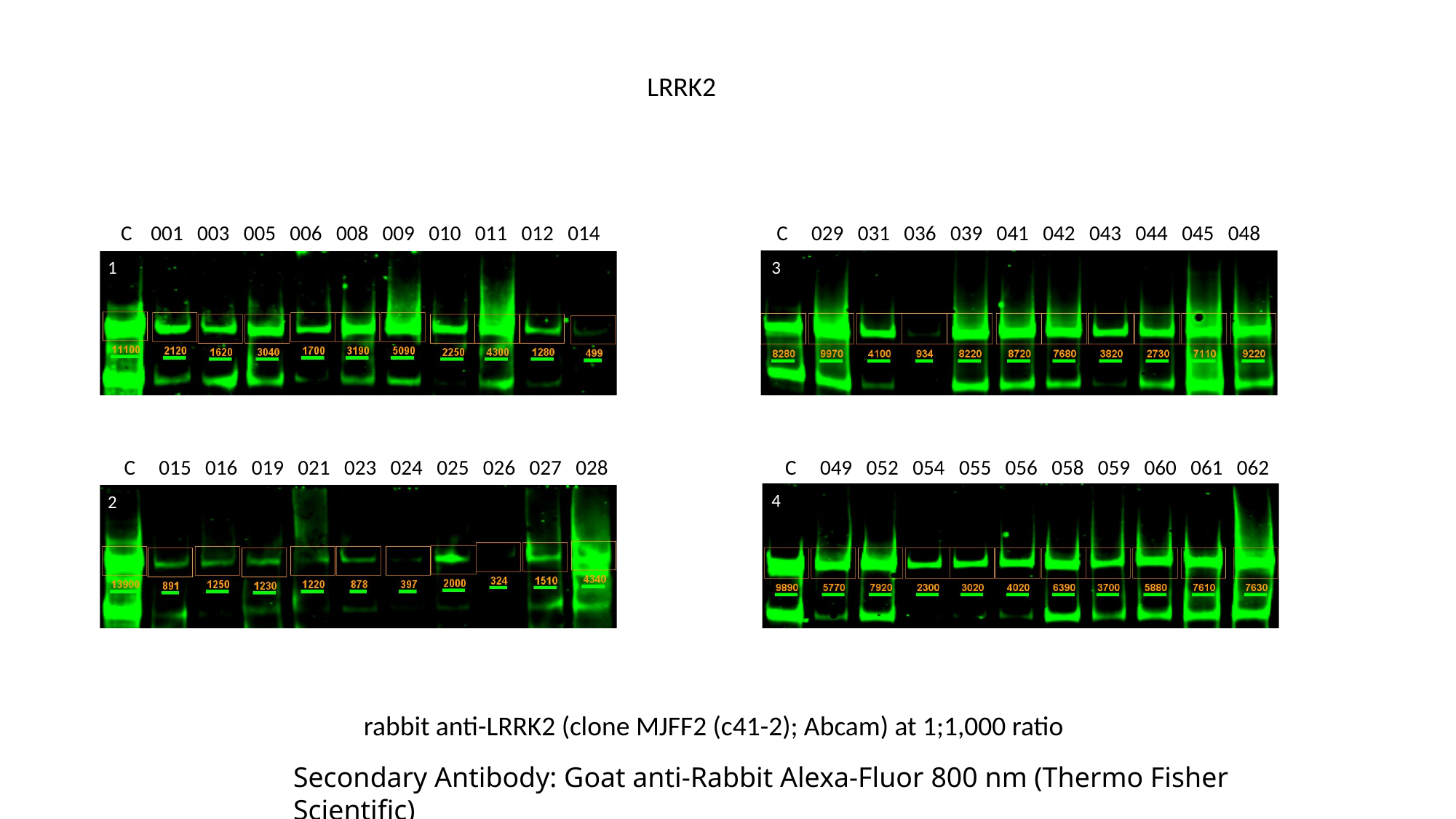

LRRK2
C 001 003 005 006 008 009 010 011 012 014
C 029 031 036 039 041 042 043 044 045 048
1
3
C 049 052 054 055 056 058 059 060 061 062
C 015 016 019 021 023 024 025 026 027 028
4
2
rabbit anti-LRRK2 (clone MJFF2 (c41-2); Abcam) at 1;1,000 ratio
Secondary Antibody: Goat anti-Rabbit Alexa-Fluor 800 nm (Thermo Fisher Scientific)

## Slide 12
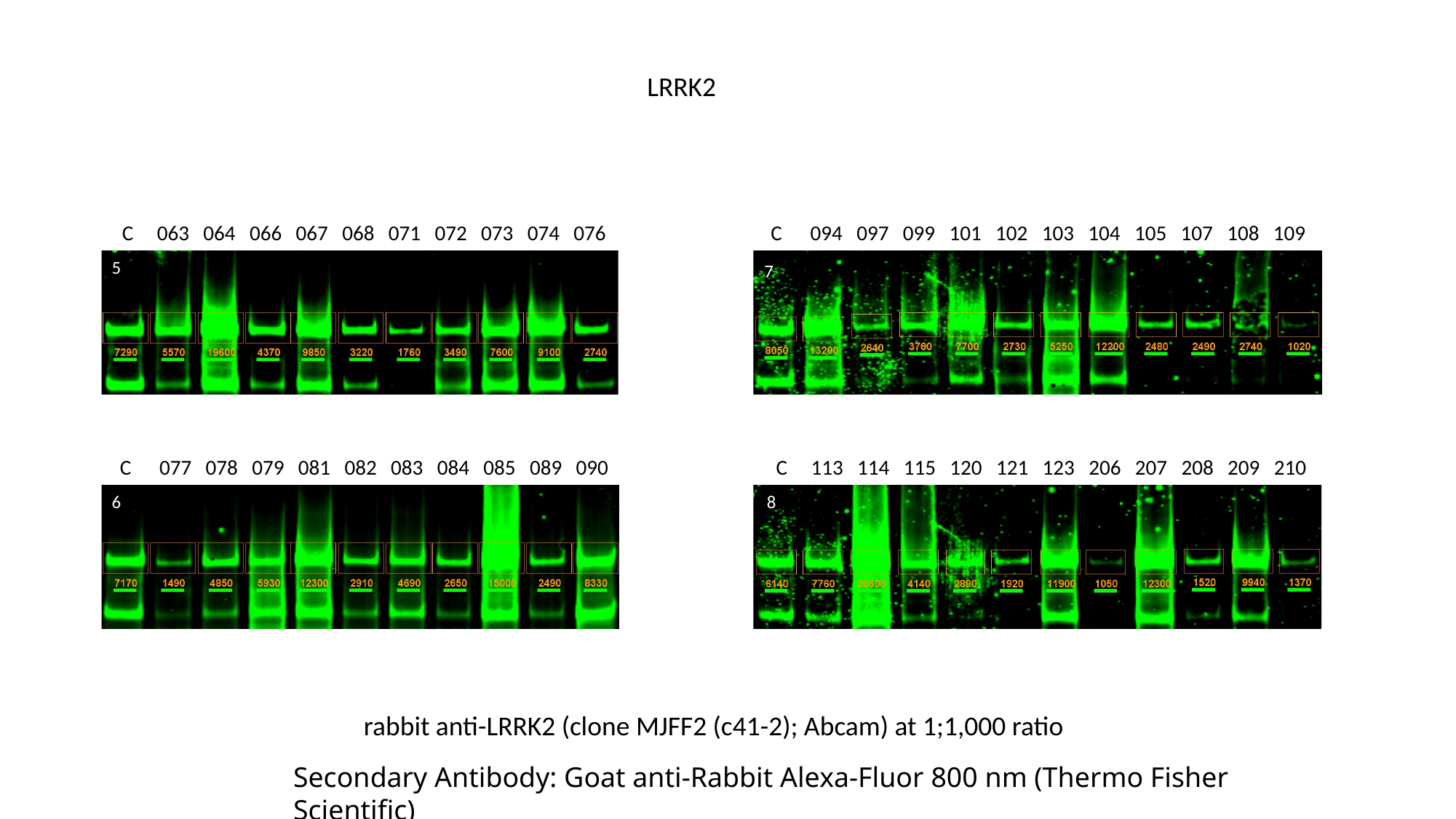

LRRK2
C 063 064 066 067 068 071 072 073 074 076
C 094 097 099 101 102 103 104 105 107 108 109
5
7
C 113 114 115 120 121 123 206 207 208 209 210
C 077 078 079 081 082 083 084 085 089 090
6
8
rabbit anti-LRRK2 (clone MJFF2 (c41-2); Abcam) at 1;1,000 ratio
Secondary Antibody: Goat anti-Rabbit Alexa-Fluor 800 nm (Thermo Fisher Scientific)

## Slide 13
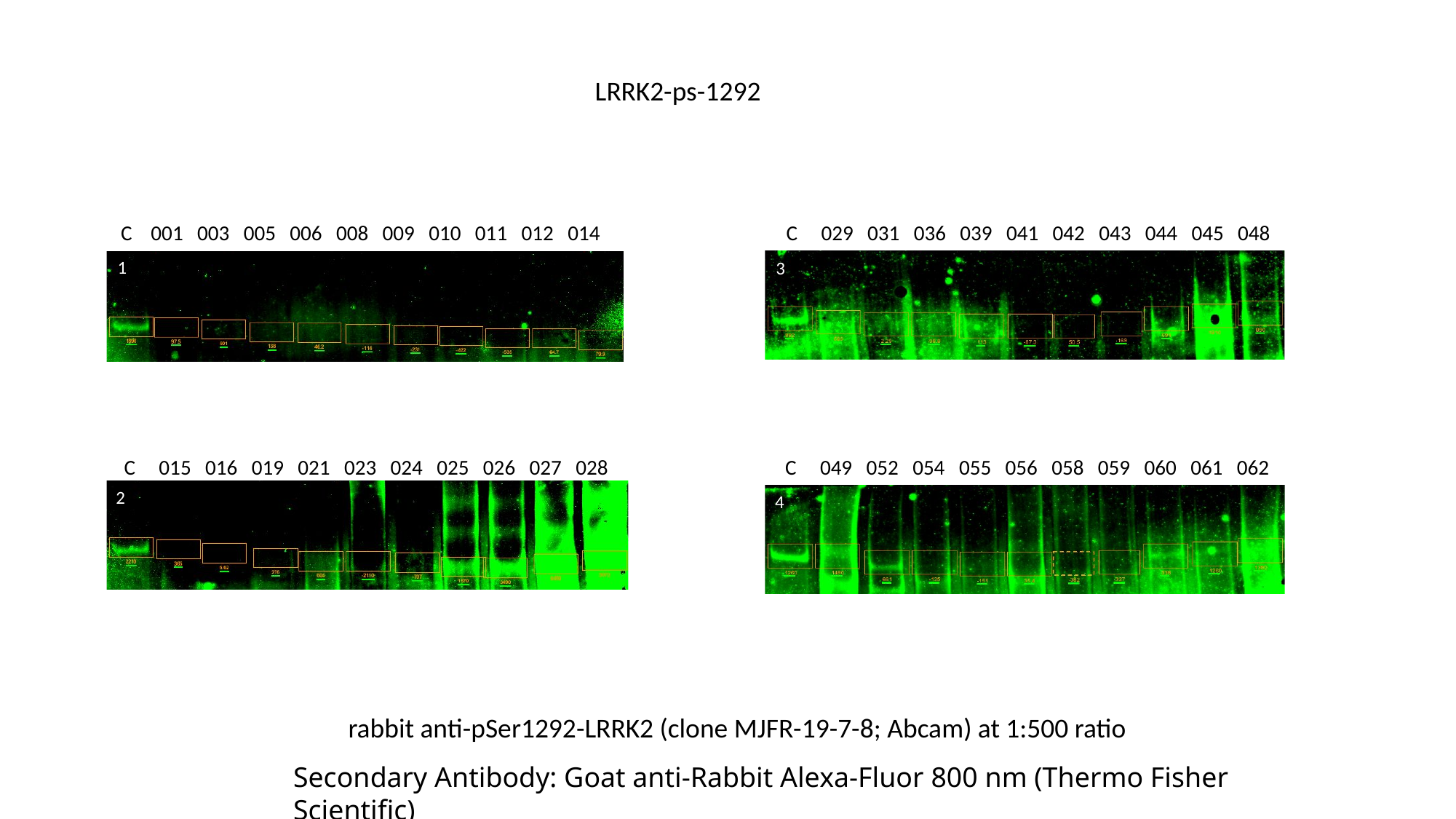

LRRK2-ps-1292
C 001 003 005 006 008 009 010 011 012 014
C 029 031 036 039 041 042 043 044 045 048
1
3
C 049 052 054 055 056 058 059 060 061 062
C 015 016 019 021 023 024 025 026 027 028
2
4
rabbit anti-pSer1292-LRRK2 (clone MJFR-19-7-8; Abcam) at 1:500 ratio
Secondary Antibody: Goat anti-Rabbit Alexa-Fluor 800 nm (Thermo Fisher Scientific)

## Slide 14
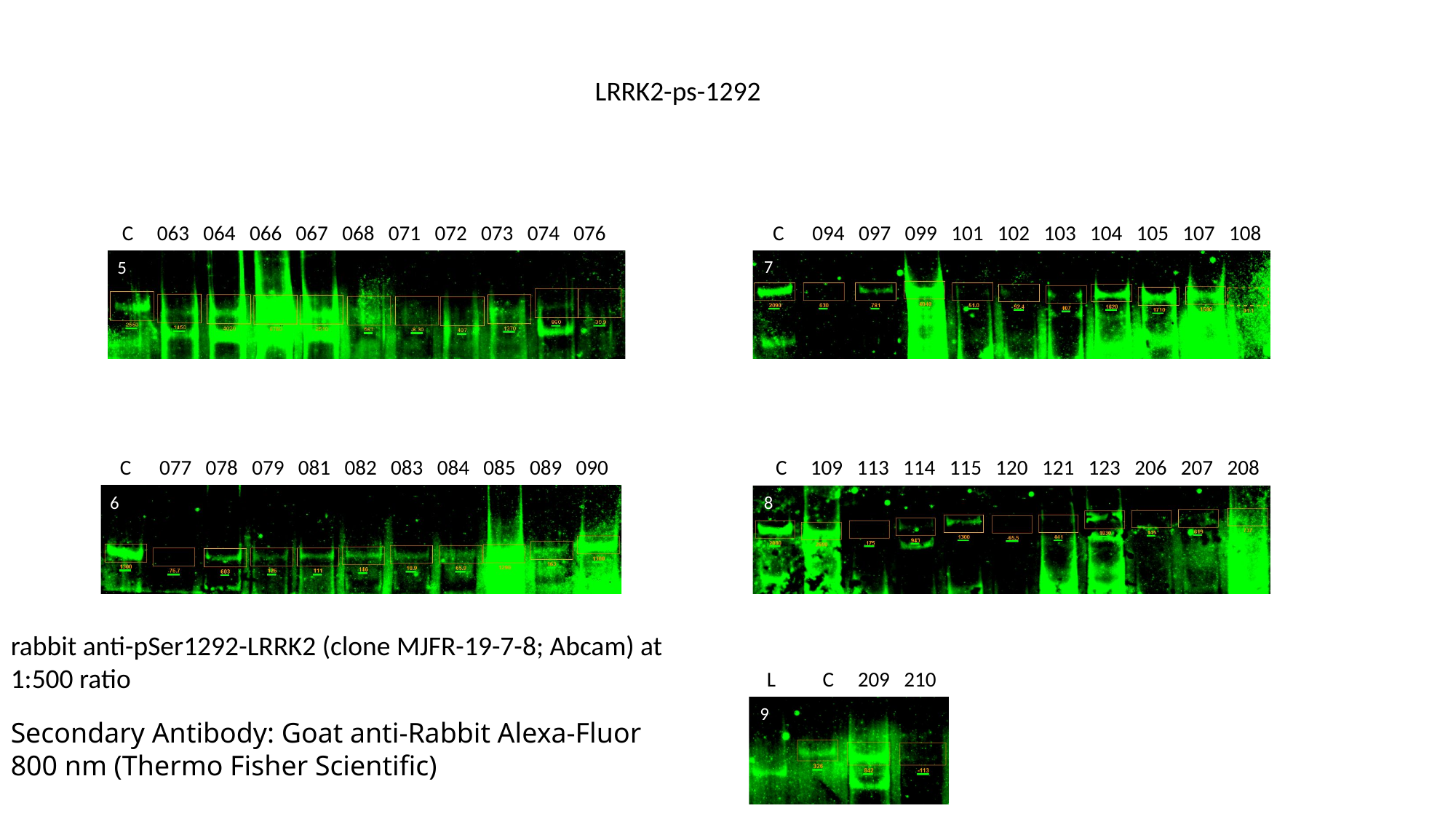

LRRK2-ps-1292
C 063 064 066 067 068 071 072 073 074 076
C 094 097 099 101 102 103 104 105 107 108
7
5
C 109 113 114 115 120 121 123 206 207 208
C 077 078 079 081 082 083 084 085 089 090
6
8
rabbit anti-pSer1292-LRRK2 (clone MJFR-19-7-8; Abcam) at 1:500 ratio
L C 209 210
9
Secondary Antibody: Goat anti-Rabbit Alexa-Fluor 800 nm (Thermo Fisher Scientific)
