# Supplementary material for: Quantitative proteomics and phosphoproteomics of urinary extracellular vesicles define putative diagnostic biosignatures for Parkinson’s disease
Source: Commun Med (Lond). 2023 May 10;3:64. doi: 10.1038/s43856-023-00294-w (PMC10172329; doi:10.1038/s43856-023-00294-w)
Supplement: Supplementary file 5 — Reporting Summary [file 43856_2023_294_MOESM5_ESM.pdf]

nature portfolio

Corresponding author(s): W. Andy Tao & Anton Iliuk

Last updated by author(s): Apr 3, 2023

Reporting Summary

Nature Portfolio wishes to improve the reproducibility of the work that we publish. This form provides structure for consistency and transparency in reporting. For further information on Nature Portfolio policies, see our Editorial Policies and the Editorial Policy Checklist.

Statistics

For all statistical analyses, confirm that the following items are present in the figure legend, table legend, main text, or Methods section.

n/a	Confirmed	
☐	☒	The exact sample size (*n*) for each experimental group/condition, given as a discrete number and unit of measurement
☐	☒	A statement on whether measurements were taken from distinct samples or whether the same sample was measured repeatedly
☐	☒	The statistical test(s) used AND whether they are one- or two-sided<br>*Only common tests should be described solely by name; describe more complex techniques in the Methods section.*
☒	☐	A description of all covariates tested
☐	☒	A description of any assumptions or corrections, such as tests of normality and adjustment for multiple comparisons
☐	☒	A full description of the statistical parameters including central tendency (e.g. means) or other basic estimates (e.g. regression coefficient) AND variation (e.g. standard deviation) or associated estimates of uncertainty (e.g. confidence intervals)
☐	☒	For null hypothesis testing, the test statistic (e.g. *F*, *t*, *r*) with confidence intervals, effect sizes, degrees of freedom and *P* value noted<br>*Give P values as exact values whenever suitable.*
☒	☐	For Bayesian analysis, information on the choice of priors and Markov chain Monte Carlo settings
☒	☐	For hierarchical and complex designs, identification of the appropriate level for tests and full reporting of outcomes
☐	☒	Estimates of effect sizes (e.g. Cohen's *d*, Pearson's *r*), indicating how they were calculated

Our web collection on statistics for biologists contains articles on many of the points above.

Software and code

Policy information about availability of computer code

Data collection	Xcalibur 4.1 was used for mass spectrometry data collection. The Western blot images were processed using Image Studio Studio Ver 5.2. The TRPS data was collected using Izon Control Suite 3.4.2.48.
Data analysis	Perseus 1.6.5.0<br>R 3.5.0<br>RStudio 2022.07.1 Build 554<br>ggplot2 3.3.1<br>ggpubr 0.3.0<br>EnhancedVolcano 1.7.6<br>Vennerable 3.0<br>Circlize 0.4.9<br>Cytoscape 3.8.0<br>python 3.8.8<br>conda 4.13.0<br>jupyter core 4.7.1<br>jupyter-notebook 6.3.0<br>pandas 1.4.3<br>numpy 1.20.1<br>matplotlib 3.3.4<br>plotly 5.6.0<br>sklearn 1.1.1

mlxtend 0.20.0
xgboost 1.6.1

For manuscripts utilizing custom algorithms or software that are central to the research but not yet described in published literature, software must be made available to editors and reviewers. We strongly encourage code deposition in a community repository (e.g. GitHub). See the Nature Portfolio guidelines for submitting code & software for further information.

Data

Policy information about availability of data

All manuscripts must include a data availability statement. This statement should provide the following information, where applicable:

- Accession codes, unique identifiers, or web links for publicly available datasets
- A description of any restrictions on data availability
- For clinical datasets or third party data, please ensure that the statement adheres to our policy

* Data Availability

The mass spectrometry raw data files and Proteome Discoverer search results for EVtrap repeatability, high-abundant free urine protein depletion, and the discovery experiments have been deposited in the MassIVE database (https://massive.ucsd.edu/ProteoSAFe/static/massive.jsp) and can be accessed via dataset identifier: MSV000085800 | PXD020475.

The PRM raw data files and the Skyline file have been deposited in the Panorama Public (https://panoramaweb.org/pd.url). The ProteomeXchange ID reserved for these data is: PXD032175 (http://proteomecentral.proteomexchange.org/cgi/GetDataset?ID=PXD032175).

The source data have been provided in files named "Source Data 1" and "Source Data 2" located inside the zipped "Source Data" folder.

* Code Availability

Custom Python code used for feature selections, model training, and predictive analyses, along with the README file, input files, and expected results have been deposited in Zenodo (https://doi.org/10.5281/zenodo.7679354).

Human research participants

Policy information about studies involving human research participants and Sex and Gender in Research.

Reporting on sex and gender	The sex information was obtained and presented in the main manuscript and the supplementary data. The data was collected during the sample collection based on self-reporting. The correlation study between several protein expression and sex was performed in this study.
Population characteristics	Analysis included age at recruitment, age at onset of PD, sex and PD-related genotype.
Recruitment	We recruited among patient and family members at Columbia University, with a focused recruitment of mutation carriers.
Ethics oversight	The study was approved by Columbia University Irving Medical Center institutional review board (IRB) no. AAAP9604, and all participants signed an informed consent. The study design and conduct complied with all relevant regulations regarding the use of human study participants and was conducted in accordance with the criteria set by the Declaration of Helsinki.

Note that full information on the approval of the study protocol must also be provided in the manuscript.

Field-specific reporting

Please select the one below that is the best fit for your research. If you are not sure, read the appropriate sections before making your selection.

☒ Life sciences ☐ Behavioural & social sciences ☐ Ecological, evolutionary & environmental sciences

For a reference copy of the document with all sections, see nature.com/documents/nr-reporting-summary-flat.pdf

Life sciences study design

All studies must disclose on these points even when the disclosure is negative.

Sample size	There was no sample size calculation performed. The sample size was determined based on the availability of samples at the start of this study. In total, we had 138 samples, where 82 urine samples were used in the discovery LC-MS study and 56 urine samples were used in the validation experiments. With these sample sizes, we discovered a robust set of biomarkers with very high AUC scores. Moreover, we could validate some of the urine EV biomarkers through PRM and Western Blot.
Data exclusions	No data were excluded from the analyses.

Replication	We validated the top biomarkers along with some other potential biomarkers. Some were successfully validated and some were not. Due to limited amount of urine samples, we could only target protein biomarkers. Some biomarkers also did not have ideal peptides to be targeted via PRM. Antibody based validation also was limited to antibody avaibility.
Randomization	One hundred thirty-eight unique subjects were divided randomly into the discovery and validation experiments. In the discovery experiment, the available 82 clinical urine samples were further randomly distributed into training and test sets for biomarker prediction.
Blinding	The research lab was blinded to PD status and genotype until releasing the results to the clinical group and the MJFF Foundation.

Reporting for specific materials, systems and methods

We require information from authors about some types of materials, experimental systems and methods used in many studies. Here, indicate whether each material, system or method listed is relevant to your study. If you are not sure if a list item applies to your research, read the appropriate section before selecting a response.

Materials & experimental systems

n/a	Involved in the study
☐	☒ Antibodies
☒	☐ Eukaryotic cell lines
☒	☐ Palaeontology and archaeology
☒	☐ Animals and other organisms
☐	☒ Clinical data
☒	☐ Dual use research of concern

Methods

n/a	Involved in the study
☒	☐ ChIP-seq
☒	☐ Flow cytometry
☒	☐ MRI-based neuroimaging

Antibodies

Antibodies used	rabbit anti-CD9 (clone D3H4P; Cell Signaling Technology) at 1:5,000 ratio<br>anti-LRRK2 (clone MJFF2 (c41-2); Abcam) at 1;1,000 ratio<br>anti-pSer1292-LRRK2 (clone MJFR-19-7-8; Abcam) at 1:500 ratio<br>rabbit anti-STK11 (clone D60C5; Cell Signaling Technology) at 1:1,000 ratio<br>mouse anti-PCSK1N (clone NP_037403.1; Millipore-Sigma) at 1:1,000 ratio<br>rabbit anti-HNRNPA1 (clone D21H11; Cell Signaling Technology) at 1:1,000 ratio<br>goat anti-Rabbit or goat anti-Mouse Alexa-Fluor 800 nm (Thermo Fisher Scientific)
Validation	The validation of each primary antibody is described in their associated publication, as referenced in the above section.<br>Validation documentation for rabbit anti-CD9 can be found at this webpage: https://www.cellsignal.com/products/primary-antibodies/cd9-d3h4p-rabbit-mab/13403<br>For anti-LRRK2: https://www.abcam.com/lrrk2-antibody-mjff2-c41-2-ab133474.html<br>For anti-pSer1292-LRRK2: https://www.abcam.com/lrrk2-phospho-s1292-antibody-mjfr-19-7-8-ab203181.html<br>For rabbit anti-STK11: https://www.cellsignal.com/products/primary-antibodies/lkb1-d60c5-rabbit-mab/3047<br>For mouse anti-PCSK1N: https://www.sigmaaldrich.com/US/en/product/sigma/sab1401647<br>For rabbit anti-HNRNPA1: https://www.cellsignal.com/products/primary-antibodies/hnrnp-a1-d21h11-rabbit-mab/8443

Clinical data

Policy information about clinical studies
All manuscripts should comply with the ICMJE guidelines for publication of clinical research and a completed CONSORT checklist must be included with all submissions.

Clinical trial registration	This is not a clinical trial and was not reported to clinicaltrials.gov.
Study protocol	This was a sample collection protocol. The protocol is available in multiple publications from this recruitment. e.g., https://pubmed.ncbi.nlm.nih.gov/32652692/
Data collection	All 82 urine samples used in the discovery LC-MS study and 56 urine samples used in the validation experiments were collected at Columbia University Irving Medical Center (CUIMC) and sent to our lab blindly. The samples were collected from March 2016 to April 2017 under a Michael J. Fox Foundation (MJFF)-funded LRRK2 biomarker project.
Outcomes	We expect our immediate results, followed by extensive evaluation and validation of the new markers in the clinical settings, could improve PD patients' medical outcomes and quality of life.
